# Two *Phytophthora parasitica* cysteine protease genes, *PpCys44* and *PpCys45*, trigger cell death in various *Nicotiana* spp. and act as virulence factors

**DOI:** 10.1111/mpp.12915

**Published:** 2020-02-19

**Authors:** Qiang Zhang, Weiwei Li, Jiapeng Yang, Junjie Xu, Yuling Meng, Weixing Shan

**Affiliations:** ^1^ State Key Laboratory of Crop Stress Biology for Arid Areas Northwest A&F University Yangling China; ^2^ College of Plant Protection Northwest A&F University Yangling China; ^3^ College of Agronomy Northwest A&F University Yangling China

**Keywords:** cell death, cysteine protease, *Phytophthora parasitica*, virulence factor

## Abstract

Proteases secreted by pathogens have been shown to be important virulence factors modifying plant immunity, and cysteine proteases have been demonstrated to participate in different pathosystems. However, the virulence functions of the cysteine proteases secreted by *Phytophthora parasitica* are poorly understood. Using a publicly available genome database, we identified 80 cysteine proteases in *P. parasitica*, 21 of which were shown to be secreted. Most of the secreted cysteine proteases are conserved among different *P. parasitica* strains and are induced during infection. The secreted cysteine protease proteins PpCys44/45 (proteases with identical protein sequences) and PpCys69 triggered cell death on the leaves of different *Nicotiana* spp. A truncated mutant of PpCys44/45 lacking a signal peptide failed to trigger cell death, suggesting that PpCys44/45 functions in the apoplastic space. Analysis of three catalytic site mutants showed that the enzyme activity of PpCys44/45 is required for its ability to trigger cell death. A virus‐induced gene silencing assay showed that PpCys44/45 does not induce cell death on *NPK1* (*Nicotiana Protein Kinase 1*)‐silenced *Nicotiana benthamiana* plants, indicating that the cell death phenotype triggered by PpCys44/45 is dependent on *NPK1*. PpCys44‐ and PpCys45‐deficient double mutants showed decreased virulence, suggesting that PpCys44 and PpCys45 positively promote pathogen virulence during infection. *PpCys44* and *PpCys45* are important virulence factors of *P. parasitica* and trigger *NPK1‐*dependent cell death in various *Nicotiana* spp.

## Graphical abstract

The oomycete pathogen *Phytophthora parasitica* employs cysteine proteases PpCys44/45 to enhance its virulence by inducing *NPK1‐*dependent cell death in different *Nicotiana* spp.

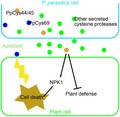

## 1 INTRODUCTION

Proteolysis of plant substrates is a strategy employed by pathogens to alter plant physiology (Hotson and Mudgett, 2004). Many enzyme effectors secreted by pathogens have been shown to manipulate plant immunity, such as the glycoside hydrolase 12 protein XEG1 (Ma *et al.*, 2015, 2017) from *Phytophthora sojae*; small phospholipase D‐like proteins (Meijer *et al.*, 2019) and serine proteases (Paris and Lamattina, 1999) from *Phytophthora infestans*; and metalloproteases, subtilisin, aspartic proteases, aspartyl acid proteases, non‐aspartyl acid proteases, and cysteine proteases from plant pathogenic fungi (Chandrasekaran *et al.*, 2016) and bacteria (Hotson and Mudgett, 2004; Potempa and Pike, 2009; Niño *et al.*, 2014).

In the MEROPS database (https://www.ebi.ac.uk) all the cysteine proteases from different organisms are classified into 15 clans and several unassigned families. Cysteine proteases from different families play different roles in plant pathosystems (Niño *et al.*, 2014). In plants, cysteine proteases function in resistance against pathogens. In *Arabidopsis thaliana*, cathepsin B proteins contribute to cell death and are involved in plant basal resistance against *Pseudomonas syringae* pv. *tomato* DC3000 (McLellan *et al.*, 2009). The *A. thaliana* cysteine protease RD19 was shown to be relocalized from vesicles to the nucleus by PopP2, a *Ralstonia solanacearum* effector, to form a complex that is required for RESISTANT TO RALSTONIA SOLANACEARUM 1‐R (RRS1‐R)‐mediated resistance (Bernoux *et al.*, 2008). The extracellular tomato cysteine protease Rcr3 is bound and inhibited by the *Cladosporium fulvum* effector Avr2, and the Rcr3/Avr2 complex can trigger cell death (Rooney, 2005). A *Nicotiana benthamiana* C14 cysteine protease interacts with the *P. infestans* effectors EPIC1, EPIC2B, and AVRblb2, and contributes to resistance against this pathogen (Kaschani *et al.*, 2010; Bozkurt *et al*, 2011).

In contrast to cysteine proteases in plants, those secreted by plant pathogens interfere with plant immunity. For example, AvrPphB from *P. syringae* is modified in the plant cell (Puri *et al.*, 1997) and promotes *P. syringae* colonization by suppressing the activation of the *A. thaliana* R protein RPM1 (Russell *et al.*, 2015). AvrRpt2 from *P. syringae* degrades RIN4 and induces RPS2‐mediated resistance. In the absence of RPS2, AvrRpt2 also interferes with the AvrRpm1/Rpm1‐mediated defence response (Hotson and Mudgett, 2004). As effectors share similar protease domains with host protease proteins, some effector proteases mimic those from plants to modify plant immunity. The *Xanthomonas* effector AvrXv4 possesses SUMO‐isopeptidase activity and significantly reduces the level of SUMO‐protein conjugates when it is heterologously expressed in plant cells (Roden *et al.*, 2004). The *Xanthomonas* XopD effector, a cysteine protease possessing both SUMO peptidase and isopeptidase activity, also mimics plant SUMO proteases to modify downstream plant proteins (Hotson *et al.*, 2003).

*Phytophthora parasitica* is a typical hemibiotrophic plant pathogen with a wide host spectrum (Meng *et al.*, 2014; Kamoun *et al.*, 2015). At the early stages of infection, *P. parasitica* evades or suppresses programmed cell death; however, late in the infection process it triggers cell death, causing disease lesions, which may promote pathogen colonization. *P. parasitica* secretes an arsenal of “weapons” to manipulate plant immunity. The elicitin ParA1 (Kamoun *et al.*, 1993) and the NEP1‐like protein NPP1 (Fellbrich *et al.*, 2002) from *P. parasitica* were shown to trigger hypersensitive cell death on plant leaves. The RXLR effector PSE1 suppresses plant immunity by modulating auxin accumulation during infection (Evangelisti *et al.*, 2013). Another RXLR effector PpE4, which is highly expressed early in infection, elicits strong cell death and contributes to pathogen virulence (Huang *et al.*, 2019). However, the molecular mechanism by which *P. parasitica* infects plants is largely unclear. There are many uncharacterized proteolytic enzymes annotated in the *P. parasitica* genome, and whether or not they participate in pathogen infection is still unknown. In this study, we identified 80 cysteine proteases from *P. parasitica* and analysed the sequence conservation and expression pattern of those predicted to be secreted. PpCys44 and PpCys45, which are up‐regulated during the late stages of infection and trigger cell death in *N. benthamiana* leaves, were further investigated.

## 2 RESULTS

### 2.1 *P. parasitica* encodes numerous cysteine proteases

Considering that cysteine proteases have been identified as virulence factors in bacteria (Shao *et al.*, 2002; Dowen *et al.*, 2009) and *Leishmania* (Descoteaux, 1998), we aimed to investigate the distribution and function of cysteine proteases in *P. parasitica*. Based on the publicly available *P. parasitica* INRA‐310 genome, 80 candidate genes encoding cysteine proteases were identified (Supporting Information S1) and divided into 12 families (Supporting Information S2). C48 is the largest family with 35 members, the C1 family has 20 members, and both the C2 and OTU‐like families have 7 members. Each of the other families has fewer than three members.

**Figure 1:**
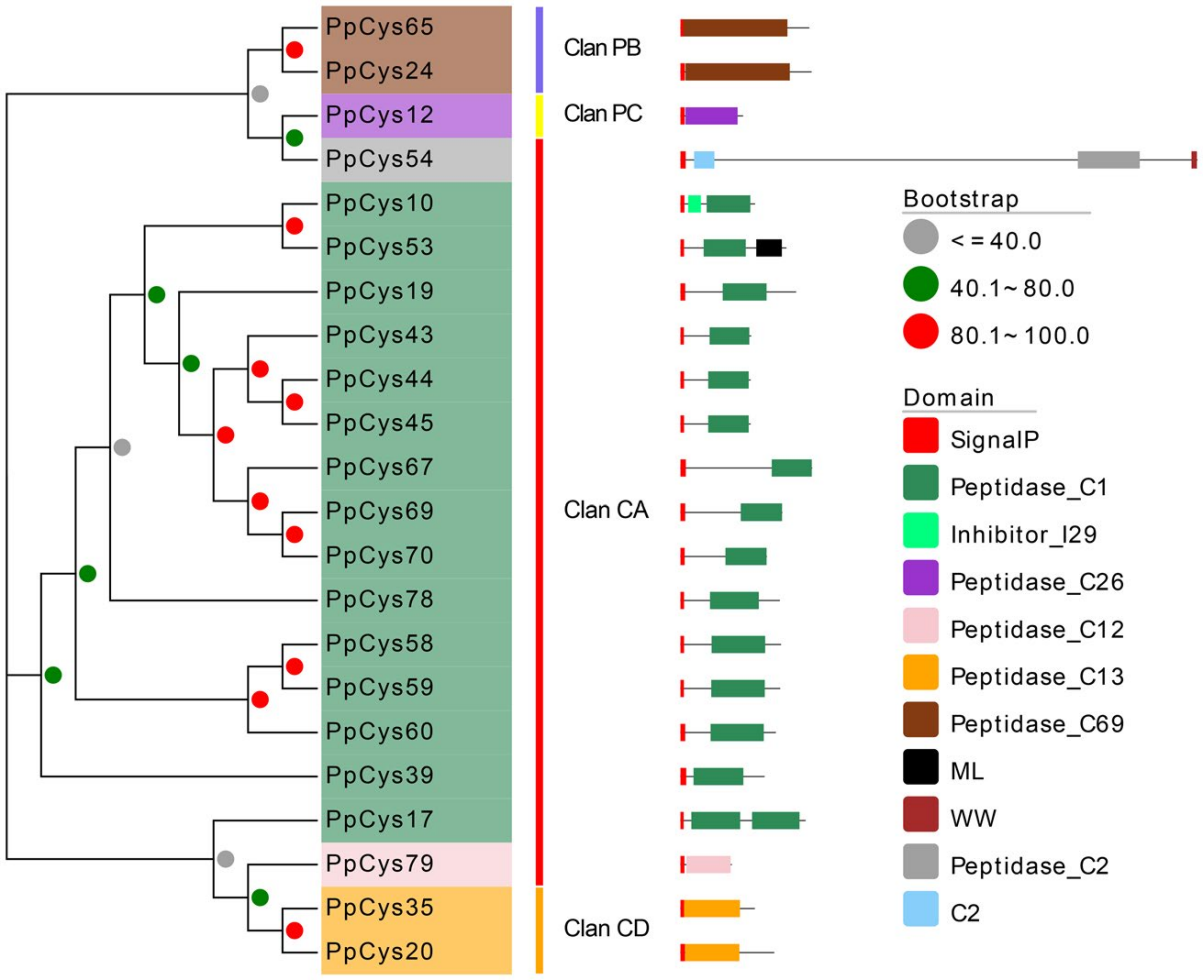
Phylogenetic analysis of the predicted secreted cysteine proteases of *Phytophthora parasitica* and analysis of their domain architectures. Phylogenetic analysis of secreted cysteine protease proteins was performed with MEGA X using the maximum‐likelihood method. The coloured dots near the nodes of the branches represent the percentage of replicate trees in which the associated taxa clustered together in the bootstrap test (1,000 replicates). The colours of each protein in the phylogenetic tree represent the cysteine protease families they belong to: brown, C69; purple, C26; grey, C2; sea green, C1; pink, C12; orange, C13. Coloured rectangles in the schematic diagrams of protein structures represent the different domains in each protein. These domains were predicted using NCBI's Conserved Domain Database (Marchler‐Bauer *et al*., 2017)

**Figure 2:**
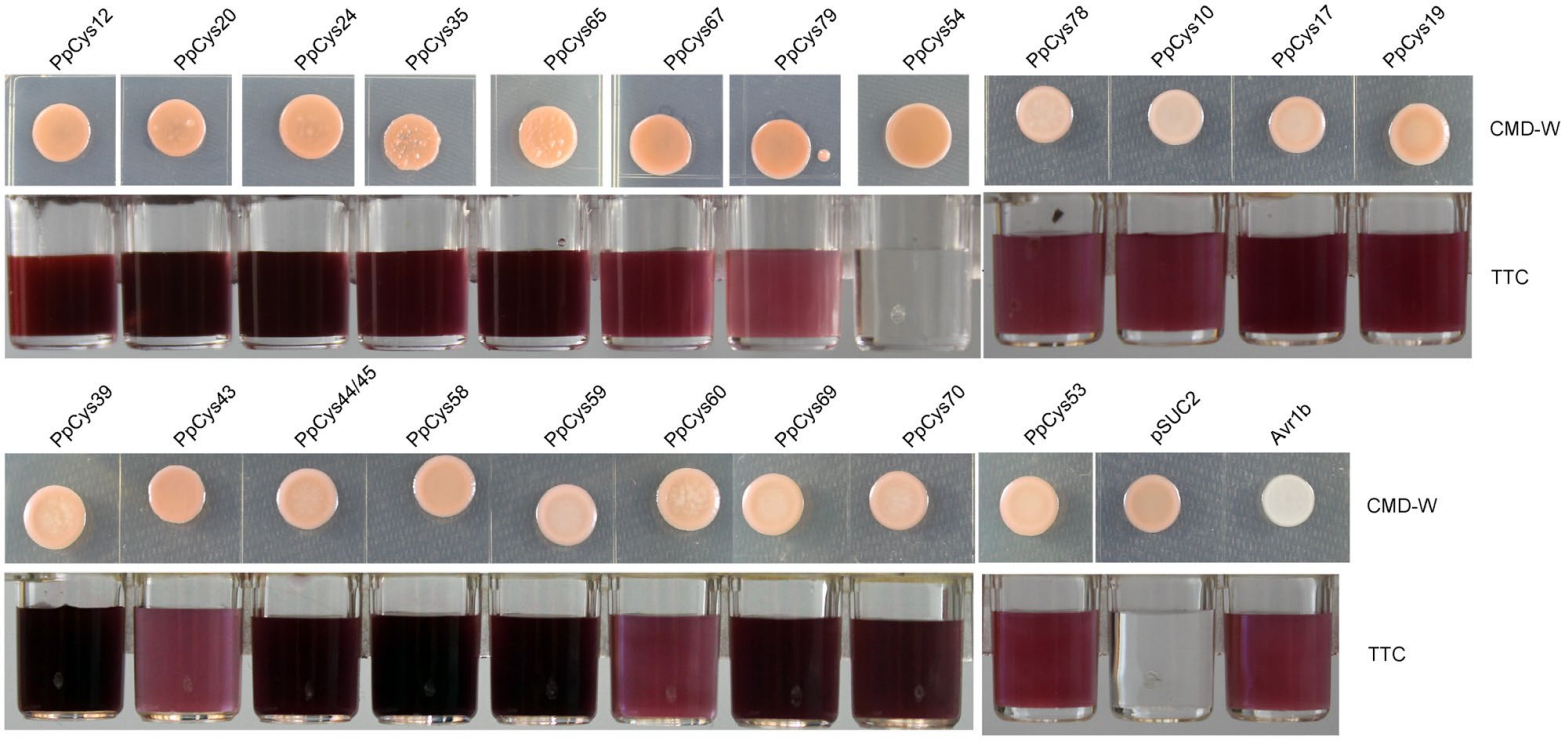
Functional analysis of predicted signal peptides using a yeast secretion system. Upper row photographs show yeast strains expressing the signal peptides from cysteine proteases fused with invertase grown on a CMD‐W agar plate. Lower photographs show the reactions with triphenyltetrazolium chloride (TTC) solution; a colour change indicates the conversion of TTC to triphenylformazan catalysed by secreted invertase. A yeast strain carrying a construct expressing the Avr1b signal peptide fused with invertase was used as a positive control. A yeast strain carrying the empty vector (pSUC2) was used as a negative control

Virulence factors secreted by pathogens usually act in the extracellular space or enter the plant cell, therefore we also performed in silico prediction of the signal peptides of the candidate cysteine proteases. Twenty‐two proteins have a predicted N‐terminal signal peptide (Figure 1), most (19) of which belong to the clan CA (C1, C2, and C12 families) and clan CD (C13 family). To confirm that the predicted signal peptides are indeed functional, we tested the ability of the signal peptides to direct secretion of invertase using a yeast secretion system. All the strains expressing fusions of the predicted cysteine protease signal peptides with invertase (except for *PpCys54*) could reduce triphenyltetrazolium chloride (TTC) to the insoluble red compound triphenylformazan (TPF), indicating that the signal peptides are indeed functional. The yeast strain expressing a functional Avr1b signal peptide fused with invertase was used as a positive control (Dou *et al.*, 2008; Song *et al.*, 2015). In contrast, no colour change was observed for the control strain expressing the empty vector (Figure 2).

Cysteine proteases usually have a prodomain that prevents unwanted protein degradation by preventing access of the substrate to the catalytic domain (Coulombe *et al.*, 1996). However, most secreted cysteine proteases in *P. parasitica* did not have the N‐terminal auto‐inhibitory domain and only had a predicted catalytically active peptidase domain (Figure 1).

### 2.2 Most predicted secreted cysteine proteases are conserved in *P. parasitica* and induced during infection

**Figure 3:**
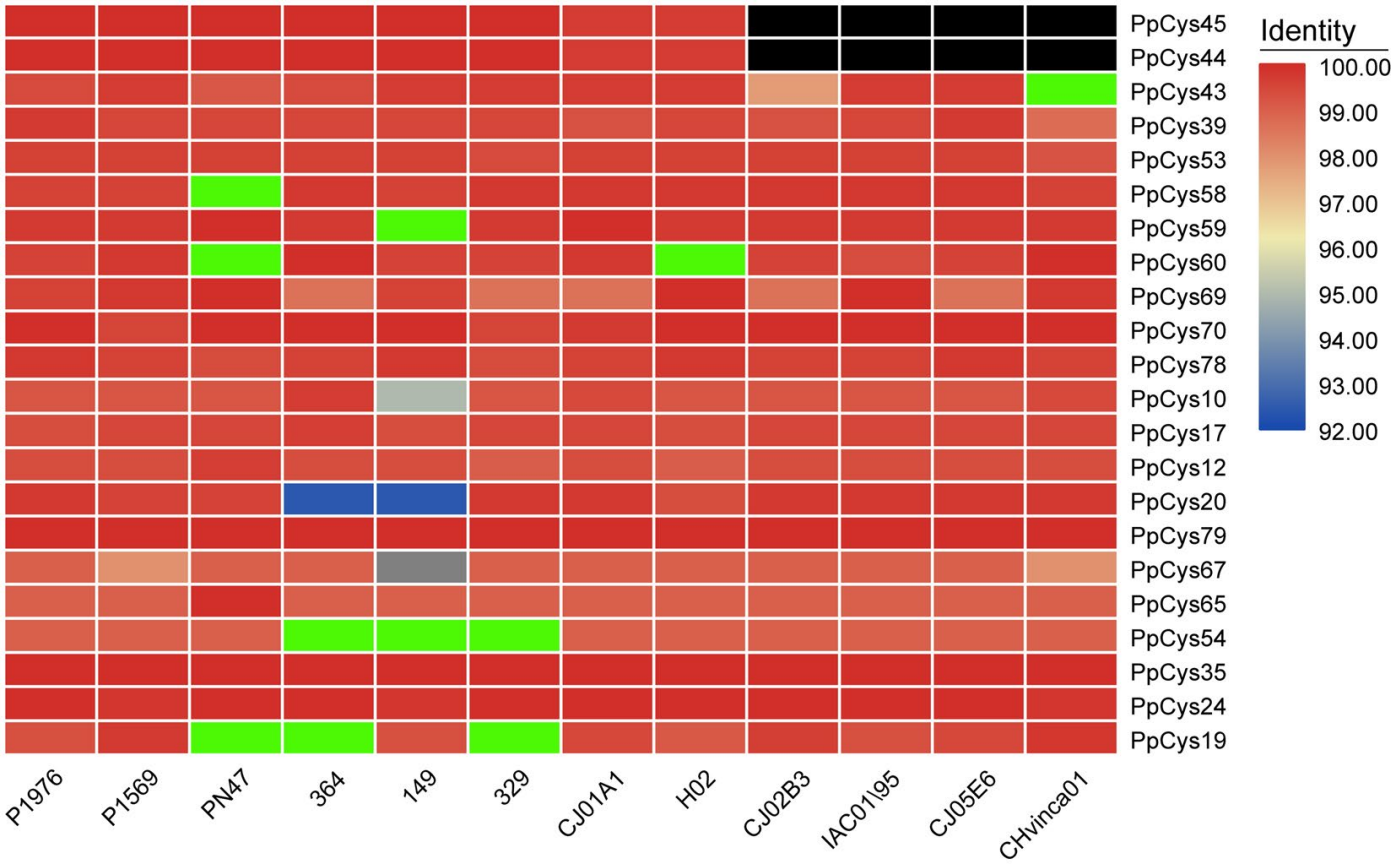
The secreted cysteine proteases are conserved among different sequenced *Phytophthora parasitica* strains*.* P1976, P1569, PN47, 364, 149, 329, CJ01A1, H02, CJ02B3, IAC01/95, CJ05E6, and CHvinca01 are the *P. parasitica* strains whose genomes have been sequenced. The colours in this heatmap represent the percentage identity between genes in each strain compared with those in the reference strain INRA‐310. Bright green indicates that the gene sequence is split between different contigs. Black indicates that the gene is absent in a strain

To investigate the similarity between predicted secreted cysteine proteases from different *P. parasitica* strains, we compared the protein sequences from reference strain INRA‐310 with those from the other 12 sequenced *P. parasitica* strains. All the proteases were found in nearly all the *P. parasitica* strains tested, and the sequence similarities were higher than 90%, indicating their high level of conservation among different *P. parasitica* strains (Figure 3). We also analysed the sequence similarities between cysteine proteases from different *Phytophthora* species, i.e., *P. sojae* and *P. infestans*. We identified orthologs of all 22 predicted secreted proteases in the *P. infestans* genome and orthologs of 16 proteins in the *P. sojae* genome, indicating their conservation among different *Phytophthora* species (Supporting Information S3).

**Figure 4:**
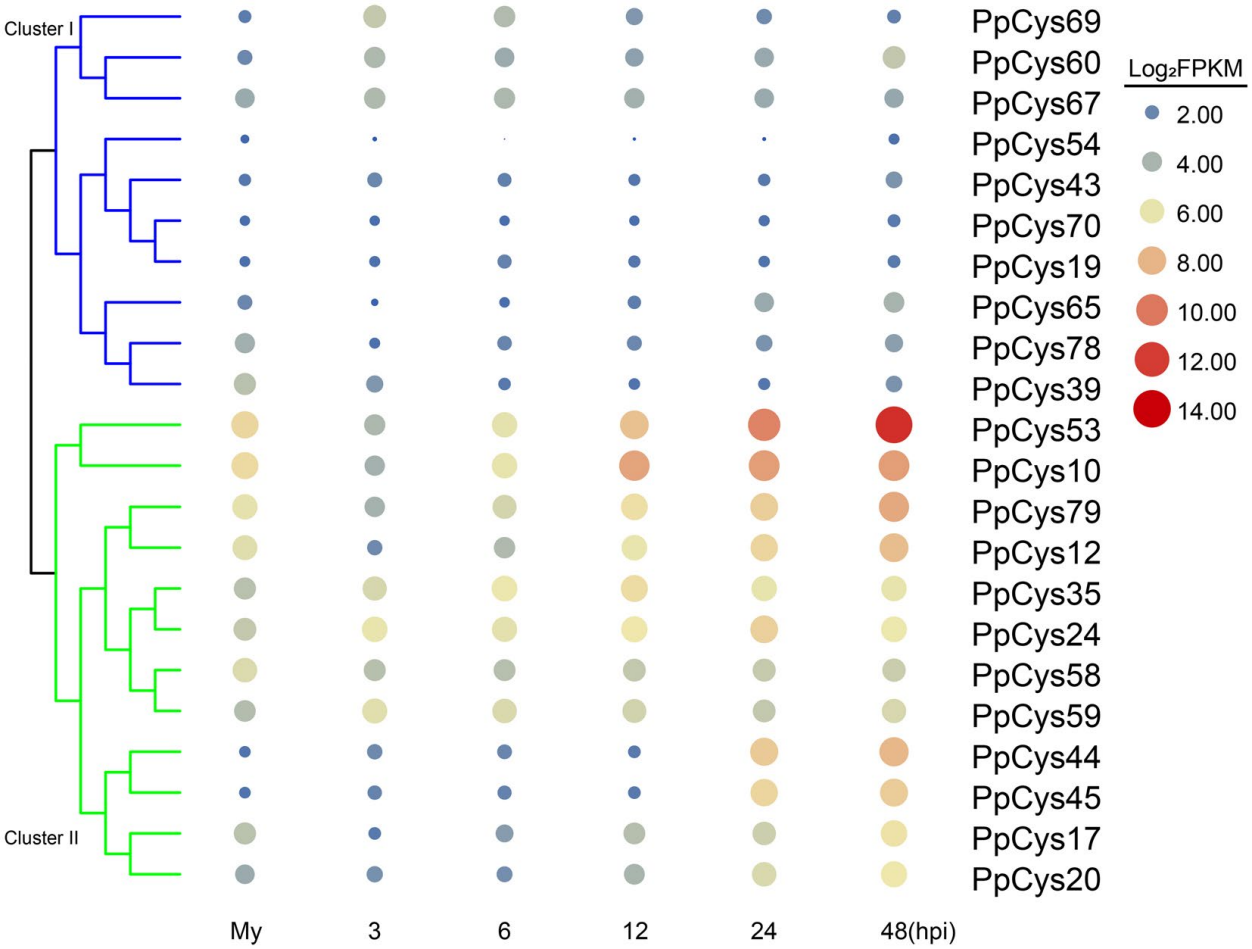
Analysis of the expression patterns of predicted secreted *Phytophthora parasitica* cysteine proteases based on RNA‐Seq data from *Nicotiana benthamiana* leaves infected with *P. parasitica*. Gene expression level is presented as the log_2_ transformation of the original fragments per kilobase of transcript per million mapped reads (FPKM) value, and values are shown for different stages of infection. My, *P. parasitica* mycelium grown in 5% carrot agar broth. The size and the colour of each filled circle indicate the gene expression level

We next analysed the expression patterns of the genes encoding secreted cysteine proteases during infection using RNA‐Seq data for *N. benthamiana* infected with *P. parasitica* Pp016 (Jia, 2017). Based on expression pattern, these genes were classified into two large clusters. The 12 genes in cluster II are highly induced during pathogen infection. Among these, *PpCys44* and *PpCys45* were barely detectable during the early stages of infection and highly induced at 24 and 48 hr post‐inoculation (hpi). Among the genes in cluster I, four (*PpCys54*, *PpCys43*, *PpCys70*, and *PpCys19*) had barely detectable transcripts at all stages, and two (*PpCys78* and *PpCys39*) were slightly down‐regulated, and four (*PpCys69*, *PpCys60*, *PpCys67*, and *PpCys65*) were slightly induced during infection (Figure 4).

### 2.3 Cysteine proteases induce cell death in various *Nicotiana* species

**Figure 5:**
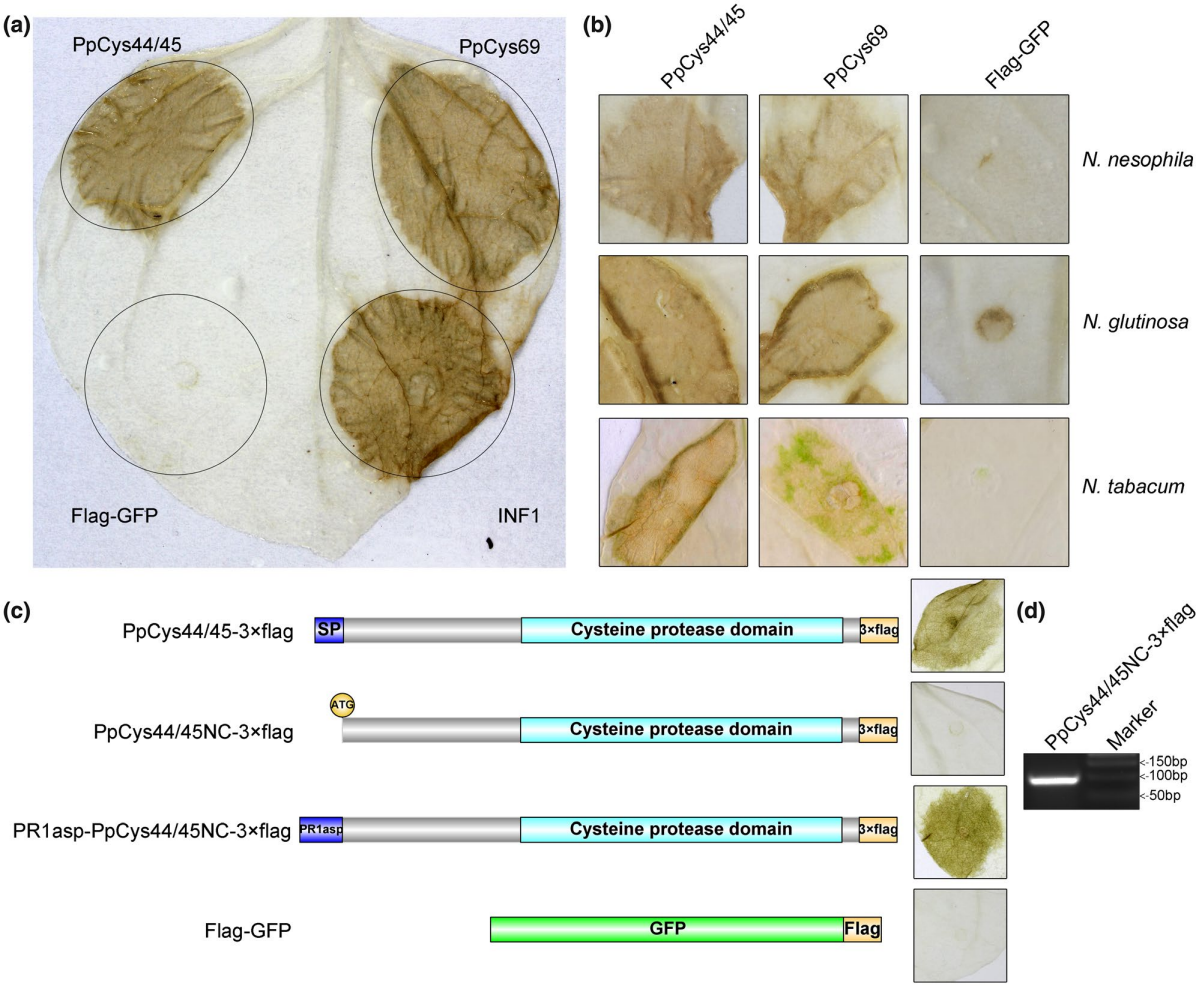
Analysis of cell death triggered by PpCys44/45 and PpCys69. (a) PpCys44/45 and PpCys69 induced cell death in *Nicotiana benthamiana* leaves. FLAG‐green fluorescent protein (GFP) was used as a negative control and INF1 was used as a positive control. (b) Cell death phenotype triggered by PpCys44/45 and PpCys69 in *Nicotiana tabacum*, *N. glutinosa*, and *N. nesophila* leaves. FLAG‐GFP was used as a negative control. (c) Truncated PpCys44/45 without a signal peptide sequence (PpCys44/45NC‐3 × FLAG) failed to trigger cell death, but the signal peptide of PR1a restored the ability of truncated PpCys44/45 to trigger cell death. (d) Reverse transcription‐PCR analysis confirmed the expression of *PpCys44/45NC‐3 × FLAG* in *N. benthamiana* leaves

To investigate the influence of secreted cysteine proteases on plant immunity, we performed *Agrobacterium tumefaciens*‐mediated transient expression of candidate cysteine proteases in *N. benthamiana* leaves to test whether they trigger cell death. PpCys44/45 (these two proteins share identical amino acid sequences, with similar nucleotide sequences except three single nucleotide polymorphism [SNPs] detected in their coding sequences and similar promoter sequences) and PpCys69 induced cell death in *N. benthamiana* leaves (Figure 5a), whereas no cell death occurred in the leaves expressing the other predicted secreted cysteine proteases (exclude PpCys54 and PpCy79, as these two genes were not amplified from cDNA successfully). The expression of all secreted cysteine proteases examined in *N. benthamiana* leaves were confirmed by reverse transcriptase‐polymerase chain reaction (RT‐PCR) analysis (Supporting Information S4). We also tested the ability of PpCys44/45 and PpCys69 to induce cell death in the leaves of other *Nicotiana* spp. and found that they also triggered cell death in *N. tabacum*, *N. glutinosa*, and *N. nesophila* leaves (Figure 5b). We also tested the influence of the secreted cysteine proteases on the pathogen‐associated molecular pattern (PAMP)‐triggered immunity (PTI) signalling pathway. The results showed that all secreted cysteine protease genes examined could not influence the hypersensitive response triggered by INF1 (Supporting information S5). As *P. parasitica* is a typical hemibiotrophic plant pathogen, and cell death in the late stage of infection may facilitate pathogen infection, we paid more attention to the function of PpCys44/45 (highly induced at 48 hpi).

Considering that virulence factors may function in different subcellular locations, we examined whether PpCys44/45 functions in the apoplastic space. *A. tumefaciens* cells carrying vectors harbouring the *PpCys44*/*45* gene with or without a signal peptide were infiltrated into *N. benthamiana* leaves. As expected, the protein harbouring native signal peptide sequence induced typical cell death in *N. benthamiana* leaves; however, the protein without a signal peptide (PpCys44/45NC‐3 × FLAG) did not induce cell death (Figure 5c). To confirm this result, we also replaced the native signal peptide sequence of PpCys44/45 with that of PR1a from *N. benthamiana*; the protein harbouring the PR1a signal peptide also triggered cell death (Figure 5c), confirming that PpCys44/45 functions in the apoplastic space. The expression of *PpCys44/45NC‐3 × FLAG* in *N. benthamiana* leaves was confirmed by RT‐PCR assay (Figure 5d).

### 2.4 The cysteine protease activity of PpCys44/45 is required for cell death activity

**Figure 6:**
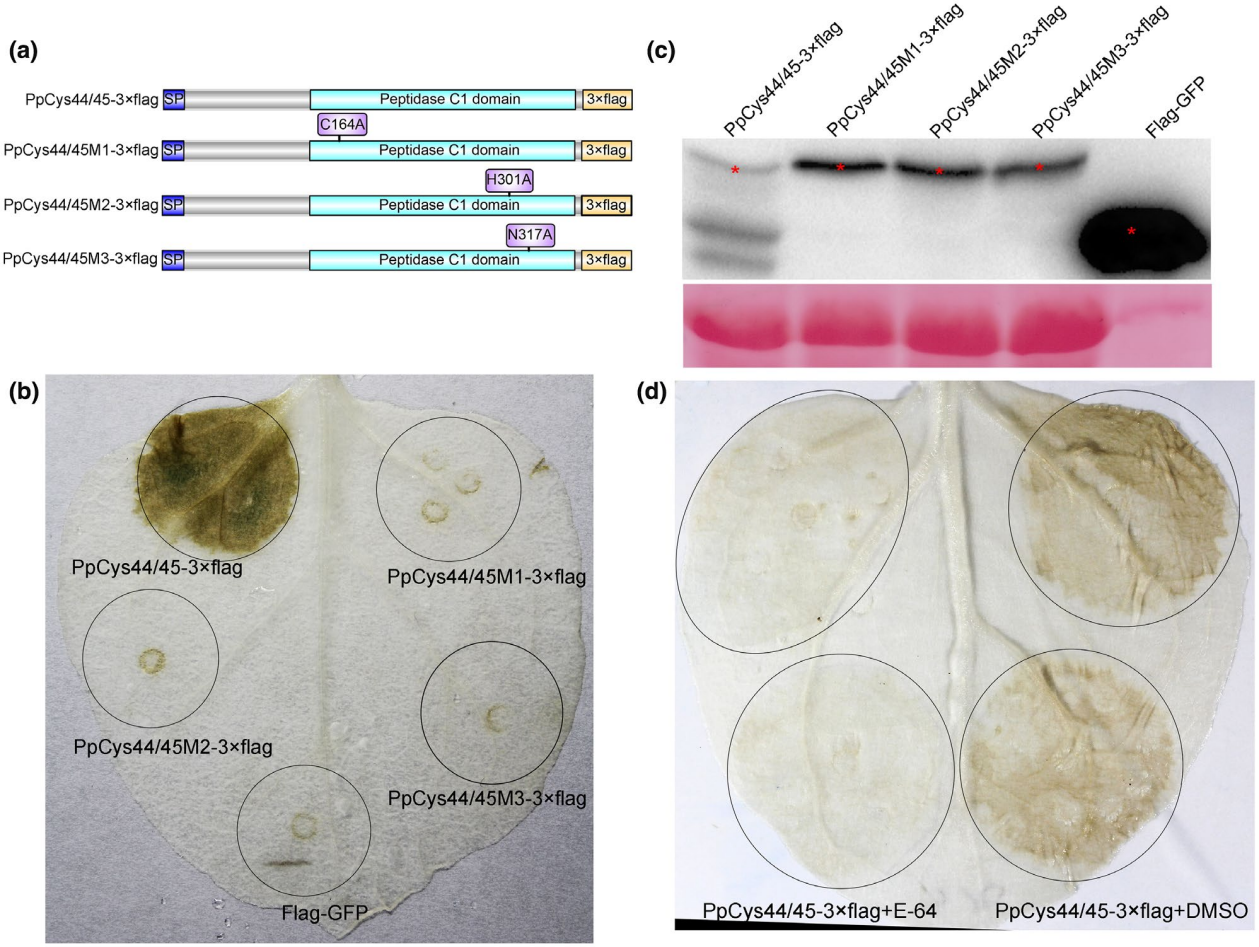
Cysteine protease activity is required for the induction of cell death by PpCys44/45. (a) Schematic diagrams of the PpCys44/45 catalytic site mutations. (b) The phenotypes induced by different PpCys44/45 mutants in *Nicotiana benthamiana* leaves. (c) Western blotting detection of different PpCys44/45 mutant proteins using anti‐FLAG antibody. Red asterisks indicate the expected protein bands. (d) E‐64 suppresses cell death triggered by PpCys44/45. Dimethyl sulfoxide (DMSO) was used as a negative control

PpCys44/45 contains a typical cysteine protease domain, and we tested whether this domain has catalytic activity. We made three mutant versions of PpCys44/45 by replacing the three predicted catalytic residues with alanine (Ala) (Figure 6a). These three active site mutants failed to trigger cell death in *N. benthamiana* leaves, indicating that the predicted catalytic residues are required for the ability of PpCys44/45 to induce cell death (Figure 6b). The total protein of the infiltrated leaves was extracted at 18 hr after infiltration before the cell death was apparent (about 2 days after infiltration) and western blotting was performed to determine whether these genes were translated properly. All these five proteins accumulated in leaves and were of the expected size (Figure 6c). The failed detection of wild‐type PpCys44/45 at 1.5 days post‐agroinfiltration probably resulted from the poor cell functionality caused by the cell death, not by the specific degradation of PpCys44/45, though the cell death was not visible.

We next tested whether a typical cysteine protease inhibitor, E‐64, could suppress the cell death triggered by PpCys44/45. We transiently expressed PpCys44/45 in *N. benthamiana* leaves, and 16 hr after infiltration the leaves were infiltrated with 400 μM E‐64 or 4% dimethyl sulfoxide (DMSO) (vol/vol). As expected, the leaves treated with DMSO showed a typical cell death phenotype, while the leaves treated with E‐64 did not show any visible cell death (Figure 6d). This result indicates that the induction of cell death by PpCys44/45 depends on its cysteine protease activity.

### 2.5 The cell death triggered by PpCys44/45 is dependent on *NPK1*

**Figure 7:**
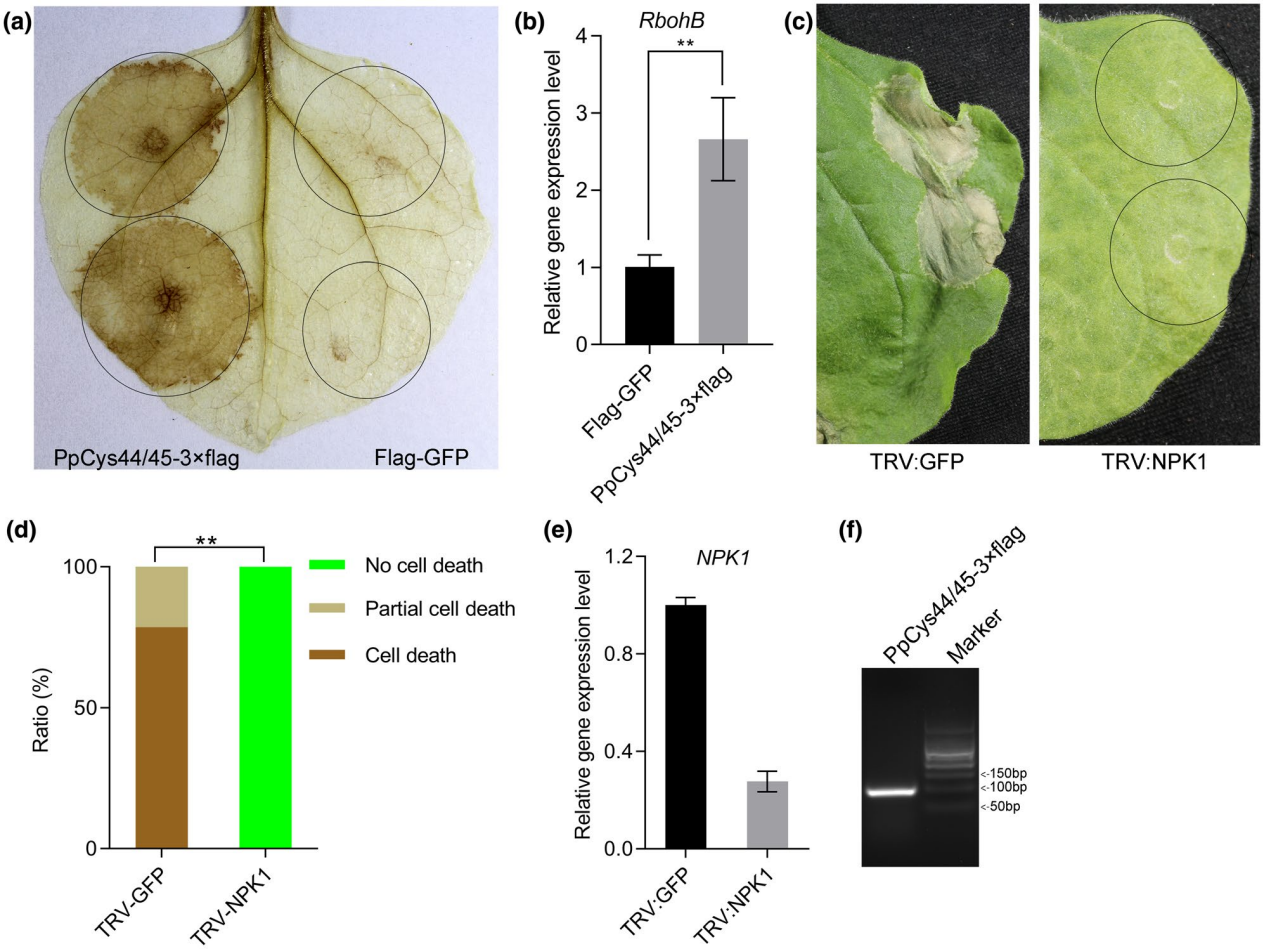
The cell death triggered by PpCys44/45 is dependent on *NPK1*. (a) 3,3′‐diaminobenzidine (DAB) staining of a leaf expressing PpCys44/45 and FLAG‐green fluorescent protein (GFP). (b) Quantitative reverse transcription PCR (RT‐qPCR) analysis of *RbohB* expression in *Nicotiana benthamiana* leaves expressing PpCys44/45. *N. benthamiana* leaves expressing FLAG‐GFP were used as a control. (c) The cell death phenotype triggered by PpCys44/45 in the leaves of plants with virus‐induced gene silencing (VIGS) of *GFP* and *NPK1*. (d) Quantification of cell death in different VIGS plants. The cell death phenotypes in *N. benthamiana* leaves were divided into three levels depending on the cell death area: no cell death, no visible cell death; partial cell death, cell death area/infiltration area <50%; cell death, cell death area/infiltration area >50%.The ratio showed the percentage of the leaves with different phenotypes. Statistical significance was determined by the Wilcoxon rank‐sum test (**p < .01). (e) RT‐qPCR analysis of the expression level of *NPK1* in the corresponding gene‐silenced plants. (f) Confirmation of the expression of *PpCys44/45‐3 × FLAG* in *NPK1‐*silenced *N. benthamiana* leaves by reverse transcription‐PCR analysis

Hypersensitive cell death is also associated with the reactive oxygen species (ROS) burst. To test whether PpCys44/45 could induce the ROS burst, we performed a 3,3′‐diaminobenzidine (DAB) staining assay on *N. benthamiana* leaves expressing PpCys44/45 or FLAG‐green fluorescent protein (GFP). The leaves expressing PpCys44/45 were stained brown at 36 hr after infiltration, which indicates that PpCys44/45 induced a strong ROS burst. The control leaves expressing FLAG‐GFP did not change colour (Figure 7a). We also tested whether PpCys44/45 induces the expression of the ROS marker genes *RbohA* and *RbohB*. The results showed that PpCys44/45 could significantly induce the expression of *RbohB*, but could not induce the expression of *RbohA* (Figure 7b, Supporting Information S6).

To investigate which pathway PpCys44/45 may elicit, we tested whether PpCys44/45 induces cell death in the plants with silenced expression of genes involved in plant immune signalling. We found that PpCys44/45 failed to trigger cell death in *NPK1*‐silenced plants (Figure 7c,d), which indicates that the cell death triggered by PpCys44/45 is dependent on *NPK1*. The expression level of *NPK1* in TRV:GFP and TRV:NPK1 plants was confirmed by quantitative reverse transcription PCR (RT‐qPCR) (Figure 7e) and the expression of *PpCys44/45* in *NPK1*‐silenced plants was confirmed by RT‐PCR (Figure 7f). The PpCys44/45‐triggered cell death is not compromised in *MEK1‐*, *MEK2‐*, *RAR1‐*, *SIPK‐*, *WRKY3‐*, *SGT1‐*, and *MYB1‐*silenced plants (Supporting Information S7).

### 2.6 *PpCys44* and *PpCys45* contribute to pathogen virulence during *P. parasitica–N. benthamiana* interaction

**Figure 8:**
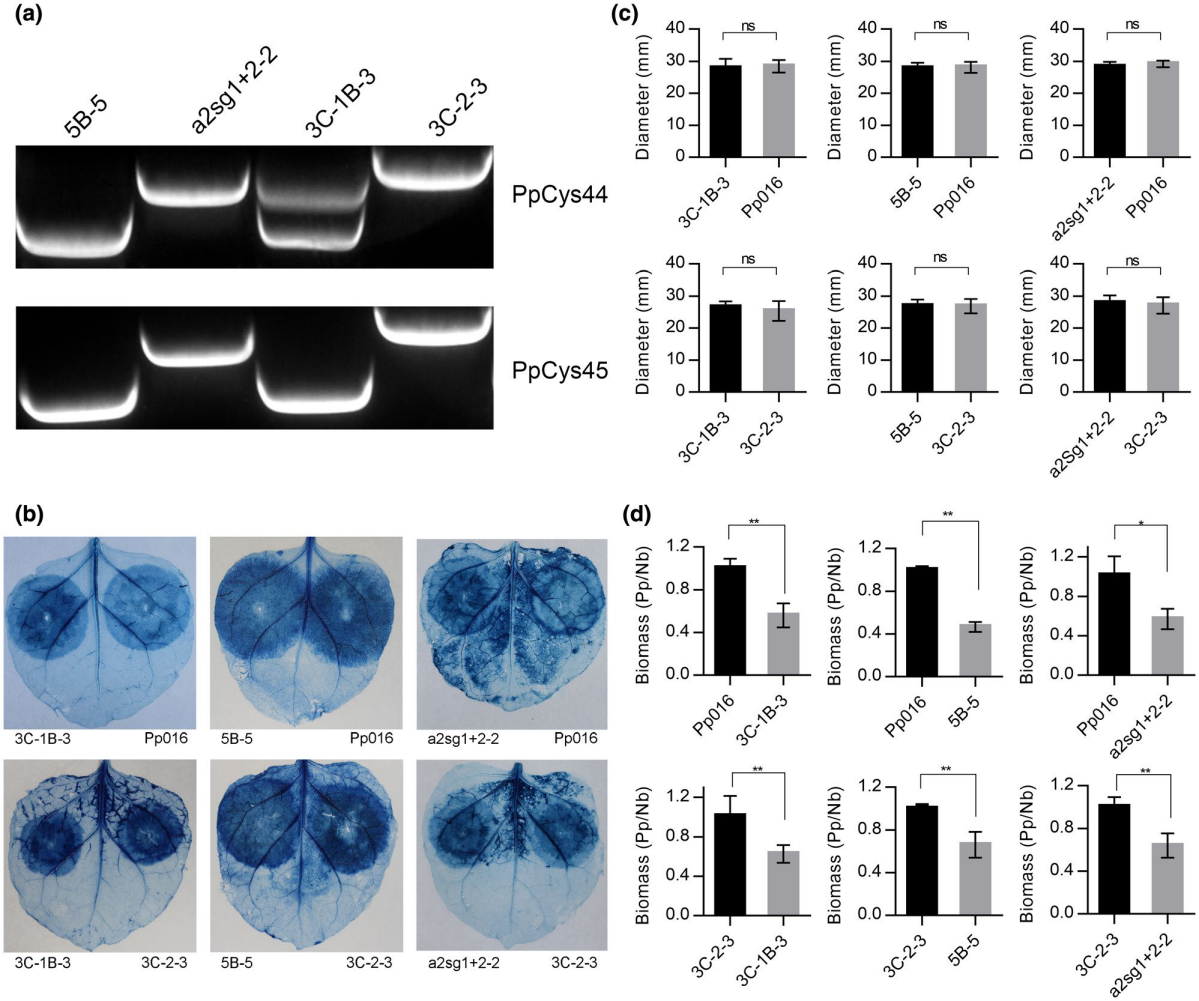
*PpCys44‐* and *PpCys45‐*deficient double mutants of *Phytophthora parasitica* showed reduced pathogenicity. (a) PCR detection of the mutations in *PpCys44* and *PpCys45* in four transformants. *PpCys44* and *PpCys45* were knocked out in 3C‐1B‐3, a2sg1+2‐2, and 5B‐5. 3C‐2‐3 is a non‐edited transformant recovered from the transformation experiment that was included as a control. (b) Trypan blue staining of *Nicotiana benthamiana* leaves infected with *P. parasitica*. There were no differences in lesion size between the true transformants and the wild‐type strain Pp016 or the control transformant 3C‐2‐3. (c) Statistical analysis of the diameters of lesions on *N. benthamiana* leaves infected with *P. parasitica*. Student's *t* test; ns, *p *> .05. (d) Biomass of *P. parasitica* on the infected *N. benthamiana* leaves determined using quantitative PCR. Pp/Nb, the level of *WS041* from *P. parasitica* (Zhang, 2012) relative to the level of *NbActin* from *N. benthamiana*. Student's *t* test; **p < .01; *p < .05

To investigate the function of *PpCys44* and *PpCys45* in pathogen virulence, we performed CRISPR/Cas9‐mediated genome modification to knock out *PpCys44* and *PpCys45*. As these genes share similar nucleotide sequences (only three SNPs) and have similar expression patterns, we knocked out these two genes simultaneously. Based on PCR analysis, we identified three knockout transformants (3C‐1B‐3, 5B‐5, and a2sg1+2‐2) (Figure 8a), which were obtained from three independent transformations. The sequences of *PpCys44* and *PpCys45* in the genome of transformants were confirmed by sequencing. Both the *PpCys44* and *PpCys45* gene sequences are partially deleted in the three transformants (Supporting Information S8), which led to mutations in the cysteine protease domain. To test the influence of mutation of *PpCys44* and *PpCys45* on pathogen virulence, we inoculated detached *N. benthamiana* leaves with wild‐type *P. parasitica*, knockout transformants and control (3C‐2‐3, a non‐edited transformant recovered from the transformation experiment). These three knockout transformants produced lesions similar to those caused by the wild‐type or control (Figure 8b,c). However, the biomass of *P. parasitica* on the leaves infected by knockout transformants was lower than that on leaves infected by the wild‐type or control (Figure 8d). This result indicates that PpCys44/45 contributes to the virulence of *P. parasitica*.

## 3 DISCUSSION

Cysteine proteases have been implicated as important virulence factors in bacteria (Hotson and Mudgett, 2004) and fungi (Ball *et al.*, 1991). Zuluaga *et al. *(2016) found that cathepsin B and papain‐like cysteine proteases were abundant during the *P. infestans–*tomato compatible interaction, peaking at 96 hr after inoculation, indicating that cysteine proteases may play a role in *Phytophthora–*plant interactions. In this study, we evaluated the expression patterns, protein sequence similarities among different strains, and cell‐death‐triggering abilities of the cysteine proteases secreted by *P. parasitica*. We then systematically investigated the functions of two cysteine proteases, *PpCys44* and *PpCys45*, which are highly expressed during the late stage of infection and trigger cell death in different *Nicotiana* species.

Apart from a few unconventionally secreted effectors (Liu *et al.*, 2014), effectors usually possess an N‐terminal signal peptide. In this study, we found that more than a quarter of *P. parasitica* cysteine protease proteins contained a predicted signal peptide, and the secretion of most of these proteins was verified using a yeast secretion system (Figure 2), indicating that these cysteine proteases may be secreted by the pathogen and play a role in *P. parasitica* pathogenesis. As reported previously, functionally important effectors are usually induced during infection (Wang *et al.*, 2011a). In this study, 16 out of 21 secreted cysteine proteases were induced during *P. parasitica* infection (Figure 4). A similar result was also reported in another *Phytophthora–*plant pathosystem, in which 12 cysteine proteases from *P. infestans* were found to accumulate at three different stages during infection of tomato plants (Zuluaga *et al.*, 2016), four of which are orthologs of the cysteine proteases from *P. parasitica* (*PpCys10*, *PpCys17*, *PpCys39*, and *PpCys53*). We found that all the secreted cysteine proteases are highly conserved among different *P. parasitica* strains (Figure 3) and most are conserved among three different *Phytophthora* species. Taken together, these characteristics of cysteine proteases indicate that they may contribute to the pathogenesis of *P. parasitica*.

As cell death can limit the development of some pathogens, it has long been considered as a resistance phenotype. However, in certain pathosystems, cell death is often uncoupled with resistance (Coll *et al.*, 2010). Some necrotrophic pathogens, such as *Cochliobolus victoriae*, secrete toxins to hijack the plant cell death mechanism to kill plant cells and feed on the cell remnants (Lorang *et al.*, 2012). *P. parasitica* is a typical hemibiotrophic plant pathogen and induces plant cell death during compatible interactions, which may promote infection. In this study, we identified two papain‐like cysteine proteases with identical protein sequences, PpCys44 and PpCys45, which induced cell death in the leaves of different *Nicotiana* spp. (Figure 5). PpCys44/45 contains a conserved cysteine protease C1 domain and the ability of PpCys44/45 to trigger cell death depends on its cysteine protease activity. There are two possible explanations for how PpCys44/45 induces cell death. First, as an active enzyme, the mature PpCys44/45 protein may cleave plant substrates, and the modified substrates may trigger a downstream cell death pathway. A similar mechanism was reported previously: AvrPphB cleaves PBS1 and modified PBS1 is recognized by an R protein, leading to hypersensitive cell death (Shao *et al.*, 2003). Second, as reported before, pathogen effectors may promote infection by mimicking the activity of plant proteins (Hotson *et al.*, 2003). Plant papain‐like cysteine proteases have been shown to participate in hypersensitive cell death (McLellan *et al.*, 2009). PpCys44/45 shares similar enzyme domains with plant proteins, and may mimic plant papain‐like proteins and take part in the cell death pathway.

In this research, we determined that the cell death triggered by PpCys44/45 is dependent on *NPK1* (Figure 7). *NPK1* is a tobacco MAP kinase kinase kinase (MAPKKK) homolog of human *MEKK1*, and the silencing of *NPK1* could interfere with the function of R genes *N*, *Bs2*, and *Rx* (Jin *et al.*, 2002) and the ROS burst elicited by INF1 (Asai *et al.*, 2008). The involvement of *NPK1* in PTI and R gene‐mediated plant immune signalling indicates that *PpCys44/45*‐triggered cell death is possibly a consequence of plant recognition. However, PpCys44/45‐triggered cell death is not dependent on the MAPK cascade genes *MEK1*, *MEK2*, and *SIPK*, the transcription factors *WRKY3* and *MYB1*, and the R gene function‐related genes *RAR1* and *SGT1*. To illustrate the exact immune pathway PpCys44/45 elicited, identification of the host target of PpCys44/45 is helpful and should be performed in the future.

The CRISPR/Cas9 system has been widely used for genome modification in different species. As for *Phytophthora* spp., CRISPR/Cas9‐mediated genome modification has been established in *P. sojae* (Fang and Tyler, 2016; Fang *et al.*, 2017) and *P. capsici* (Wang *et al*., 2018). In this study, we found that with minor modification the protocol reported by Fang *et al. *(2017) also works for *P. parasitica*. The successful modification of the *P. parasitica* genome using the CRISPR/Cas9 method will advance the functional analysis of *P. parasitica* effectors and other genes. In this study, we tried to knock out both *PpCys44* and *PpCys45* in the *P. parasitica* genome simultaneously, and the mutation efficiency was not very high. From about 400 transformants, we only obtained three independent homozygous knockout transformants. The results of PCR product sequencing confirmed that all alleles of *PpCys44* and *PpCys45* were edited at two different sites, which lead to partial deletion of these alleles. In the future, gene replacement and gene complementation may be performed to investigate the functions of candidate genes in *P. parasitica* using this CRISPR/Cas9 system.

We found that these successful transformants showed decreased virulence. This result indicates that *PpCys44* and *PpCys45* contribute to pathogenicity. Expression pattern analysis showed that *PpCys44* and *PpCys45* are very lowly expressed during the early stages of infection and then highly induced during the late stages of infection. Taken together, we speculate that these two genes do not participate in early stage lesion expansion but function at the late stages of infection, during which the pathogen kills the plant cell and feeds on cell remnants.

In summary, we show that most genes encoding the highly conserved secreted cysteine proteases from *P. parasitica* were up‐regulated during infection. Two of them, *PpCys44* and *PpCys45*, contribute to the pathogen virulence by inducing *NPK1‐*dependent cell death in different *Nicotiana* spp. leaves, which is dependent on its enzymatic activity.

## 4 EXPERIMENTAL PROCEDURES

### 4.1 Plant and *P. parasitica* cultivation and inoculation assays

*N. benthamiana* seeds were sown on soil in a matrix and cultured in a Phytotron with a 13 hr light:11 hr dark photoperiod for about 30–40 days. *P. parasitica* strains were routinely maintained on 5% carrot agar (CA) plates (Zhang *et al.*, 2019). Incubation conditions were as follows: 23 °C, darkness. The *P. parasitica* strain used in this study is Pp016, which was obtained in Australia (Wang *et al.*, 2011b).

For *P. parasitica* inoculation, *P. parasitica* strains were grown on a 5% CA plate for 3 days, then 5‐mm diameter mycelia plugs were taken around the colony edge. Detached *N. benthamiana* leaves were placed in a plastic tray covered with moist filter paper. Slight wounds were made by poking both sides of a vein with a toothpick. Mycelia plugs from transformants and the control were inoculated on opposite sides of one leaf and incubated at 23 °C for 48 hr. Ten leaves were used for one experiment and the experiment was performed three times.

### 4.2 Bioinformatic analysis of the cysteine protease family

Cysteine protease members in the publicly available *P. parasitica* INRA‐310 v. 2 genome (GenBank assembly accession: GCA_000247585.2) were identified by aligning the predicted proteins against NCBI's Conserved Domain Database (Marchler‐Bauer *et al.*, 2017). The orthologs of the secreted cysteine proteases in the sequenced oomycete genomes, *P. infestans* (GenBank assembly accession: GCA_000142945.1) and *P. sojae* (GenBank assembly accession: GCA_000149755.2), were identified using the BLASTP algorithm. Multiple sequence alignments were performed with ClustalW2 (Larkin *et al.*, 2007) using the default parameters. Phylogenetic dendrograms were generated in MEGA X (Kumar *et al.*, 2018) using the maximum‐likelihood method with 1,000 bootstrap replications. Signal peptide prediction was performed using SignalP v. 4.0 (Petersen *et al.*, 2011). The heatmap of protein sequence similarity was generated with TBtools (Chen *et al.*, 2018). The expression pattern of secreted cysteine proteases was analysed based on the RNA‐Seq data for *N. benthamiana* plants infected with *P. parasitica* Pp016 (Jia, 2017). Jia (2017) collected the infected leaves for RNA sequencing at 3, 6, 12, 24, and 48 hpi depending on the life cycle of *P. parasitica*. The fragments per kilobase of transcript per million mapped reads (FPKM) values of all the genes were calculated with TopHat and Cufflinks (Trapnell *et al.*, 2012) with the default parameter. The FPKM values of secreted cysteine protease genes were sorted out and the heatmap was generated with TBtools.

### 4.3 Cloning procedures and plasmid constructs

To generate 3 × FLAG‐tagged versions of the candidate proteins, we first constructed a vector carrying the 3 × FLAG sequence. The 3 × FLAG sequence was synthesized by annealing long primers and extending them with the high‐fidelity DNA polymerase FastPfu (TransGen Biotech) to form double‐stranded DNA (dsDNA). Then this short dsDNA fragment was inserted into the *Xho*I/*Xba*I restriction sites of pKannibal (Wesley *et al.*, 2001) using a ClonExpress II One Step Cloning Kit (Vazyme). The vector was digested with *Not*I restriction enzyme (NEB), and the smaller fragment was inserted into the binary vector pART27 (Gleave, 1992) at the *Not*I restriction site. The resulting plasmid was named P27:3 × FLAG. The open reading frame sequence of each gene was amplified from *P. parasitica* Pp016 cDNA and inserted into P27:3 × FLAG at the *Xba*I restriction site using a ClonExpress II One Step Cloning Kit (Vazyme).

To create the vectors used for signal peptide functional analysis, the signal peptide sequences of the secreted cysteine proteases were cloned from *P. parasitica* Pp016 genomic DNA and inserted into the *Eco*RI/*Xho*I restriction sites of pSUC2 (Jacobs *et al.*, 1997; Yin *et al.*, 2018) using a ClonExpress II One Step Cloning Kit (Vazyme).

To generate vectors for expressing the truncated version of PpCys44/45, the truncated fragment was amplified from vector P27:PpCys44/45‐3 × FLAG and inserted into P27:3 × FLAG at the *Xba*I/*Xho*I restriction sites using a ClonExpress II One Step Cloning Kit (Vazyme). The signal peptide sequence of *PR1a* was amplified from *N. benthamiana* cDNA and cloned in frame with the N‐terminus of truncated PpCys44/45. The three catalytic site mutants of PpCys44/45 were created by introducing a point mutation with traditional overlap PCR. All primers used in this study are listed in Supporting Information S9.

### 4.4 Functional evaluation of the signal peptide sequences of secreted proteins

Functional evaluation of the predicted signal peptide sequences was performed according to a previously published protocol (Yin *et al.*, 2018). Briefly, the sequence‐verified pSUC2 constructs were transformed into yeast YTK12 competent cells. The transformants that grew on a selective CMD–W (complete minimal media lacking tryptophan) agar plate were inoculated into 3 ml of liquid CMD–W broth and incubated at 30 °C with shaking for 36 hr. The cells were harvested by centrifugation and then washed with sterile distilled deionized (dd)H_2_O twice. The cells were resuspended with 750 μl of sterile ddH_2_O, and 250 μl of 10 mM NaOAc (pH 4.7) and 500 μl of 10% sucrose (wt/vol) were added into the tube. After incubation in a 37 °C water bath for 10 min, the tubes were spun at 12,000 × g for 1 min and 100 μl of supernatant was transferred into a new tube. After the addition of 900 μl of 0.1% TTC solution, the tubes were incubated at room temperature for 5 min. Those strains carrying a functional signal peptide can secrete invertase and reduce TTC to the insoluble red compound TPF, which leads to a change in the colour of the solution.

### 4.5 *Agrobacterium*‐mediated transient expression and virus‐induced gene silencing assay

*A. tumefaciens* GV3101 carrying different constructs were cultured in Luria Bertani (LB) liquid medium supplemented with the appropriate antibiotics. After incubation with shaking at 28 °C for about 36 hr, the bacterial cells were harvested, resuspended in infiltration buffer (Huang *et al.*, 2019), and adjusted to the appropriate concentration. For transient expression, the bacterial concentration was adjusted to OD_600_ = 0.4. The biggest leaves of 30‐ to 40‐day‐old *N. benthamiana* seedlings were infiltrated with a needleless syringe. An *A. tumefaciens* strain harbouring a plasmid expressing FLAG‐GFP was used as a control. To test whether the secreted cysteine protease genes influence the cell death triggered by INF1, the *A. tumefaciens* strains carrying vectors with a construct of secreted cysteine protease gene or GFP were infiltrated into the *N. benthamiana* leaves (OD_600_ = 0.4). Twenty‐four hours later, the *A. tumefaciens* strains carrying *INF1* (OD_600_ = 0.05) were infiltrated into the same area of the leaves. The cell death phenotype was photographed at 5 days after infiltration. Statistical significance was determined by the Wilcoxon rank‐sum test. For the virus‐induced gene silencing (VIGS) assay, *A. tumefaciens* strains harbouring different pTRV2 constructs were coinfiltrated in equal ratios with those harbouring pTRV1 vectors with a final OD_600_ of 0.3. pTRV2:GFP was used as a control and pTRV2:PDS (phytoene desaturase) (Liu *et al.*, 2002a) was used to monitor the silencing process. The lowest two leaves of four‐leaf stage seedlings were infiltrated as previously described (Ratcliff *et al.*, 2001; Liu *et al.*, 2002b). Eight plant genes involved in the plant immune signalling (Huang *et al.*, 2019), *SGT1*, *NPK1* (*Nicotiana Protein Kinase 1*), *MEK1*, *MEK2*, *SIPK*, *MYB1*, *RAR1*, and *WRKY3* were silenced to test which pathway PpCy44/45 may elicit. The pTRV2 constructs for silencing these genes and primers used for the gene expression level detection are the same as described previously (Huang *et al.*, 2019).

### 4.6 DAB staining

DAB staining was performed according to a previously published protocol (Daudi and O'Brien, 2012) with minor modifications. Briefly, *N. benthamiana* leaves were stained with DAB solution (1 mg/ml) at 1.5 days after infiltration before the cell death phenotype was apparent. The leaves were incubated in DAB solution with shaking (100 rpm) for 4 hr. Then the DAB staining solution was replaced with bleaching buffer (ethanol:acetic acid:glycerol, 3:1:1 vol/vol/vol) and the leaves were extensively cleared by incubating them in a boiling water bath for about 15 min.

### 4.7 Single‐guide RNA design and CRISPR/Cas9 plasmid preparation

The protocols for single‐guide RNA (sgRNA) design and CRISPR/Cas9 plasmid preparation were modified from previously published protocols (Fang *et al.*, 2017). The sgRNA for PpCys44/45 was designed with *EuPaGDT* (http://grna.ctegd.uga.edu/), and sgRNAs with a total score larger than 0.5 were chosen for RNA secondary structure prediction and off‐target analysis. Three candidate sgRNAs (sg1, sg2, and sg3) without strong RNA secondary structure and off‐target sites were chosen for primer synthesis. Synthesized primer pairs were annealed to each other and extended using the high‐fidelity DNA polymerase FastPfu (TransGen Biotech) to make the dsDNA fragment. The dsDNA fragment was inserted into the *Nhe*I/*Bsa*I restriction sites of pYF515 (Fang *et al.*, 2017) using a ClonExpress II One Step Cloning Kit (Vazyme). The sequence‐verified plasmid was purified from *Escherichia coli* DH5α cells with a Maxi Plasmid Kit (Omega Bio‐Tek). The concentration of prepared plasmid was measured using a NanoDrop spectrophotometer, and the DNA solution was diluted to c.5 μg/μl with ddH_2_O.

### 4.8 Genome editing of *P. parasitica* using CRISPR/Cas9

Genome editing of *P. parasitica* using CRISPR/Cas9 was performed following a previously published protocol for *P. sojae* (Fang *et al.*, 2017) with minor modifications. *P. parasitica* strain Pp016 was inoculated onto a 5% CA plate and incubated in the dark at 23 °C for 3 days. Ten fresh *P. parasitica* discs taken from the colony edge were transferred into a 250 ml flask containing 50 ml of nutrient pea broth and incubated at 23 °C in the dark without shaking for 2 days. Four flasks of *P. parasitica* culture were sufficient for four transformations. The entire culture was poured into a sterilized 1‐L beaker covered with a layer of Miracloth and three layers of cheesecloth. The mycelial culture was washed with sterile ddH_2_O, then washed in 0.8 M mannitol for 10 min. The hyphal mats were poured into another beaker covered with Miracloth and cheesecloth to remove the mannitol. Then the hyphae were uniformly dispersed in 40 ml of fresh enzyme solution (0.4 M mannitol, 20 mM KCl, 20 mM MES [pH 5.7], 10 mM CaCl_2_, 2% [wt/vol] Lysing Enzyme from *Trichoderma harzianum* [cat. no. L1412, Sigma] and 2% [wt/vol] cellulose from *Trichoderma viride* [cat. no. 219466, Sigma]) and digested for about 1.5 hr with gentle shaking (40 rpm). The digestion products were filtered through a 70 μm nylon mesh to remove the hyphal debris and collected by centrifugation for 5 min at 500 × g. The protoplasts were then washed twice with W5 solution, followed by incubation on ice for 30 min. The protoplasts were collected again by centrifuging as described above, resuspended in 4 ml of MMG solution (4 mM MES, 0.4 M mannitol, 15 mM MgCl_2_, pH 5.7), then placed at room temperature for 10 min. The CRISPR/Cas9 plasmid (three different combinations: 60 μg of sg1 and sg2 [1:1] or 60 μg of sg1 and sg3 [1:1] or 90 μg of sg1, sg2, and sg3 [1:1:1]) and 1 ml of protoplasts were gently added into a 50 ml tube. The mixture was placed on ice for 20 min. The protoplasts were mixed gently with 1.74 ml of 40% polyethylene glycol solution until well mixed and placed on ice for 20 min. Then, 10 ml of pea broth/0.5 M mannitol was added to each tube in two batches and the tube was placed on ice for 2 min after each addition. The solution was poured into a deep 90‐mm Petri dish containing 10 ml of pea broth/0.5 M mannitol supplemented with 100 μg/ml ampicillin and 10 μg/ml rifampicin and mixed well. The transformed protoplasts were incubated at 23 °C without shaking for 22 hr. The regenerated protoplasts were collected by centrifuging at 2,000 × g and resuspended in 4 ml ddH_2_O. Then the resuspended protoplasts were diluted with 150 ml of c.40 °C liquid 5% CA medium containing 0.8% agar supplemented with 200 μg/ml ampicillin, 20 μg/ml rifampicin, 20 μg/ml nystatin, and 10 μg/ml G418 and poured into nine 90‐mm Petri dishes. Then the Petri dishes were kept at 23 °C for 5 days or until mycelial growth appeared. The candidate transformants were transferred to a new 5% CA plate containing 200 μg/ml ampicillin, 20 μg/ml rifampicin, 20 μg/ml nystatin, and 10 μg/ml G418. The nutrient pea broth and pea broth/0.5 M mannitol were prepared according to Fang *et al. *(2017).

### 4.9 DNA extraction and screening for genome‐edited transformants

After incubation in the dark at 23 °C for 3 days, *P. parasitica* discs taken from the edges of the colony of candidate transformants were inoculated into a 12‐well plate containing 5 ml of 5% CA broth. Mycelia were harvested after incubation at 23 °C in the dark for 4 days and ground to fine powder in liquid nitrogen. The mycelial powder was resuspended with 500 μl of DNA extraction buffer (200 mM Tris‐HCl [pH 8.0], 25 mM EDTA [pH 8.0], 200 mM NaCl, 2% SDS [wt/vol], and 0.1 mg/ml RNase A) (Fang *et al.*, 2017) and extracted with 250 μl Tris‐saturated phenol (pH 8.0) and 250 μl chloroform. The DNA was precipitated with 800 μl of a mixed solution of 7.5 M NH_4_OAC and isopropanol (1:3 vol/vol) and washed with 70% ethanol. The air‐dried DNA pellet was dissolved with 30 μl ddH_2_O. Primers PpCys44‐F/PpCys44‐R and PpCys45‐F/PpCys45‐R were used to amplify the full‐length coding sequences of *PpCys44* and *PpCys45*, respectively, and the PCR products were assessed with agarose electrophoresis. We could distinguish the wild‐type and heterozygous and homozygous transformants based on the PCR product size. The PCR products from candidate genome‐edited strains were reconfirmed by sequencing.

## AUTHOR CONTRIBUTIONS

W.S., Y.M., and Q.Z. designed the experiments. Q.Z., W.L., J.Y., Y.M., and J.X. performed the experiments. Q.Z., W.L., Y.M., and W.S. analysed the data. Q.Z., Y.M., and W.S. wrote the manuscript.
