# Supplementary material for: Two Phytophthora parasitica cysteine protease genes, PpCys44 and PpCys45, trigger cell death in various Nicotiana spp. and act as virulence factors
Source: Mol Plant Pathol. 2020 Feb 19;21(4):541–54. doi: 10.1111/mpp.12915 (PMC7060141; doi:10.1111/mpp.12915)
Supplement: Supplementary file 1 [file MPP-21-541-s001.docx]

>PpCys01_PPTG_00866

MSRSVTKKLLLLLNECWMLDQKSISQASEKMVEGVNGNNTTILLPPMKTPVPKAPKKGMRIPPTTLRLITAASSGRIFLPLNINGTHWTYIVVDGSTQTVCCYDSTDKRANHNLLAQLADEIVKKSLTKAFSVTVVHGPIQKDGFNCGVFICLYFWRRFWKGAGSDYTEEGLLRRRWDILRIIMEFSDESKDEEKVTE

>PpCys02_PPTG_00906

MVDYGFKFKTPTARTISSLPAKLFGPRSNVMIVDPGLIGSIENNDSLINTSVSTEDFAGATKEKILIPVCCSKKRWCCIMLDLETTDICIYDPMGSSYIIRVRALAEKLATCLPDYTPRKYRVQPYQSDLGVQVDSYNCGVYVLVAFELFAGAAGLP

>PpCys03_PPTG_00999

MHVRASMQKKKIIPLNYIHSRWLLQAETNRPEEDEADEDISCSSLRVQTSHMRSVEHAVLNGTTKWKTAMDIAGGVVEAMSSHGTRTFLRMTEVFREFGECVREGVVPTLMKPQTTSVESLLRAPSGDFYVQHDTELADSDSSSVPPTHEATNTAQSNTTVDGFNVESDCGDSINGESEQLPDECDRLLAETNRIITRVRDMVSSDSDNDDTELFELVQPGNKNTPTSRTLKSAKIQETNQSVVITKQSGTATNQLKTSTIQSVSCAVQKPSNTFDLVDKAAASELSQSVNDTTSLAEKSVPLVDTTNAEPNHSVDDGNQSVRSSNQSVRAGREFADQPTEATCQLVLRKRRSKERGDSSPGDSFSHEGISGEFRVSTSVKSKGRPKIRRKQAREAKKLRMDESIAEAKALVQGTLVPEKDLALVRKTLACSFNVEDALPVLHSIAKVDAPTWKATSIVQLARKQKVHKKVTIVFPKNYVTKCIAGINMYRKSFPSDDEAGRMGVSIKKLGTFTEKDLITMRDWHDESPKFDAFCDLAAWIRVSRFARITLPSPLNNCVTVDLQACAKRLETVNLKTTMDTRFGQVGIEEMAFFRRSEWLDDSCMKLVMSHLMDQDQDENKRSCIGAVNPLYARVHDEAMKLQVIGSSPLRSSNRMILVPVYLDGHWAGVVFDNAKSRAVVFDPMQTNKYYNQACTVIKKYFGNYSSNLKLVRQHAPRQKDTNSCGPLVLLFFECMVRGMAVPNVAREQLAYLRFRYLFLTSKGVFCRSPDTATSEE

>PpCys04_PPTG_01258

MMATRPINEEHKHPKSSKDEDVGTASDNETKEDTEALKSTNEDKKIVMPLSTAANGSKDETSITAKSREEEIAELEEILRAQHLSRTSKADLLFYNRLAEREANTILEEVLLEDIYDIAEDVFDEEEEKSRNKELPPELLEIVENALHEGPMEEVLINKYNVDITRRHLQCMLPGTWLNDEVINFYFQMLSDRDEALVKAGVLPKRSHFFNSFFYTKVSENGYNFINVRRWTRKIDLFAMDKIFMPVNVGNMHWCMAVIHMTEKRIQYYDSMHGSGAACLKVLLRYLHDESEHKKKQKFNDEGWELVTTTPDTPQQNNGSDCGVFSCMFADYLSLNKPLSFVQKDIPFHRHRMVLHVSRGYIPLEEEGL

>PpCys05_PPTG_01687

MGDRRPRRFGARHHTTRVSAPSGPHGGGPGGSHYANTLEFETVASERSVPRNHQHRANWEDTHSQFDFSQDNQSRGQLEGPHAPDKYKSSRPKASGQSISAPGSSFANSASSTSTPGVADGVLSKGFIPPKSQGDFLDNLTKMNTRANRGSGSILKAMAYSTSSSSLEITSAPPKEDISRPHTGSSGWVNTRMKQLPAKPRILLTASEKRMKLSNNTRKTPSPVVSHYSLRDRNKTTLFGISPTSTAPFSIRNSRVSRLGKRKMSSEGSASKPIALDSDSEPDVQSDRDIVDEVLDDTRRTTPLKKQDVKAAVIDLDDMALRAMARLNNCDVMIGLFQCIVDMFFQGDSMCMRNIRGKNETWPFEENYLFNLDKLHDIRYYCVSKEVDVSVEIDVDEPDRMEQLLDEASFMAFKLPFASEADQTAMKDFYDPTGTDVSKGYMVLRPLEDTTGGDLSQIQEHLRSFGDVQAINDKKQAKEHLEALLRDPFSYKASRQSQRSATEADAESSSESEEDDEEGSITVLTYPLPPCTSDIVTIIRRDVSRLKPRRYLNDSIIDYYFKRMMLDSYRDNKTVQEKVLFLNSHFYTRLRGGKGATALERMEAGYKNVSTWLARSNFFDRSIIFIPINKEYGAVDELWQSLWC

>PpCys06_PPTG_02273

MASKDETLGQTKQRHKLELRTLQNDVKAFQKKAKKDRMSKKDIEAQVLAMENAVKERHDAELKAFKTEEDEAETPAAAPAEPEAAVPSKQAKAQAKAQRKREAKKQQERERRERIEEANKNTVSERQIEADMILAQLARQGLKIKDIPSDGHCMYHAVADQMKQMNMPIADEVAGFQYLRKLTSEYMLAHPNDFLPFIAVDESSANPEDAFVTYCDRLANTADWGGQLELRALACALQTPIEVFSAHGDVLVMGDEFVNDSAPKLQLTYHLHYYTLGEHFNSVTPV

>PpCys07_PPTG_02444

MVCERLVASPRERPDDPMDGRSHTASDDSDLEEEMMRVNVFDYEWRRQMHVGDQVDAQNVFGNWCPARVLQIADGEVLLQFLNMHESWHQWLQMDSGRLAQSGIKARNDSAPIRQAQQIEVRPGGGNSSLWKEALAVRAGGGSVLVRYPGRDQKFDEWIQFTPAKVAPAGEHLQLRGRGSTLEQRKLAVDAPNATHRRVIQAQNPRFTHYRDSLQATLGLTIHDIEGDGNCLFRSVSHQVYGDDSHHALVRAACMDYMESEKEYFEPYVVGDMAAFMRYLRYKRRDGVWGDDPELQALCELYDRPAEVFAYDPQQGFRKLRCFHENSALARTRPPIRLSYYGGGHYDSLVGPDHQANLIRERPGQWEQRHIGYSHRINSRETRNDVSGVEQEVQAQSDRERTEVEQLEQVLVQSRNEFDAMDKSLEETLKLSLAEYGEASPAQAHREREEIEEATRESEMAAIQAELLAKAKAESEEEQMKSAIQASLGDHAVDFDAQISAAIQASLGDVGASVPAASGAADYDEQLRRAMELSAQDYAPPQDFGLYGNESTNTDEMDELQRAIQASLERS

>PpCys08_PPTG_02860

MVRAKHTANILRSLQIQATTSSSSSSDAESDGENGSDSNESSGTNEARFEGIPNAQTTDDIEEIDSVPDGNTGTHSDAKNSNVSGEVIEEKLPQASADSDQADSEDEERSEVASSGSRKRDHAECEDELYYAAPLRKYHRSWKDLEKRLKSYQEETHTVLVISETMNVRLRNQKISKMKIHAGKPQSELPLVPEELDPYQRVYICTHGWKERVRSKGQRPRQNMKGVGCPMRFRAQFVERDEGKWRIEIKQAFYGHNHVLSEEVYRVYPSVRQVPVDSPIISDVELMVASGSKASKIYEYIRERTPHYIQLKDVHNLVARIRNSGGRLSDEDFVAELLVSFELESPGNVAAIDEDGSGHTAVVTISSQHMRKLYKRFPEILLVNCTHKTNRCVSFLIMVKQVLARFRSLANDWKLTKVIMVDKDLTEIGVLRSMFPDARILLCQFHVLKWLRGAVRDDSKYDTFPSAILKQMDHCVSNMVYSKSDDEFQLHAAEFKYLACRDGRHTLWTYFDRNWVACKEMWVTQHRMELPHFRNNTNNRLESFFGKLKADLDSSMSMRECLEATIRYQRRKEDEYATRVVMPGTRRDLTYDDEMNQLLGMTSEWVAEVFEPEYKFALDPNVAKFYRIEDEDLYVNLYRDGRKHRVDKYNWMCTCEFSSTMKLPCRHGMIYRKHVAHMLTIPYSSIPASFTMFPALGALDEEIAAVDVPLRISAPCADVKRMKRMSDRDKYRAAQSAFSRINTELTDLPDDKFRAALDHLEKWGARLRRGDVRMGTQEAPDEVKPSSQVAKPVAADKPDEEKDAKRHGGICDQEERDENDQEEKEGNEEKVENVHGGDQEEKNENSISKVFSKQSQQKKVKVKPFNPRTRVGRPRKNRALEASQRKQSRQEFNQGCKLRGALRGDDVVEVADFIRSCEPPLSDLASFLNTFEERFSKLKPKQISVVWRVPEPTIAPYRLPEVNATAECWVLTVDGIGEFTHQQLLAMQYVWTLVHASEAGMSCYSWLMNIADDQISHEDTAGLAKRIWDAWPQEILPGFGIGFDITWVHLYSARSGIWYNDNLISAYAKTMESKYGNNTTIFLPAMKIPLPKTPKKGMRLPTTTLSEVSAASEGVIFMPLNINNNHWTCIVVDGPKQTVYCYDSTNKRANHNLLAELADELVKKSLPQGYSITPIHSPIQKDGYNCGLFICLYFWRRFWKEAGSDYTETGLVRRRWDVMHLIVEFSDGSKEKETVAA

>PpCys09_PPTG_03223

MSRDAVDAVPRAADDAPAQEDMRDESPLGGGSVIANEQVIAESGGEQDRRSAGDDAKQAVEPGGSLISQTEDPGGRNMNVKASEHMDVEDERNTEAARSSEMDGDGEDAGSQNTSDAASVTVDPAVKYHANWEAWQNYFVDYCRRTLQVIPVKETMSCAERNKRLKKTKRGVDEDQLVPTEFDPYQRTYICTHGWKKRKSRSEGSRPRQHVRLTRCPFRFVVQWNLARGELQVKNGVWLHNHQVSPAAFATYPSSRGVFHPLVGARVQGMLEVGAKRSRIYDYLLEHDENVMQADVDNLVRDYSSSMTNVDDNEETARELALFAAVDPENLLSVADTDCGETGVISIASAHMRLVFARFSEVLLLIAAIRRIGAYNYQLLTFMGMNEFGEGAVVQQSLIEANGDWHMERAIDHFKRFHPTRIDLLQVIVVDKDLNEIRVLEANFPAARILLRHFHVIKYLKEMRSKPEFGKISSDDASQVDAAVHKMVYASSEHKYKEAHESLKGFCERCGIDRFFKYFEKNWHSCTDRWVYYLRATLPHFNNHTNNRLESYFGKLKEGIDSLMSMANCIKALVAFDRRKQNDYEYRLTRIGRFSNSNYDEEMSTVLRFTTHYAARQIERQYILGLENASMYNFEKDPEELSVVKIGGIFKTHALRTDDWKCNCEFAASMGLPCRHAIAYRKYTNVRTHSERYTEAVRATHLIASEIADIEDEAEFESMLQFVMSQWRNVRQKKIAEDIPEGDLKEALKMEDRHAFSDADLKWEFEISSSDDEECCTGDAANSDANTEKNPPASSVSIRLNPKARKVGAPKKAKKKIVAGERADRKWYEAAKEGRNKSGEVTLLAVVNSLDHVQPGLREVQRRLSGIIVKYGDAESKKPKLHMMKNPVLIQDPFYLLPTKLLEACIKILPVSNSKEDAITIDTSQTSQPTEEKTGKLVETIVIKDQVKEDGVDTVLLPLNFDNFHWCCVVVKANAKRIYYYDPLNHASYKNACNAVGTHLKILGLLDYDVIAMNNPIQFDEHSCGVFVGWMFICQAISHDTMEDDDKEEKTPAPRNTGDDDAGDEVQHTQRAARIEDAEDEVSPTQRAAGVDDAKSEVLPTQPAQ

>PpCys10_PPTG_03239

MKVFTSLLLAATAAFAPASALTTDLPSSLSASEQQTWEAFVDYALDYEKSYRYDTYDQDLVQQRFRAFATNLERIQMHNAAYERGEHSFTLGLNELADLTDAEYKQLLSYRASVSKASRASETFVKPDNIEDLPATWDWREHNTVTPVKNQGQCGSCWAFSAVAAMECAYALSTGTLESFSEQELVDCTLNGIDTCNHGGEMSEGYEEIINNHKGKIDREEDYEYTAESKGVCNAKDDKAIGHFTSYANVTSGDEAALQAAIATKGVQAVAIDASSFTFQLYRHGVYSWPLCGNAPDALDHGVAAAGYGVYKKKDYWLVKNSWGDSWGMKGYIMMSRNKNNQCGIATDATYPIMTKEEEIVVDRPIVLETTEVASIM

>PpCys11_PPTG_03579

MFQWYGRSRLSTQGLSKVAKPFVAKVFKRDDAAISRDRTEKEKFRRAQQAFGRISGELARLGDEAFDAAMDQFEGWWHNLRQGQISMTPCHEQTESRDNDDSNDGGNACGGGNAEDTSSVAQAISGGGSSPSSDESEDLALTQQTAVSTQLTQQKGQRAKAKADLKEYNQGMKLRGLLRDRDVCGVVSALKEIQPGILEMGAFLVTFQVLGKATPKLSMAWRLRTDIVADNVRYRLPEETVNRAFELLSESRTGQKDEIQLDSDGEGVGDENGYVLVAEKVGTYSREQVEQMKWMWNMQDTCRRGVLLCTWLNNEVKTLVDEKVAVSVAFDNFLKSWPYLVIPGFGFDITYADLFCIRGSTWLNDATMRAFCVFLKTYKNNATVMIPPVKKQTQFVLMPINLSGVHWVCLVVDGTAKKIQVYDSAGSAMYLKRLKNIASEIASTLPDMYEEIVFDGPLQTDGDSCGVFACLQLWKSVSSKAPTDVSESGIIKLRWKILQAILKVKRRS

>PpCys12_PPTG_04091

MALWSFLLSAALSLSIVSASARGPIIGVFAHPISQHGEYIAASYVKWVESAGGRVVPIPYNAPKPYLEQLLPQLNGLLFPGGAAIVNDRAERLFQLALELNDKGVHFPVWATCLGFEWLVQLTTKDMDSLNKGLDSMNITLPLNFTDAAPTSRLFSQTSPELYSWLKDKPITMNNHEQGITPERFNKYSSLTDFYTVLATNVDRQGVEFISAFEAKEYPVYAVQFHPEKNSFEYGEYQDGTPYEVIDHSREGVASGQFFANFFIEEARKNDLRFKNPKEERKALIYNYQTSTITDPGFVESYIFKHDFKMDFWSVQM

>PpCys13_PPTG_04375

MPMNFKVTFANGTSMKLMGLPDDITLDGLRAHLFEKTSILPQEQHVMTGYPPKMIEGSGESTLTSLGVRTGSVLVLKEAPENSAAQSKTKSVFMRRVMPADNSCLFHSIGYALGKGREGNGPVMRQLIKDTILADPEKYSEVFLGRPVYEYCAWIMDDKSWGGEIELSILSTYYKVEMVVFDVTSMSRLCYGEDQGFTQRLFLLYDGIHYDLVVEAPSATASESQDVTLFAINDFSKVGFGR

>PpCys14_PPTG_04444

MLPCQLLMYVCRYGHGFEELPIMTIPSRWSMAEATKLFPQLEKSASSIDTVTSTIKLRNSARWRAMSAATLSSTSNVNHTKIVLGSKARVAYVLLRRRESGNVVVLSSEEKYTMAKSVFEPLLNHLQSLSGTGFYKALEKWEDFVADAVKNTDKASDTSDTDEDNYDVDITDYYDAVQLVEEMEKMQYEEEKEEADICPPTQPSTVVTLTQSATLPEQATLTETAANSANAPTTSPLPATLSATATLPASVEPGAAQGVHVTAAEMPLSAFAPATYSEFEEASVSQISLGDINMSESSQRIIEATNDTVLGVEADSTPAQRGSNKHVPVTELVAGSQKREIELNLPRPHQGKPRKKLRQKWSAVAAKPRYVIVEYPRGLTVTVAEVVKWALYTPSLKTVADILERFPCIMNEQTLRGRARRVKEVTQQTASSYCYSYVIPKDLVVSLQAAINDFRASKKDECKDIQKAPEVVDLTSTPSSSDDKTLAVWVTPRLEPFSSECVHTMTTFYEIKRSCDWWERDLDWITQDWMKVTGRPIEFFAAQTDSDGKAAPEAKQRLTHLSFEVSALFSSACCHTKFYHKDPTKGIFFQEVVGYVADRAWLNDAVLNYALDIITTSHLGVHVLSSFVADQRTFPSPPRAKLFSMRFVILPINIKSSNWTLIVVAVHRHGTITVHMYDPLCTTGYRKRMEKIWTAKLLPYLRAWHSQWESQVARQEEHPFPADVDIEWLMSPMQPDGYSCGVMVAAMAYSFIYGGRGFTVDAVTRDVVKVMRLRLLWVILCGSHVEPIEESLQIEAKRIGKQITAAFGKGSKKIWN

>PpCys15_PPTG_04889

MPFPPATVLCSKVLSGIYRQRLRIRHQQVTSSYLQHWRPFSVRSTMPPKKPPSKLTAQGSTLNPPPTAHNYRQSVQAPNLPPSDSIPPLWKPNVVPNEVFPEWMDPAALPADHWGTVEQPFEDPLAPENLVLPKEVIEFKDSIMWKRPSQFLPVIVETPVVHATPPPPTPEPKATGKKGAAPAAKKGEPEPQKKPPPPKHAHVFVPKSEKAGTTTSSSSLPSTAEHHHDHTLRIPRDFQRRWSVEQLEALEAWTKEEERIELEQQHRERMYMGFEDAIAYIVNDRPRDLLAEVDFDADDLIDDGQDEDDALTTNMDVSNATSPWGVMPPPRIEIAPNAVYKPEIPHGDVIHADMASYFRIVEQLYDSSKTSNNCMAPFLWQAIYPQDNTGQPVYNPGGKYSVKLFVFGRWRRIDIDDKLPLDADGKVIYLASSMKNEIWPSLLVKALYKVAYWLHPHRHETELERQSSTDGMCQNVIQTILALTSWKVSLWQPEASTKFSENVFHQLRQYILSSEKPDEASEQPTESESVANSTTGVSPAVTETLATVNALMPRAVICCTGINRVAGMVFGEIVLVTGVVGDTGSTTFKVVRQGSPATISEETNDVNELVFLLVHQVLQYSDTFIREWMPNPEPPPEGVEDTRAPLVPFETPRVQFVVITARDSIPEQPPGDNLSDISHMIQEPVNLVASLTPVQSPEKYDQDLDNLSKPSVLMATHLVVDPNGSVILIEEIEKKASTSSLPAILPLNSTFAEYISVPLVGKSKIVYRVYSQKSLRYGYSLVVESNQKVSFQDAPTYWRSLSNFHVIECDGAYPVMLPGTWNILFKQSFELVPPTHEDGDKEPSPELRIDLYLSEEMLSSFTHISIVNDATGEVKKASTLCTKVSLPSVSNLASNAPIAYTMIIDCAPGNFHVREGKWKLTLASDWDFTKPTTHEMKITQFEGVYEPNKPLLCFRDVIMAPKTSIWTSFQLQLLSDGAVVNTLAAKLEVFDLGMNQTQRISEISSKGEVRLLQLPCVTAADGQSQSDDKRGYIIQGSIDRTTCIVPDELQSRRPFRNNSNRPSKELTTTSVDENTSLEGNSGVSPPGSARSSRPSGIKWRLNCWSSEDVKLQADNAKELQFEAIRASWAEKAVDRNTNGAVSRLLYLGKLEEAEARMKQDNMTDEQIAKVRNRFEWIQAVRKAEGVCESYLEEVAGSEEKLLSKEELDESKRLLLERIGAVEADKEQRRVARATAKEERAKELKNMVRSVIDRRAISLKKQQELKRQLAALQTQSA

>PpCys16_PPTG_05047

MRHIIDAGSGRVSADSAVSGDIVLDTGRTELGAEVGVREECKHPFNAYFVKNWDNSRQIWCAFERQNACTLGNNTNNRLESSRKQLKELVDSFMQVDERIVSIMIYQAQAERKLLDALVSEHACELVYEQYNFVTITKKYNFHEAVPGVYFIRCDADDEDALDEPRSEYSVTKTDWTGSCLFMLLNSRWLLSSLRVESELPQLSEEPFRVSRVLKETNACSDSNRKFREANFVATSISEHLSGLGMVEYRAAMKALRDVATLFKHGQYTTIPEIAGEDSLSDANMNTTGGARHPTTDSEEVSEALEVGQVIPRQAEDVSQRMVSASSVESTQVCADQSGSVGDEPDSGDDQSGSVGDNLVSVAIETGADGDELDAEPAQIATEAERVSIESETSQDNDDHSSMLALANLTDDFQIMSPPKTKGRPKQKPRAVKAKWNQTIAMVQEDLDMHERQMSLLTVYELLDGEPVYKSTHEKLLQFKEFLLASKPKPPIAHEMSKLPPTKPLMRPEMVLALEIVGVGVFANSTISLMRKWHTAVKFIKKIKRAMTWIERLDFTRHGNSSFYVEEDPAIPVLLENLPILSNDVLVINPSNIGISNGAVTTDSGYFQRALAGVTKKMKVLFPINCNNNHWCTVLMDMKKGRVYVYDSMAPSYAASVRVVAQKMIMMLPDGVRPSARLVTHDPGLGVQSDSYNCGVYVLLAFEIFCGSEPPRQEDCNACDTAICARV

>PpCys17_PPTG_05143

MRASLIVALVAATVSASPLSLLERTSSGGFGYVRSPDRSVSLTSPRPHDYIDVSKLPKNFDWRNINGTRYVSISRNQHIPHYCGSCWSFAATSALADRILIAKERNPGNKPSVEVHREVVLSPQVLLNCDKKDNGCHGGDQLEAYRYIKEHGVPEEGYQRYAATGHDTGNTCTDMDVCENCLPSKGCFPQKAYDKYYVSEVGTTLGEQQMMAEIYARGPIACSVAVTDGFLKYSGGIFDDKTNATDVDHAISIVGWGEEDGVPYWVLRNSWGSFWGEDGWMRVIRGVNNVGVEGECAFGVPRDDGWPTPTKIEEKEEEKLEEPQEETSVESTLGGCRQKLHFAGGERVISPLPHETMDVQDLPKKWDWRDVNGKNYVTWDKNQHIPKYCGSCWAQGTTSALSDRISILRNASWPEIALSPQVLINCHAGGTCNGGNPGLVYEYAHRHGIPDQTCQAYQAKNLQCDQFAICETCWPSKESFTPGVCEPIKKFAKYYVSEYGSVSGADRMKAEIYKRGPIGCGVHATSKFESYTGGIYSEHVMFPLINHEISVAGWGYDEDTDTEYWIGRNSWGTYWGENGWFRIQMHHNNLGIEQDCDWGVPLPDGSKPNDFVVDYQGNQATDRNFLYVGDAH

>PpCys18_PPTG_05165

MRSAVKDNGAADLHTLQEELKKLGISVVSGRQDTVISVKIKGLAGRALIRGLKLADQLLQEKIKHAIKACTDPATKFFTDPTFGPCSSDPDGAAAICKAGGVIPSKGGSQHQAKVIGLLQRGKIRWERPIYAPDNEEDDISPQEDEDDIYAMSSSDNVFASNATLFADGVSSGDVIQGNLGDCWFLSALSVVATRSDLLAQTFWRRDQHKSKGLFVCKFMKNFVWNYVLIDDRLPVFGFTDKKPGKPYFARCRNPNELWVSLIEKAYAKLHGSYEALIGGFIDCALNDLTGMCAEQVILREGFPGFGENPYTPAKPQQKHGDPFWEKITRYKNSGTLMGCSIQPPVTSTKEVAVESSAGNGLYFKHAYALVDAADIKTAKGEYVRLVKLRNPWGMGEWTGPWSDSSDERAANEDAIDKFFKIMKRKVGANADKRVFMTLNPQGRVGADEISQEEVIEINANDGTFFMSFDAWMQSFTHFFAGIDFPDSWHGRRSEGSWNEANCGGNTTKSTWINNPHFELILEQRARIFASLSQEDPRGSENLKIVPVGFHICSLSPVGNDPNKFEIREPTKKLDAYYRSYKPVDRDNYMTGKRPEPLPPAIIPGTVIPGIDDDGVPQAAYTFKQAVSVEATMEPGRYCIIPSMYMRTDKVTGNTNVGNFWISVYSDKPTFRLEGGEKIVEEEEGDEETQTNGSQSYKNLNAGATTLTRQFSSTNSSRKSETIVATGNAVAAEKRRNFENEKEELLRQAKRYGIGLRELRTAFGKTPKVSKAECRQKLQKLGFNVASWDDDKIGLLFGGLEEGPTSGLIQTDRILNLFTPDIQEEKMACELPDMEEDGDTPDSIHREGILEIELHGASGLAVGEKRQSLIQRKVPIPTPTFSTSQISLRRKIAAEILERDPSMAWYLRQFAKKSTLLANTHNTSKQNRDGSVRSQLHIPTPVAAFYELLEKEYAARIISRCDVEDELEYVVPVSRTPSYSKRDGISPFYPSLSSKSAPEWNLPPTPLNELELHKSSRIRDLEAKRSTKLALLLQHKQQALGDAEKTASLPRLIGGRKSGSSHVKCIDCGARVLVGGRSKTATSQDIDSCPCNGANCVNVFCSPCYSLLPTKSKLCEECYQHEISPEERFGEQLRGVLIEKIGASDQQRSHLEEVFRSFDVDGSGSLSPQEFEKMLQLLNIQPTLTQRQKAFLVGQFDANGDGEISLIEFKHWILRDHVWVESVAPTSHKTIPHTAEVMSTVIAPLCEEIIDLAYNAYAFSSTIGSWSRSSSSTTECQKKTLQSGETGVIIFQRVIKLSVTSGGEANVAEKVFEQVDDDGSGSIDQFEFVALLSALGIHLNSEDAVLLMNRFGAASTGSGRITIEKAAFIEYVGQMSTKTFSRVGDGSLDKILVKLAEFAATNPVARESLQDELQQLHENITQKELLRICKNLDEKASLIGGVDELRSLAMNLAFDHEMNASNTEREGADQHGPLKSSGDILARMLLSESGDLMKLVRTFDVTSTCRELASHLQIRSGTNSTRTIWKSVFGADTTDVVHLRDFISCVLKTGFLIEVQNEPDKLGVATHDRLITPLLLSLAVDAVRTDCHVLSTSPEASPVISFNLFRLIVRLQKIKEIELKFDHALTNFMQLCQGEQQYLVTIALDASRNLVVRAGDPVFKFSAAFELREDEFDYQNLIAQLGSASFERRQQETHEVSRELAFAKAPYGLITSEFQPELNNSMLTIIERLRAELRCRHHSTTSHTSRLHILSLTMVESEAFVSTLRNLLSLVELPFFWSVSPRSLVFSIDSEYLDQQSKPSFQQLVADTLSLHGSQLPPPLRALATFVAHTASSLLVRYEVVGKYTNVETSWQEFQDFISGHQNAYAVMELHPQGSVLTTRVARASGSGAVQWNFKTQIKMDEPKLCDHRIDRPVVFTDTVKVSCIPGSTENINLIRGDSEGPGHFVVISVRRALPNGSDFPKPRLYCTAYDPLTSCDYAVEGYPSDWSVDFFDVTVNPSFESQWQTMLDSMRLGVTITPKLAITVYNKQPKTDKLIGECEVSIGSAVVQEGHIFEERVALRPPVMNPTSACAGVTSLTFRFDVEKTSSIREDVVAERRQQDTGTRVPIKLGAQYLAGEIVTPRTESVPDNGNDRVKELQTSMTAMEMSKLEAQEQVKQLKAQIQQLSYASNKTTEDNAERWKRRLEQARQEQFAAEEQHAARLAALQEEIRHLSEETQKQKNAHREASNLKDCRLSADASAYDIFSSIREILTRRCPERPYNGLKKALAAEAEVPGKVTFAVLNDVLGDFGLALSSDQRSTLANLFDPEMVGRVNIEGEFVW

>PpCys19_PPTG_05196

MLPVSVLSLGALALSTHFFSPGVALSEDAQQIYSEWKQSSYYGQAVQVVNTIGGTAAGSFSSSSKTDVSGSASSSGAVGVGSLESNETITFPPTSGSTGSSATTSSASGTTTTVSSITESEEQQRFNQALADIAELQKLHPHANFSINSPFVLLTSDEFLSYVNRYAIDPDSNPVKASTSSTAGMFTMDAEGSNTMTLSSSGGEIMTSSAAAGETVDWQEAGCVTAVKDQGECGACWAFSATAAMESGYCVATGSLPSLSDQQLISCNTEDGNSGCGGGYAAYSMDWVANERSGKMCTLDTYPFTSENGNVASCSMSSCTEFDVGVTGYESVHKDPGAIEDAVRNQPVSIFLYSGSTAFQYYSGGVLTGENCDKTGSHSGLAVGFGETDDNILYRRIKNQWGTSWGEEGYVRVQRRYSGDSEGACGVELYATWPTFDVSASPTPAPRPTTSAPSATTATPSVTTATPSATPLTTVMPSATPVTNTPSAIETVVDQAASASTASSSDSTTQKIGVDQVASSASASSGSTSAYEAVNTDAGSGNAVDDSTAPTKAPATPSVVQSDSVADDASYATVPSTDKRDCAM

>PpCys20_PPTG_05597

MGFLAALSVALLGTLLTYSPVNAEHWAVIVAGSNGYSNYRHQSDACHAYHVVRRHGIPAENVVLMIYDDVAWHESNPYPGQLFNKPTTKNASHADVQPVDVYKGCNIDFRAAEVTPETFLNVLTGNSSGAFNKKVLNSTKDDRVFINFIDHGSRGSIYFPHMKPLTASRLKKAMQTMHDKKMYKELVFYMEACESGSMFSDSFLKSINAYVTTAANGFESSWAAYCPPLDEVNGERIGSCLGDLYSVNWMEDSDLTDLSGETLTTQFHRVKNATTKSHVKSFGLSKLSHEIVGNYQSTYDKSYNGDDSDSEDIEPLSAAAARDTETAIESTVDARDVDLVVAFYRYLRAAPGKSRRGFADELTATIQAREVADEVFETITALYEQRTESSLLQVEEPKNLECHEEITRIFETSCAYSGGLTSYSLKYVGTLMDLCESSLPQDEVASIVHKACLVVETRRAPRNDVFNTNVAMLA

>PpCys21_PPTG_05949

MRLGLASPSAPQASDRHLPPAVAHRSSAFCPIDVDGDSTDDAKSPASRHHDLSALSSPNTLPSLLDLSSDTAYNNGTTRRQARALEIHSDDSETKQSEHKDDTESHSSSVRLSPNRHEETKKPALNFFFNIDRSTPKKPRRDYFSSGHISAHSSPVPDELHEIISAARTNICGQMFDRCELEFGEDRVRLTIWKDNKARWQGKVKYAHMARFCFSRVDHPPYILLMQLENSRGRSDFATFYDPIFARVCEENVAKDQTMPKVYGIVFYFDEEMNYMRCRSMGDNNPTLAHLFKEKLSESEVRQFVRLDVPSRPRRIEVKNSSSSGASRSLVSRVGSGARQAFGSVTSFFRPAPRPSVLEEDENPSVVRDRGSSTPQGRMLNLSRGSDPIPPDLDEEKAPISVDDAGIDKKKYRSSLADSGADCVVIIQNTQQDEAEKRDNSAASSPSQEEKASEKVESRKRAASSRREDEKLKRRRIESRRNEVLLTYPYDGSDTSGRISVTLGDVDRLVPGEFLNDNIIDFYLRFLWRHLPSWQQQQTYFFTSHFFTQLNGTNGAHELTTADPDERFARVTRWTQKETNLFDNRFLFIPINDSFHWSVAVFCNPGSAIIKKHRKVRRRRRMITARSSDTREVVDLVDGGDQHERPLEAASNDGDSVEEEIEEEEVLSCQEDRLAYPPCLLFLDSLRCHRKKKFTKMLRDYLECEWKARYASSAAANVSKTEDGVDEEESIVTMFDSESIGLLEPNIPLQSNSSDCGVFLLMYAASIVHCFPAGVTREDLENNLTSSLTPTMFRDEHVLEFREYLQQLLFSLQFLEKHGIPEEKVKDEELEMFTID

>PpCys22_PPTG_05985

MVAMDQYGCGQPVQYSLLETNADWHMAKCLDHFKRANDSWRLVRIVIVDKDMREVEVIRQKLPEARVLLCHFHVIKWLHETIRKSSKYGPYEAAVLNQLRHTITNMTYARSDEVYEEHRAEFKSLSMRGGRQELWDYFEKNWDNCKDTWVMAYRMELPHFNNHTNNRVESLFSKVKQHLKGHITMNASLEALLYQRRKKEEYYAKVEMPGTLRDFTYSEEMNIVLGMTTKWVAAAIRSQYVAVDPVIVSSYTVVDNGPTVTLRNEDHECLLQKETFKCDCEFSQTMQLPCRHAMIWRKSRGGPFVVPFGAIGSRWYGRSRLSTQGLSKVAKPFVAKVFKGDDAAISRDRTEKEKFRRAQQAFGRISGELARLGDEAFDAAMDQFEGWWHNLRQGQISMTPCHEQTENRDNDGSNDGGNACGGENAEDTSSVAQAISGGGGSPSSGESEDLPITQQTAVSTRLVRKPDVPVKIKLNSRIVPVGRPRLNRKEQRAKAKADLKEYNQGMKPRGLLRDRDVCEVVSALKAIQPGILEMGAFLATFQVLGKATPKLSTAWRHRTDIVADNVRYRLPEETVNRAFKLLSESRTGKKDEIQLDSDGEGVGDENGYVLVVEKVGTYSREQVEQMKWMWNMQDTCRRGVLLCTWLNNEVKTLVDEKVAVSVAFDNFLKTWPYLVIPGFGFDITYADSETYKNNATVMIPPVKKQTFVLMPINLSGVHWVCLVVDGIAKKIQVYDSAGSAMYLKRLKNIASEIASTLPDMYEEIVFDGPLQKDGDSCGVFACLQLWKSVSSKAPTDVSESGVIKLQNFAGYSKGEASLVVEKVDATVNVGV

>PpCys23_PPTG_06038

MPPRLRNAQKRPLSADDIDVDNDSNASDGSSVTSSAYQIFRVRTNTSVAVRNGRIDAQGSSASKLSAEWENYAKTYVCTHYGKYQSQATSKRPRQETRASGCSAQINLCVQEINKNTHTFALMITKCRLEHNHTLNEYAFKSHSSKRVSLYDTALKTVEELRKAGAKKTSILKFIKDNSNSNPTPQDVRNLVRKLKASENGSGPSSSAKRLKKLFAERVDTIGIDTSIAGNVMNGFLPVDTMKSVLLGATGEKILIPVICGKNHWCSIMIDLTCKDVLIYDPMNSSYGSKVRPLADKLVTMLPDFAPRKYRVRLYLSELGVQVDSYNCGMYMLLAFEVFAGANTLSLLSRKELQYLRYRYLCMCI

>PpCys24_PPTG_06097

MVGVLRAAPLLAVVTLASLVEAKDRCTAIIVGAKASTTGTPMTTHTNDCSSCDFRIAKVPAQTHEDGAQHDVVLAAFDYPRYVGGARGKEYLPENLDTRFYNWTTTPAIGSIPEVPQTFAYIEGAYGIINEHQVAIGESTCPARFWTKPITQGGEALFDVGELSRIALQRAQTAREAIQLMGDLAVQHGYYGAVWEGKDVYDEAGEALTVTDTKEAWIFHILPDDTGKSAIWAAQRVPSDQISGVANQFVIRELDLNRPSDFLASPNIHDVAIRNNLWKPDEGTPFDFTRVYAQPRKDTHQYYSTRRIWRLFTLANPELKLSPETDVLASDYPFSVKPASPLSPRDIMRFQRDHYEGTPYDMTKGPKSGPYGDPNRYDAAPNGDLTQEDIDRGHFERAISIFRASYSFVSILDPYNPDNAFLWFGQYAPHATTYTPVFVQSTDVPKQLARGSLYAFDRESSFWIHALVGNWAARFYSYARPFVARVQDEVESHADGMLRSVLIEAAQHKREGGVPAMVEFLTKQSETFAQRAHRASSDLFDYLVTAFHDGYQVSNFYAKMLTVQSIFYPKWWLQQVGFFDGAEDSGDAEPDSTETATTAPASSSSGATATAKTVIVEVAHKGSVSYFTTTVLVILSGLAGLFLGRKFHGPLTNKKQGYRPLQ

>PpCys25_PPTG_06374

MQFTNRRVVSLDQNRSSSRVSIMPRRTLAIDSSSSALAVEDEVIQWLSGQIDETTGSVPLYEDPDFPPEISSLYFDPNKLPDYVPQQSRESSGHVFWYRPTEYAEDPDYFKNSTGCGDLREGLLNDAWLLGVFAAVALHPDNLIENLFVSESLQNFKQFGVFTCRFFKDDNWVPVTTDTRIPYSMELQPEDKVSSGGGSTFSPGSALYGSSVNRSEVFILFLEKAYAKLHGSYQVLDEKYGGGIGGSAGTSSGRILEAFLDCTGGSAHRVDLQQERIKQLQFDQATAGNSSAAASSPSILLWKQLLRYKKKKCILTTQLRQLSFNAQDVTAMGIVKNRQYVILHVTEVGLPSGQGGSASREEVLRFVKLKTVWGRGMWKGEWSNDDSKWEEHTQVEQVMRSDPRCEFSRSGHDGCFWMVWEDLLDTFTELFVVHIFQPEDRQYSVRGDWVGTTAAGAPVKMPVPTAGDDHNSSPHPGRSKEILIEKTRWGWLHDADPNWHRNPQFKLTVPLESLSTGNDKTVDKSVNSVLLSLTQRDFRLYGGDNYAINFVLLREKLTLATAASNDPPPAIWEFKRSHVVAEAHSYETSSAIDLPPATPATPALSHPATPMAGVVANAAGMSAPVTSGQSKTLPERELVKEDVTIAPGAAYYLIPYTTNPKVEMEFFLRVVAPQPVRVERVPPIMSVIRTGRWRADDGAANNDTNNTSGGTSSTGEMANAGGPLLTLPLPQSHVVGKENPAWCQNPQFWVRFAERSPRELRRLRKLLSAKAHVTIKVVLRKTSHRASLGNKSRQQREAAKDRANLVGITAVRAPPTISTVSVVVGGSGSSAATQLRAHAKGQKTNFLGEIVDSPFTKAVAVSDNLVQGAKSRNQKAEKTIADDLDDDTEAPEQAEPGSLSGNFPTPKLVVKPTEWCRLSSYSSPASACLYLRKVPKEWLLASDSPSAVNSSGGGGLLLVPTLGEAGAEGSFELQVDSDFPLLVDELPKGAGAVQSMPGEWTPTKSGGCHLHPDWRQNPKFYLQIQGVRPTKVRITLTRSEREWKTRCQRDSVGTMIGFYLFRGGSGKLMRPAEEGDSNSNPIVINGRPWSETDFVPLHSVSSPPDLVLSAAAKGGYVVVPTTYEPGRLGKFVLSVQCDTEFTLTCDAE

>PpCys26_PPTG_06979

MAERQYAEALAATEQAQSFERAGQAAKAVPFFLQALRILKRLAVIESAAQRARLQPTIAELTTRVSELQKSSDYLLAHAMEQHAAAREAEKSDSTKTDIVIDRYIAAAETYMQALNALPTSEVAARAAIKEQLEYVIDYVSQLKAQTQTTRHQIQQQVEDNSNDVDVAALQWPEPPMLQHKTAHVTPAYASAAMPAAKPAPEVEQTKEEKPGAAYTPQELDVLRRSSQINGHLFVPWLDDLDAQEKFSLPEPFEDPDGHVPLSAKQKKKEATWMRPSDYAAICGHAPVMIAQGGVNPLVVKQDIVTDCSFVASLCIAAAYEQRFQKHLITNIIFPADPRTKQPVYNPFGKYVVKLWANGVPRKVVIDDLLPVNASSGQLLSSCTTRKNELWVSLIEKAYLKLNGGYDFPGGNSGIDLFALTGWIPERVPVSELIDAPNKEERLWEQLKSAFHYGDCIITMSTGDITKQEAKAIGLVPMHVYAVLNVYELSNTSDSSQEGKKVRLLQVKNPWRKMSWKGPYSRHDKTRWDSAIGDELRAYQRQFYASEATESEEASGQKDDGLFWIDFESVKQYFESLYMNWNPELFPYKGVFHEHWPVELGPVNDSLTLGFNPQYSLTFCKTAPSQEGATAAAGSCTVWVLLSRHVSLIERDTDYSNQQFLTLHVYRGTPGKRVFYNHRAVSRGMYSNNPHTLVSLDLDLADDSERCFTLVASQYEKFAALDYTLSVFSTRPFTCEPNPQFVTRAPTSIVIPGAWDSACAGGRPFYSTFMNNPQFHLQLQKPCRSLFLFLETEAFFSQDTTTASFPINLRAALHTRERVCGLHAAGNDEEQLKVLSSGEYRPGFCFIEVDSAVLASGLHDVVLIPSTFEQGVLGKFTLRVVSDPPAGASIAYRQIPAEGHGMELTRLRGKWDMQTGSAAGCSNYGCYTFNPKYLVHVTQECDILARLVVLDPEVDAAAGESPTEEESLPPPSINVSVFESTSEGDLLLSTNPKQAFAGATSAGGVYVSGAPSGALAKASRRFPPGWYIVLPSTFEPQDWSFELRLYASAHVDARLL

>PpCys27_PPTG_07405

MARQVLNYHDVQLYESDVALFAGRQWLNDNAVNFYLQYLTQTVASRDVLLMDPAVVSCLQHQCEDEDEYQDLASGLNLTSRQLCIIPVTDNDSLGGDSSHWSLLLYRDGMFQHFDSSSGHNKHAARRVAEPFELLLQAAGRYDGDGASRHVEEVQDVPQQQNGYDCGMYVLVLAEYFCRRHAGEMETVSLDDYATPKRVTELRSQMPNLIERLKIAIVQYDSQLGQVERNMEVSVGAAFKAIASRYVKLTDPLFDLQYVDKMVVLLSEEDKIDVLMLSEMAFTGYVFKSKADVAQVAEVAGQGPTFHWCQRQARRLHCMVTCGYVEKEGELLYNSMLVVSPEGELVCNPRKTFLYETDKSWATAGNGFCTWHCPWLNKTISFGICMDINPDDFKAPFSAYEFGTHVVENKSDLVLFACAWNDFEDHDVEPYRTLSYWAERLSPVITALGTGEYTKPNCHFLCSNRIGSENGTFFVGASCVLSLKEPAVVAHAGRRSEELLRVEVPDEEIAAGE

>PpCys28_PPTG_07663

MQLALEIVGVGVSANSTISLMRKWHTAVKFIKKIKRAWIERLDFTRHGNSSFYVEEDPAIPVLLENLPILSNEYADMVDLAAKETLSAQVMQMALHKLFDADPSVLVIDPSNIGISNGAVTTDSEYFQRALARVTKKMKVLFPINCNNNHWCAVLMDMQKGRVYVYDSMASSYAASVRAVAQKMIMMLPDGVSPSARLVTHDPGLGVQSDSYNCGVYVLLAFEIFCGSEPLGHLDKKTLQCMRYRYLRMCMKEEGSSNSSS

>PpCys29_PPTG_08165

MGKKKSKTTLPSDDLEASASESLLPSSDAEYHRLEKHESAVLRRQRSKSRRIWCAIIALTTGILGFIAVLAILRAVEDASHLSPTKRPMFPTQYEASVNFNMPYMNMVEPLYVHVDEAKGLQKLSYYGGTDVYIFNTSGTSYQIIPVIRERKCFKFGSESLQHIFPNMTLFEPQHGVFLVEGRPCFSWKFVTKLHEPTQDGLLGEYTLYVDQKTERPVRFHYVGRNGMLGGSHIDEYYLDYIYVREGPVDEDIFSFLPASMNCTERPGDDGGPSRNPKQDIHMLLPEGSTVKKELFDNFSASHDKEYNDDAEAVQRMATFHHNLRFINGENRKGLPYHLEVNKFADLSHEERLALHRPSRVKRAKDNGAMAVHELTSFEDPGDMDWRTKGAVTPVKDQGACGSCWTFGTTGALEGALFAQQKKLYNMSQQNLLDCSWDYGNNACDGGLDYQAYEWIMANGGLETTATYGAYRNAPDYCHFNVDNAIGRMKGFVNVTSVQALNDALATIGPLSVSIDASLPSFYFYGGGYYDDVECKSDLDSLDHSVLAVGVTTHNGQKYTLVKNSWSTHWGEDGYIKITQKDNLCGVATAATYPVLAD

>PpCys30_PPTG_08264

MQATCRDAVQCCTWINSDVKTGIADPVPARIVFLQLLEAWPYTQLRGFGFDLAYSDLYCFRGSAWFNDNAMRAFAVYLRRYKNNGAIALPQNSGKTVSLHANMLNHIAEGVELHPYVLLPVNFGGVHWGCLVIDKAAKKIKTYDSMGGKKNMKFLKAMTSEIIEKTTGGDKFTNTTVTEPIQTDGDNCGVFVCLCSGLFK

>PpCys31_PPTG_08268

MALQEQTHSCNHDGSKDSIDASTDEKDSIDASTDEKTDGAKDTSGTICGGGSPTSEESENVPLTQKTVVETEQPASGAELPVEIKLNSRVIPVGRPRLNRKEQRAKADLKEYNQGMKLRGLLRDRDVCEVVSTLKEIQPGIREVGAFFATFQVLGKATAKQSMAWRPNANYVADNVRYRLPEQTVDRALDLLRKLLCGKMEEIQLDSEGEEISDANGYVLVIEKIGTYTREQVRQMKWLWNLQDTCRRGVLLCTWLNNEVKSLVNEPVDVSVAFDDLLKTWPYLPIPGFGFDVAEADLFCVRGNTWLNDATMRAFCVFLDTYKNNTTVTIPPVKKQALKKPVKAEPILADQQLQQIHSGLTSRQYLLMPINLSGAHWVCLVIDGISKKIELYDSMGSAMYLKRLKDIADEIVAALPDAYEEITVNGLLQHDGDSCGVLVCLQLWKCASAEAPSDVTKSGITKLRWKILQGILKVKRRL

>PpCys32_PPTG_09001

MARTHKTAQIKAQMAEKARIEEEEARRREEARIADLDSARTRSEKAGALVRACERQNVGSTDSSTSGANALVVDKEPDGEESAGEESEDEVQEEEESEIEDDEDKEENEEEEGELEEKPPTSRDTKGKKRTLSDVEDEELVRVPAFKKEHDSWASLEFSLREYMNTTHQKIVIKEVIHTARRNAALRSQVRFKGRPDSDIPLVPAELEPYQRKYICTHGWSERERSTGKRTSHALRRTDCPFQMLAQLAKKTDGSWCVSIKREVYEHNHFVSEDIYRFYPGIRQVSADSPLMPGVELLVEAQAGTSSVYDYIRSNSNHRVTMDDVRSMISRLRSVELSDNDAVSEMIVEFNLESSKNVSTVNENARGDTGVISFTSGHMRAMLNSFPEVIQLDCTHKTNQYNYQLLSMVAMDQYGQGQPIQYSLLETNSDWNMAKALDHFKRANERWKSVRIVIVDKDMKEIDVIRQKLPEARILLCHFHVIKWLHNTIRNSRKFGVYEEEVLVQMRHNITNMTYARTVADYEKYRGEFEHLACRGGRTQLWTYFKKNWDSLTEMWVMAHRADLPHFNNHTNNCVESLFGKLKRTLKGKLTMRTSLEALFAYQKRLEEDYRSKVSMPGTLRDVAYTEEMNIALGMTTHWVAQAIKTQYDVAVNDNLASAYAYKDNGSTVTVTRDNHEFLSEKDGRTCDCEFSQTMKLPCRHVMAFRKACGNPFIISYAAIESRWFGQSRCSPDELSELSDPFVAKIFKSKRDATPRNAGLSEPQKYRQAQQAFARIGDELARLEDGEFVCALQQLDQWWYNLRQGKRLKGARTSSEDEVKTQYDVAVNDNLASAYAYKDNGSTVTVTRDNHEFLSEKDGRTCDCEFSQTMKLPCRHVMAFRKACGNPFIISYAAIESRWFGQSRCSPDELSELSDPFVAKIFKSKRDATPRNAGLSEPQKYRQAQQAFARIGDELARLEDGEFVCALQQLDQWWYNLRQGKRLKGARTSSEDEVNRETENCNGGAGAENANECSDSEAPTQKATEGPPFSSVGRLKGGSAMATSVPTVPLSGKVRRVGRPKLNRAEVREKTRVALKEYNAGMNLRVILRESDVCDVVATLHEIKPAVRIVGSYLGTHQVKTVHQNRAMKWSVDEEYVASRVRFRLPESVIDDALNLLKNGIGRDEEIELGSDGERTGDITCFVVTIEKLGQFTREQLEAMKWLWNLQAACCNAVLSCTWLSSVVKPVAEQPVAPQVTNLQILECWPYAELKGFGFDLTYSDLFCLRDSVWMMDNTMRAVTVSLGRYKKSLSVMLPPPTGNPSEELQSSTIDEISQATTKRPFVLLPINLGGVHWAGIIVNRAEKKVMIYDSLNGAKDRKELRKLADFITTKALRDESYLKVDVTEPTQMDSNNCGVFVSLFFWSAVSDEAPSDLSPTGLTKLRWSLLNAILKMKQR

>PpCys33_PPTG_09911

MGRKVVKKLAKKAPKVIFEAATGKRLDDVVAKTKAKPKKKQKSASYVVDDGAIELMRRQLVDVGCKLHEVEADGNCLFRALGDQLYGDQHQHEEVRKKIVSYLEQHRDDFEPFMEDEEKFEKYCERMREDGTWGGNQELYAAARLFQVYVVVHQDQPSARIMVIECDRLKPTRFVHVAYHGEDHYDSVRALKDPVDPDSLPVSIELNWDGFRPEDFAK

>PpCys34_PPTG_09912

MTTRLSSRFSLRSSSKSTKRSHKGVRFWFSSKTDNSGDDLVAAAAAQAVDEQLQKTKQRSESGCSYVPRVEYGSSHCGVERDAPWTSNEQVEQHYSALGWTVVPIGKDGNCLFRAISDQLYNNELFHQDIRRRLVDFIEREEKHFQPFVDGEQVDDYCARMHEDGEWGGHLELYAAARLFNIHIVVHTGPVRRLRVTNEDVDKHNLAPPPYRILHLLYKDDHYSSLHSNDDVKTIAAEEYTVIPTDKRVRSPAKTFDSPDKKVAKVQETKVVHIQDVAKKAKTSYDPSRKNVKFQEVVEAKPSPRESKAFLYEEENLPECGVLLPKQVLFKRGKRRVSGATLFSLVHIPSTGNLKPVVETTSVSTSSDASTATSSSEEQEDPSEMVTERPKPKAPTLRAVAIAYPRRTTFHKGRATTAC

>PpCys35_PPTG_10152

MARWTLSLWVPLLLALGCVCGEEYHRHTNNWAVIVDTSRFWSNYRHVANALSLYHSVKRLGIPDSQIILMLADQMPCNARNCFPGQVFNSRTQKINLYGKNVEVDYRGSEVSVANFITVLTGRHEPGTPASKKLDTDENSNIFLYMSGHGGDGFLKFQDFEEMSSQDLADSIQEMHVKKRYNEIFFMVDTCQAGSLSNALESPKVVTIGSSQTGENSYAHHSDFELGLSVIDRFTFSTLDYLQRMKVGDSIRNGTLRDLFNFYDPKMLLSTPDYRTDILGRPIDEVPITDFLGSMLDVHLHFDDEAYPIEPAPIETTACSAEALEIDDPVKVLNSPAQLSGTSTSVNLQSFEFSNEFFLGVAAAVIGAVFVTIKAI

>PpCys36_PPTG_10195

MQKLHDHVTVFVTQRELQARAPKPDGTTASTTALQYVAENSGFDVVTFQVDFSESANIQLHRPAEPGSTTQQKATQQLAFNCELRPFERRVLAYVIKKGKQRALVRVHYAVTAVVTPSVDAILEAQERAARMILKQQQHSNHIFSHVGKWQTLPYRPAAEVIRACDEINRYFREFGDVFVDMSFPPQAKSLYDTDAPNKVSEADSAIYSLCTWDLIHNIIDSSWTFVAHDSASTVAFTSGLPAQDSFLCALTIIAPHYELWLHQWFPTLDTSGQFDKVAAVPVALCDRGLTWQHLAVDLFFPSFPLGRGLMTPRNIFGDWYPALLHKAYAKLKGSYAAVSNIPTMKILRELTGSPWVYCYRRQDYENDTDPSTGKMRLQEVIQATQSYRPKSDEVSLLVAVTCGKLDNHHRAFRLTISSDEDSSTTSVVLHDATGKYLRQKIADSIAEFDRDQDPSALRVSWEQLFELEPAVWSLPLGRRCEQRLRRILLHEEDIGTMTAVISVPTLTTITFSSLYLLHFTTEFCDQQVDVDVNVVTVESDFSLALMENNNKFQDLSESDDGVSSKQVVRTLSAGEYVVTVSAKLPESRSKAENAALTPASVDANLQLVFDCLDREGKHELSEGDITSFLQLYENVASSQHGQEALRSFLHQHGSSNSLERSLTLDDIRELYLNQAIQDCHEDVVSSSVSSRAISSVNVRARFQELVWQDLLRLLPSLDVAEKPRTEIKCQDIVELVCCFHSDTPLLDVVLLSEDKSSFSSNNFNI

>PpCys37_PPTG_10258

MADIEHESTEPDWPVGPLLSDRMSVLCLKAEYVGNANPNFMHGIDSLNARYEALRRVRGDGNCFFRGFIFALCEQLLPTGGAGGEDSNAALRSRIQDKIQMSKSELVAIGYSDVAIDAFWETFVDYLAAMETRSHAELVQDFQTEGGESEYLVWYMRLLTAGYMKKNAETFQPFIDGLYPGQTVAQFCAAEVEPMGKECDQPQIAALTEALQVGVKIEYLDGSAGPGEDLQSYVCSPTVEATTQQEPVSITLLYRPGHYDILYPRESEEKEAE

>PpCys38_PPTG_10491

MLEPLLQHLSELSSADYYQELSAWKETVDIGLRVGEKGTFVSSNDEGGEDEDTKAGNFSLLDPVEAMGTACLMETLETSADSADITDDSSDGETVPPTQPSEITKETPKDSSDENLQDEGDGEDMSIDVNASHASVAPPARQVDIISVPKPKGQGRPRTTTKQLRQTKIATRLAVHKYPSNLTVQLDEFVIWARNTSNLKVVADMMDKYPIQLEDAYLSSRTIQCSWEAMRTGTYMHPFVIPADLTRSMEAAIKDAKNEQRGPGPLADKIKKQGIGLDIVASIDPKLWKFSGKYVGALTAFYAVKSNIRSWFDDRKWLERDWRKVVSNVDLFGAETGMTGLSSDAVCNRHWAMANEVISKFTISRLSSEFAPKSGKGIIAFDNIVGGLCRGWLNDSPVDFCMESLSEVVEQCYVLSSLTSAVGWPRLPTSPIIATRFILHPFNLKQNHWGVIIARLRYNAAADRLKVHVYLYEPLIDDEYHEDMEAEWNGITDNENKLVKEGLRGFVERWCQASAPNTKLEVDPIKWVEKPQQPDYASCGAFVVGQAYSYVTENLLWQHTKVSKDDVQVMRLRMLWMIMCNSRERRRSRATTEKAKAIGDKLLDELK

>PpCys39_PPTG_10776

MWGGRFGSICKATLLATVLSTLLLNPVDAEEEGCRCKRTMKGREHLHPKVHPLPTMSLDEVQELEQLPKHLDWCERGFCVPSWNQHIPQYCGSCFAHGAMSSAQDRIKIANTRRKYTGADVMLGRQSFLNCAPGHGLSAGCDGGEASDVYEFMRLYGLPDESCLPYNATDHTKFHGTNGTCPPHGYCMNCMYTPESKKVPQCFPVTKMVRYRAKSHGHLSGELAMMKEIMKDGPITCGVACSDEFTFKYKAGIIDDKTGFMDIDHDVEIVGWGEEDGVKYWHVRNSWGTYWGMNGFFKIVRGKNNLGIEADCAYMMPDISDEELVWEEKSVYGGSIFGIVPFKDSAKDHPIKDTSEDITSPEERLAAHYLPETESYATEPKTHHEDNSFAALAIMFFVSGCVCAALTALIVLKFRGHRYVYRTIP

>PpCys40_PPTG_10928

MARTDELKASNVEVEVSKQHSSWQAAGNNSRFVCQSIGSMLFWVAVGGAITSMLLTTQKEQNSNNLLPSTKIQTNKGSGWYLGGDDPEHVLSSLPEIRSLDELPENWDWRDYNGTGISLTTSVLNQMVPRACGSCWAFATISALSDRIRIAKFKKTGRLDAEVILSPQVLLDCGMRSFGSCRGGDPRYAHKWIYENGIVDSTCSPYIAAHPSWIGSGECAATQCHTCTMNGDCFVVENPTKYYISEYGTLNFTTSEEFQLQAMNEIYHRGPIVVSLYSLTPEFKHYTGGYILRDFEKCPGTTHVVSIVGWGKDAKTGVKFWAVRNSMGTHWGENGYFLIERGNNTYNIENKGAWAVPIV

>PpCys41_PPTG_10929

MAIIDKSDLPLLQPRTSTLLNTNNVSEQSLTSPLQSIRESFHADRFKLRSIGSVLGWIALGSFVSSAVLAPKQLGDYTHNGMSMHAVQHQLDLMAACTSTGSAKGDGDVEEVQTNKGVGWFLGGNDPEHVLTSLPEVTSFDNLPENWDWRDYNGTGVSLTTSVMNQMVPRACGSCWAFATVSALSDRIRIARFRKTGRLDTEVLLSPQVLLDCGMRSFGSCHGGDPRYAHKWIHENGIVDLTCNPYIASHPSWMGGGDCAATQCHTCNLKGECFVLEDPVKYRISEYGTLNFTTPEEFQLQAMSEIYHRGPIVVSMYSLSPEYRQFKGGYILRDSTKYPGTTHVVSLVGWGTDAKTGVKYWIVRNSDGTNWGDRGFFLAERGVNIYNMESHGAWAVPIV

>PpCys42_PPTG_11304

MSGGGGIDGDGRTENSSGEGATSSRCGATSRTRASSRPRRGPGLSEMTGVSMELSNSGDGALETGNHRGGMSGGGEVEKTEEGVGDDQDSDSGGSSDDAEGTDDASGDELVHVEPPAETMSRSERNKRLRRTKKGADDSQLVPDQFDPYQRTYICTHGWKKRKSRGEGSRPRQHIPLTDCPFRFVVQWNLSRGELQVKNGCFKHNHRVSAAAYATYPTSRGVTNPLVGARVDGMLAGGAKRSRIYDYLLDHDQNVIQVDVDNLVREHASSVASVDDNDATAREIAVFSAADPENVSFVAETESGETGVISLTTAHMRRMFGRFSELLMVDCSHKTNRYNYQLLTFMSVNEFGEGCVVQQSLIKPNGDWHMERAIAHFKRSNPTKIDQLRVIVVDKDLNEIRVLETNFPEARLLICHFHVIKYLKEMRSKPDFGKISGEDASQVDALVHKMVYARSEENYDETCASLKGLCERIGIQGFWEYFDKNWNSCQDRWVMYRWQNLPHFKTFTAVSQDDDIVEVWGSTRHYTLRLDDWCCDCEFSISMSLPCRHAIAYRKKEGVAGPLIPWNCTHERFVIWTTPTKQLKKVRQFAYDQFRGAGTRPEPKPLRSQAERYREAVRATHLIANELADIDDEDEFNTMLEFILSQWRNVRQRKIANTQADDADDAREGIQSDSNPARGAQFDDAAIKAEFEISSSGDDDTGSNEDEESIGVESKVKIRLNPKTRKVGRPKKDKSKTVVSEKVDRQWFEAMEKGRKTAGEVTLEALLAALLTEEPTLSETQRRLSGVMVKHNGADLKKPQFKMLKNPVLIMDAFYILPPKLMDACMKVLPLANTQSSAISVDEDRTKKKSQTERLTECVHIKGIGTFLRAQIELFKRVQNLKDTVQLGVDAHKWLLEGGLPSLPAGYHDLVRKVADDVEATFPYRLIPGLPQLEDYQYSLLYRAVPGIWLSDAAIRAVCERLVTDFPSCRFAGFVRAETTKKRTRNTDEMLVDSIVRNRISTQVAESGVDTVFLPLNFMNYHWCCIVIKVQAKRIFFYDPLNQGPYRNSALAIANYLKISGLSDYDVVSQNNPLQFDGSSCGVYVAWMFIRQGTPVPALDMSKFTLPRRRFELFYYLLTGRLLPIQPVQAVGGNLEDEEMEEKPPAPTITQQRNEEEEVPPTQVSL

>PpCys43_PPTG_11309

MNTALLLLLAAVAATDAAVPVVPTDRMVQFLQEKPDLESELNAWKQSDAGQYAQQNGFVPASSSRDVAAAADEELRRFFMTKLMIEEAEATNPEAVFSTNTPFTLMTEDEFVKFIGESYQRGSGALAATSFVDAAPSNSTDPLSTDKDWTTSGCVVPVKNQGQCGSCWAFAAVAALESAICLAGQPLTPLSEQQVVDCDKTSYACQGGFPGDALAYIQQSGGACTEKAYPYVSGDSGDRDTCKSSCTREAVTIREVVAVPESDAGLVQAISTQPVAVGVAAGNPTWKQYKGGIVSSCTSSELDHAVLAVGYSPSYFKIKNSWSTQWGEEGYMRLKRGAGTSSSGTCGIIGPKSVYPQL

>PpCys44_PPTG_11311

MNTVLLLALAAIVTTADAAAPAVPTDRMDQFLQEGAALRSELNAWKQSEAGQYAKEHGFLPTPSSKNTTDEELRRFFLSKLLVEDAQAANPDAVFSTDTPFTLLTHEEFAKFIGESYQRDSGALKATSFADEVPLNLTASPTDKDWTTSGCVAPVKNQGDCGSCWAFAAVAALESAICLSGKPLTLLSDQQVVDCDKTSYACDGGWPSTALTYIQQSGGVCTQEAYPYVSGDLGYHQTCKSSCSREPVNIRKVVAVPRTDAGLVQAISAQPVAVGVAADNPTWKQYKSGIVSYCSTTQLDHAVLAVGYSPSFFKIKNSWGTQWGEDGYMRLKRGAGTRSTGTCGIIGPLSVYPQL

>PpCys45_PPTG_11313

MNTVLLLALAAIVTTADAAAPAVPTDRMDQFLQEGAALRSELNAWKQSEAGQYAKEHGFLPTPSSKNTTDEELRRFFLSKLLVEDAQAANPDAVFSTDTPFTLLTHEEFAKFIGESYQRDSGALKATSFADEVPLNLTASPTDKDWTTSGCVAPVKNQGDCGSCWAFAAVAALESAICLSGKPLTLLSDQQVVDCDKTSYACDGGWPSTALTYIQQSGGVCTQEAYPYVSGDLGYHQTCKSSCSREPVNIRKVVAVPRTDAGLVQAISAQPVAVGVAADNPTWKQYKSGIVSYCSTTQLDHAVLAVGYSPSFFKIKNSWGTQWGEDGYMRLKRGAGTRSTGTCGIIGPLSVYPQL

>PpCys46_PPTG_11516

MREYKAAMKALRHVASRFKHGDYNSIASQQQDHSRTSNSSGLSDGLSDGEVLNELQVEGGAYVDQGPTETHGAASPLSSSVQTTQLGVQLGTDPAELGTDPAELGTTSVGLETDSTQMGSENTQLGSENDLGASTTLLGTNTTPNDEQARSVDLGNSSDLFEIVSPPKARGRPKQKPKAVKAKRNLAITMVQEDLDMHENQLNLVSVYEILEDAPTFTSSHVRLLQFKLFRFLSKVKPPIAHEISKLPATKPLIRPDEICRIFPMELIKKCMAKVTAYQRKHPRVQELDIAFEIVGIGVFANSTVALMKRWHTAREILKKVERTLMWINQLDFSRLANNASFNATADPNLPEKLKNIPIFSSRNQLLDLAAKECLTEDTLHLVLSKKFGGDPNVVVFNAATLGAVVNGKIETDKEVIRNALSGLSKETILFPMNCNGNHWCSVMMDLNKGKVLVYDSSSSSYLCSVRAVAQTLITLLPERAQPSVRVGTYESGLGVQVDSYNCGVYVLLAFEIFCGAEPLGHVDKKTLQCLRYRYLRMCMET

>PpCys47_PPTG_11601

MEVIQIKDVGSFSRPQIQLMKKIDCLKAIVQQGINTHKWILEEGLSALPAQYHYLAKQVADEVMNNYPYKNINGLSGSNELVYSMLYRAVPPAWLSDACIRGLAIRLVADNPSARFAGFQTVTTRTSRARAVGESLVSSDVLARLIHQIAEVGVETALLALTFHNAHWCCIVVKVTDKRIFYYDPLNQKPYMRAAKEVATYLKHNGLAEYDVVPQNKPVQFDQMSCGVFVCWMFMRKVVHGLTHDINDNCLAQRRFELFYYILTGHLIAPIKNTPATSDIEMQQTPLKDRDSTPTQDESGEESLPPTQVAG

>PpCys48_PPTG_12146

MRNPVLVLDPFYILPSKLLDHCMKVLPVGNTLHQAVCVDTGSSGDAGPTAVMEIIQIKDVGSFSRPQIQLMENIDCLKAIVQQGTNTHKWILEEGLSALPAQYHYLAKQVADEVMNTYPYKNINGLSGSNELVYSMLYRAVPPAWLSDACIRGLAIRLVADNPSARFAGFQTVTTRTSRARAVGESLVSSDVLARLIHQIAEVGVETALLALNFHNAHWCCIVVKVTDKRIFYYDPLNQKPYMRAAKEVATYLKHNGLAEYDVVSQNNPVQFDQMSCGVFVCWMFMPQIRALLLYLDRTLDSADQKYPSD

>PpCys49_PPTG_12287

MELNEFADTTWDEFQSWYLGAPQQCSATERNGVVYGEVPVEKDWRADGAVSPVKNQGKCGSCWTFSTTGCLESHVKLKHGEFTILSEQNLLDCAQNFDNHGCNGGLPSHAFEYVKYNGGLDTEETYPYEAKEGKCKFNTYHVGAQVDQVVNITARNENELKAAVGSTGPVSIAFQVVSDFRFYKSGVYESKECHSGEKDVNHAVLAVGYGVEDGKDHWIVKNSWGTKWGMDGFFQIARGSNMCGLADCASYPIVV

>PpCys50_PPTG_12342

MELLENWLYSKLAGIGFDYTYAGLYCFRSGLSSSIVDTGDVVTDVISVITLVKIRECLDNDLTGYIFIPVNFDTNHWACLVINKLKKKIVVYDSMNKRKIGKILKLMAREIDGGLLESAFKHLTMTTPRQKDGDSCGIFACLQFWRQVSNAAPTDVSSRGLVRVRWEMLQALMNQKAQ

>PpCys51_PPTG_12477

MAQFAHGISALDVTPSAALSAPVWLLGKRYDDVAAADFDAYKRSFESILWFTYRRDYPAMTPYEHTSDAGWGCMLRSAQMLLGQALQRRILGREWRLPALFETEIDARLPETYVQLLRWFADSPDVECRYSIHHMVKLGVQYDKLPGEWYGPTTAAQVLRDLVNLHRREFGGELAMYVPQEGVVYSDDVAKLCVSHIEQETAEKEVKDEDAPEFFDPLLHPPTTEDKSDWSTALLILIPLRLGLDQVNERYVPAIQKTFAFPQSVGIIGGKKGHSVYFVGTQQDQLHLLDPHDVHPAPELNAAFPTATHLRTVHSSRPLVMNVTTIDPSLALGFLCENRADYEDFERRVRILHDEVKEEGGMCPFSVAARRPDYAASDGDLLMADCLSGDDEVSSAGGAGAGEDDEDDYVLL

>PpCys52_PPTG_12597

MVSPQAHLQICPNIDTMFCGECGHRWEPDEIASARFCAECGAPREDVPPPASAPAPVPASTSSFSSSSSSFTSYAAPVSTATSSSSYSTPSYTSAPSSASDSSPSTAYSAPSIASYSAPSTSSYSTPVTSSTPSYAAPPSSATSSTKCTQCGEAFGDSTALFCGECGAHRPSAAPAASPAPAAVPNDDGGDLFGVASTSYTAPPSTSQPMTSTFAASASVPFNPSYAAPAASANDPYAPPSAAAPPAASPAAPVHDPYAPPAASPTYDPYAPPQTPAAPVEPPASVNLEAAAPSGSSISTLLANLSVSAKPEGERDEPDGKGVGVHIRQVVRARNMDSLSTRLEISNAVKRNRTNPQERTEGYNVTKVNDTITVVQNERADPVEERYYNQRTMPDNGEWLQILRKNRQAGTKFTDPQFPPDASSIFRDATNPKYPHLQEYIWKWKRVTDFFNETAYVEITMLDDDKKLVCSMSIKNPAEAESILEVIRQTPTSVDPQFLKIAKDAVTTGVTKRKRDHLLLLTKNMIVHMSEFVQDGLQRFELMWEKSLLDHYKPLAFASVGEGSGYRVDGSESGVVSRVSVLVPVYFRAQGTCLFDRSRESKAASRISGVGISPGDMRVGRLADAYVFGALSILSTSQLALSQVFPELSDDLVRPDRVEVGTAFPKEQQYNEEGVYAVRFWRNNRSRVVVVDDYIPCSQYGKPVFGSFTGSNGKFEIWSLLVEKAYAKLHGGYETIVGGQEGYALQDLYGGVPSRYKLQEKCPNEEVAWQAISSALQAGSLVGCSNEDMAAELPTGLRKTDAYGLMKLVELDYQGEHTRLVQLRNSWGIGTHQQREKWKGSWSNEDPKWHSFSRMQKVECGFQFREDHTYWMDFSAFYCFFTTIIESRNLYNFRSLPDGLGNPVSPSLSIHIVSGEWNGITSGGRDAMHLNPQVQLFSPATNGCNLIVHLEQPSRRMKMETEYTSYIAPVVVKNGERQQRKIDLSQDVVATGTFISNRSCVLELPLMPADGGKPFVIIPATYDGHLPYQLGFSMTLFTSKPTAVVPVSDTGSLPVCCICQKALSGTFRTFTHEQDGQRKVDRVCQGACVDAYRERNTPVCVDCHQRIEVVEGRFSGRMFTLPDGSSVHAECIDAYQIRTSEKCIHCGHAIAAIPGRFDGKYYQVNGGKCHGECMEAYQMATAQRCVHCGEPVIKLAGKFDGRFYKIEGERLVHFECWEQYQQSVAPKCVHCAQPILQIPGRFDGRFYELASGIGKVHFECWNAYQTLANAPK

>PpCys53_PPTG_13332

MRIASSSLLLASLALADALKTPLEYEHEFSAWMKTHSISFSDALEFAKRLENYIANDMYILEHNLENAWTGVKLGHNEFSHMSFDEFKFKMTGFEMPDGYLEQRLASRVDGLWTDVQVPESVDWQDKGGVTPVKNQGMCGSCWAFSTTGAVEGAAYVSSGKLVSLSEQELVDCDHNGDMGCNGGLMDHAFAWIEDHGGICSEDDYEYKAKAQVCRDCEKVVKVTGFQDVNPQDEHALKVAVSQQPVSVAIEADQKAFQFYKSGVFNLTCGTRLDHGVLAVGYGSDNGQKFWKVKNSWGSSWGENGYIRLTREENGPAGQCGIASVPSYPFATLISKDEQTETEKVVEEPRSVPADNPVESFPAEERDFRPMNLADLFSSAKITQCGDVGSAIIDFSDLEVTPSSPQRGQPVSFFGNGNAKRDFASANFKLGVKLAGTQVFGHSGKLCGDTHVPLPLGLGHIDVHGFACPMKKGKFSDLKVDVNLPIIAPAGNYEIQLTSDDDSNSPLFCVNVELDLTSGEGAAKKTHVYEPISYM

>PpCys54_PPTG_13351

MTRRNLQNVVVFSILLQLQSVSRVAAQTTDSLGYPDPRYSTTQSLTAFSCTGGARNCPNGGQYASAGGNLTLTIVEMRNLPDLDSFGLAGLETDAYVEALIGDNVRTSGVVWNSLNPKWPPCTEAGCAGEDDLVRDLNFGFRPAGTEIIVRVWDKDSGFEFGDDLVAQVTLNAIYCSAFSALKQKLPSNDTSTWALAEQPMCVEELWIPLTESGDCTDAKTSAPCMRIRMTAVPFQMRTEEVFVSGALVNGGMGGYFPDEESWLYGRVYSSSDTRLLSYYRMADSQGGLLIRSSSTSNNNKGNTSLISTYGYAPYSRVTFNFAAQLFVFRRVDDEASSPEWLNTTFGWVETREYAQLMNVAGDFKAVAQNFTPHAINKYGDAEGRGVITGANVALNYTDTTLSMYFIVAVPHETLDVVPAVYSKEFSRGIFLEITVQYGPTFAFLMILVVRYLRRMHWRVERVQSFLAEKVANPSGQLSAAAGVQPKVKEKNNTAKGKEKIVKKTKVKKPDIVAQLFFCYGDGENNAQFRRNLCYATWAVNIVIASPVLMLLSWGVTSIVLVTPPAFGFGIVFLGIGALGGIYAASVWVRTGWRMTRRTLYLFAVAFFGAFVFLFSATFADPKVYVGGEDLDFFSLSCIFLTLNMMPIIWLAFTNDSKLSKSLKQVIAVVGASKKVTTLKSKFKNLGTLGLKLATAKSDHQVVDPEESKTKRKRKESPFAAILGDHYTVEQSLPGLEYADILKSVFVTPASTRRRVNRRCYWSVLATLVVYCIVASAWTEYGTQAVGISITLVLVDLCVYMLHRGKSANNWSAGYVVFLLSSVRVCLAVTCGRYWILGHAFLYMVFGTALCREIIGRNLPRMNKQEAGGVTFFGHDPQKERQHQLDVGTTPEFVLGFLSFFYLFLLLGVAFGSDANQTVQVPVLGQEWPLWIFGVLAFIVVLFVGLSLATSRAFFLQKEQLLSDYAMHVYLFVQPLRLPFMLAAASEVLVICSGLFVYASTQSTFILVSSFFAPLILMLSLAVNVQWKKNDYRLVVWPPEDDEEDVLDDDDDGGFLDAEAALEKEAELMRENFVLPPLKGKGNNHVFDSADETFKMPNLPSKSLLGLVGGKAAGVMSPTKKLGLALPGKSKPTPDKPPNSKTQVGVPAVTETGANNTALAIKPAKPSRCAWLRDLMTGKISCRAFFSRKTWSKKYREENNHSPAGKPLLNRNGVANTAQDDDDEEGARPLLDDNAANNAVLARRKTMAQGGDGDIDFAKMTLYQAYQQGFLLPQDHMTIGCFIALLFLIMLYGMILTATENPSWLGQVIWTGAYILIFTLSPTIKFFKTATFTPDMKYCFGGSAVLAWITGFILFFAVLKADVNQVESLVILSILMFYPIFLLFAVTLGRWQDENWVITRLIRRIGGVCMVAIVLWVFEMYVFVSVAFGGVLTFLLALFLFITYYVVKWVENDRYLAPVYQRQASFIVLCATVAAIIAALALGMSFFSCLSIVFVVLLLKYSLQFVAGWIVAKSSADESVFYSPYLFPVFSYNAFTNNVVDETHNVLPLYMVFLLVFLWGVAGVMFFDPLGFGIGLCSLVLLAFAGVTAHLCAVTPVQMGIAAKYVNEMILKDASEAAQAVANRRSQPFTLESPEFVEQERREKKAELEFQAIAYGGGAASAFASRKKGTKAVDIKVEEEIVKVEPRLTCGDIALDIEDVDRQCRYITTTHGLEVCRPDGLLSWKEVKQDIFQRGVGPFGFLYAFTVPPRVFRFLRWKYFPATLKWRRKAKSDTQADRQADTLERGDCRIDTTPADPSRDLVELLLRLPELDAALDRAFYEETRCVIHLQLLMLTASDARLSREKIMFQKFLRENRFKLMSNGISPPANVFRTSSFASIDIPLVAVWLLSLTPESRARFHALKAAFNEEMERTDAIVDAEDRAALVAQKELQEHWAPREAEQCRQRMQEFLARRLRRESEGILTEEEDSKPGYDEGLVNAKEALLEIESGYSCVPGEYGRSLQFVDREFPPDTTSLTGCSHESEVAPQWKVSSAINIASGLFDGGTDPDDVRVGRLDDAWLLSALSIMAASGGVDDGKVDELIDRLFVTKQTSLTGAYALRLYQNCQWETVVVDDYFPVLYDSAGDEAELSVSAGAAFAHSRDFEELWVPLVEKAFAKYYGGYAALERGYVHHALRVLSGCECEEVFLAPAARGALKKTLWQQLKLFRKNQFLLGAASLPSEHADPALRASGLVFDACYVIYDVRDVDGAQLLKLRNPPGDHEEWKGDWSDSSRLWTRRLRKRLGVRKGEDTGEDNTFWMSFDDFCHAFRALYVCRYYDPDKWTVKTLDARFSREDATSSGLPTRHNPGCTGLDNNPHFSLRVSRPTEVIVTVTQVDSRGMAPVAVLPIAVYIVAHQDQKDRARRVTALNQDNVTVHSGVPARNREVRIQCELAARTYTLLVAAYVSGMEGPFRLHVQSNYPVELQQIWPAAWKTGNGTMTPVPHGTLVERMAEKMKHTVTESAAGTKIMAKTHELADKIAAGAAIVDNVMRDEESILEEQLAKQQQEEADEAEKKETRLTGKKKKKTDPDAPQSNPWIEQWDEGAGKPFYFNKQTGMSSWEKPDDF

>PpCys55_PPTG_13374

MVANSQPEAAAVSATNATNTTAGATVTSPQKRDVYLTGFGKFGDILENPTTFLSTKLAEHPKVTESHVLEVSKESCEEELAKMYARAEDRGRPCIFLHFGVSAISRSLKLEQVGYNVADFRIPDERGYVAKDEVIHKGEPDEITTKLPLEDMLKTLQDVHPRVDMSTDPGRYICNYVYYRSLVWVKRQEANGHPEHLALFVHVPEFRNVAFEDQVALASKIVDLVADL

>PpCys56_PPTG_13415

MWCAFERQNVVTLGNNTNNRLEASWEQLKDMVNAFMAVDECIAGILCYQHQQEKSFKNCVYNLSVVYDPKYDREMRHHAKLVSEHACELVYDDYYFAITKAKYKFHEAVPGVFLIQHESDDEDALDESHSEYSLTKSDWSCSCLFMTSRLLPSRHVFFIQKALRCENIIPTHLLNSRWLRISLRSDAEVPQFTGEPFGVSRVLKESTTCWDSSRKFREANYLTSSISEHLSGLGMREYRVAMATLREVDARFRHGEYDIITRTDSGSRELTISGELGTASGGLGSGSTELDSCLSDVRSDGIVVGTGISEMGASCDELVSNNAALVTEQHFTDEVGSRVEEQASSGNFNTQVPTLSVGGLKSEFEILSPPRSKGRPKQKSRAVKAKRKKAVAMVQEDMAMHERQRNLATIREILKGAPTYESSHECILQFKAYHFDPKPKPPIAHRTALLPPTKPVMMPKEIVRVFPMDLLNKYRAKVTAYQRKNPGTPETDIALEILGVGIFENSMLALMRQWHATTKTLKRIQRALTWIQQLDFYRHTNASFNVEKDPDLPNKLKNISILAVEKTEVLELAAKECLSDSVMQMVLRKQFGADPNVKVVDPAYLGIYNGEITTETSHFSRVLTGVTKDTKALFPINCNNNHWCAVLLDFQDGKSYIYDSMASSYLNTVRAAAQKLIMMIPGKHRPSRRMTIYDPGLGVPSDSYNCGVYVLLAFELLCGAGPLGHVNKNILQLLRYRYMRMLIEV

>PpCys57_PPTG_14714

MRGNAKVGATRDQLLALVQRRGAAHRALTLFTPQAAALVRNVSATNSSSTTLSLLKAAVERRVPDWTQSLTQERDAKRQRVSSSSKTDVVFAAKMAQSLVKVYAVTSGAVAKEEELDDLVAAMDLACVALYVVCALEHAVRLGDLVVDNLLYQIAKKYADIPKMKEAAACMTACVHARLWQAHLKLSNGASEKSQLMRLLDWQDENDDALASEMMGKAELAAEFPTPKSFHTAQFARLLLGHTNICVSLLSAVKKYEVVLQVAETTLSPWISHLRRSSEQDAELASSFSDRTFRVLWKIAAAVNGAKPKPDTTSGGLRFRFTALSFLLKCSNYSSSYFVQQVHRVGVQHERATKRSQQGLKELYWFYDQSANALSDSHPCSTHIHRSDSLQWEYIQWLEHFALICELSGMHLRSAMILENAVQYVQLFGSAGKAIETCLVVSTAGSLFSAASSENRGSCFISRDGFSSSAIDKNLETGTGRMIRDLVHNISSDKWTDQCEKAARVYLKKLEALAPYSFASMNSKDHPGFVPFMTRSLKRSSTKMFNYAIAVQHSAKYQLYYQVITSFDEICKAVKEVWSSVANSDAKLMETLILEQSRLHRMDVILSVRCCLADNSATTKPALRMLSRHVTSVRELLDNAIMDGKLSDSIINGVLGDLNSIARECYSCATRCYKVGNYQDTISALLGAFEVVESYLEYVMCSNLDVEGIHEAHTQLKADAIASLLAHCYRELGNCTKSRLFTGYSILYCGDIHEIIPRAIVDKYVSCILEEAKFMEEEEQPSIINEFKIFLDNITHVFKSRQISEHQIVTLWREFRDAFDRASATIVVSLRGKSCALSGDVDMSSVVRVQMCCELEHYLTVKLVKKKMKAINGEFPISILLDVSQSLADRKLIYCCYYAKRNIRDTIEGLRLVHHSLAEATENLLNFCDAVGVDLGGIYGWCGVVTMEMALLVSYGGSSDEYVASGNISVSEESAIADIERCLAYWDGEDSCGFLFDGLYAVQCLESVYNTLSLISCSPLEQAARKLLQTLHMSENAQGLMSSVIPPPIGLMVMDSREMVLTVEEGAATAGDNREMQLFEQVDRDLFAAKTSHLGAIRDQACQHLLSAMTTLGDIKQNVKYCQTATVAKGIGMRELFVHTILSDIHFYEGRSKDAITEAKSALQVCWKMAKKFTAPSSHTEAACFELPREISAAEYLQRKSAHFLYFMALECSSWDLLLAAKVSLCRIASLYSLADQPHRAASYLTEAMRLVGGLNLRFFRRGPFYEYAELELNANHLEKAKVAMSLLSLNPKPNQQSKTNHSIADEHRSTIQFDPAIIEQKCDEIVQQGDVHLSEAKHQEALACYAKAIEIVDVTEDCQSSISKRLGRVKARCWRKYTRLQSQTSDFSNVSAVKSLLVSMKKLKRSLKSCDVHLERVKSMLELGRINTRLLRSASPRAFTSLGRTLSLLEGAYLLGDHLGIPHLNQELRTALGMAYFAEIEENTRDDASSMDGGARAAFLSWASCALLANAGSDDAISIAMDNDTSTQDGLVVQLEQLAAGSNAFQKQEPQEYLTRMAQGIAEKVQHLPDNWVIVSLTIGLSSELVVTRIPTNGSTPISFCLPTVSWSKCIREMDAIIQNSRQGLSGHTAEETSSWNDEQKRKWWDNRKLLDRRIEKVVISMQKKLGFWRCLLVGGSTFESQIVRQCWELLTQTKTGPIKLAERNQTLLCAISNAQQYLPDSEVIDGLKHIAAEIEVPLSDSVSREVLQVLRTNQNDFVTNSTEATHSNLTNLSTDKIARMKVGEVKQLLAAEGLNIDGLKKELVKRLIAARDAALVDRIRTSTGCCDGGNTADVSTILILNHQLQQFPWEGMDVMDLCSGVTRMPSLDLIMQNAKLLSPVRRDRVRYLLNPAGDLKSTQNQLGPILEGGATTYGWEGIVGEIPDPDKLRNYLLAADLYIYCGHGSGEAYLHRDKVLSLKSNCSAALLFGCSSGRLEREGIFGPSGAVLAYVRAGSPAVLAMLWDVTDRDIDQLSVKVLQEWLLSDNKDDSRSLAQVLQSSRGVCKLKYLNGHAAVCYGLPLYVAKS

>PpCys58_PPTG_14795

MRVQALIALSTAVASASAARTDRSLGAFVDCPSVRSDGSPCLWAGQNGEIVDSKTLRDLLVQRNLVSFSERDGLTRNLQAHMTYIEDVHMYAKSVGHEFSYHMGVNERHLTSSPKRRLSPQGLVDQEVKSAHSRRLKAEASGSTASTTSTVSSSGSSEYWNWCDTDNSFGYSVCSSVKSQQNCGSCWAFAAADAIETAVVIAENASAAVSLSPQQFLTCSTLETTQTFDYCWASDSGVDGASWMETEIKWESQNNGCNGGMTHGAFIDAAQNGWGLVTELTMPYDDSSSGSTSSNNVSSTCTVSDNETAASITGWEQVVGTDCTASNNCTTLLRTALEKQPIAVAINSEDPFGEYAGGFYSCPNDGDLSSKDDVNHALVLVGYGTDASVGDYWILKNSYGSSWGASGFMKLVADSKVNCGLNIFPVIPTGASAGAAATTSVDSGGDKVFVGLSPTNWIVVAAVTTIFTIVMTAIGMIISQRKLKSIRKQNSVMYAGRAPVNAQQTMAH

>PpCys59_PPTG_14796

MQIRAITFVLSTALVNGAAVQTTGRSQGTLVDCPTVRSAESPCLWAGENGQVVDSRSLRELFIERNYVAYSDRESYERNLQEHMTYIEDVNMYAKEVGHEFSYHMGVNERHLTSSSNRKLTPEQFVDQELISAHSRRLQEANTTNSSTISSSGSSEYWNWCDKDNSVGHSVCSPVKSQKSCGSCWSFVAADAIETAVVIAENASAAVSLSPQQFLTCSTLQTTQTFEYCWASDSGVNGASWMLTEIKWESQNDGCNGGMTHGAFMDAAQNGWGLVTELTMPYDDSNSGSSSATNSSASCTVSADQAAASITGWEQIVGGDCTASSNCTLLLRSALEKQPIAVAITSNGGFGEYAGGFYNCPNNGEIASKNDLNHALLLVGYGTDSTVGDYWILKNSYGSSWGDSGFMKLVADSNINCGLNVFPVIPTGAKAGAQASTAVDSGGDKIFVGLSPTGWIGVAAATTIFTLVTTAIGIGVSQRKLKVIRKQNSAMYAAQTPTNAQNGY

>PpCys60_PPTG_14797

MVPAPWLTLLALTAAAAAPSMTQAQDPSTFGTLLSCDDARCLWADRDGIAVPSDTMVTQFLQDEGMDAGRSEFRRRMEARVDYLEQVQKHAADRDWAFSYTMGVNSRHLYHDGSRMLSPADFVQQEHRAAQRQQQRRLTEQRRLTNSTLDVRETLDWCSTDNPQNQSICTDVKSQNQCGSCWAFAAADAIETAVVVNTGTSPRSLSPQQFLECSSREMTATFDYCWADEGVDGSSWLLTKMIWGSRNNACNGGMTHAAFADAAQLHWSLLSQLDLPYNEEDTSQASAATLANACDNSSTDNAAASIAGWEQVAGPSCDESSDSTELLKLALQQQPISVAINSGGSFDAYKGGIYTCPNDGDFASSGDINHAIVLVGYGSDGTTDYWILKNSYGASWGEKGFLRLAMDSKINCGLSVFAVIPTGAIAGAAHTAVDGGGAVEFVGMSPDSWIVCGIAVAVVTLFLTVIGVIYASRQRNAFKETL

>PpCys61_PPTG_14804

MPPRLRNAQKRPLSADDIDVDNDSNASDGSSSEWQPSPSPPRVEMTGTTSSASEESGQICVIAPPPGETQFRSWEEFEIYLAKYQAQLFQIFRVRTNTSVAVRNGRIDAQGSSASKHSAEWENYAKTYVCTHYGDSGLGMLCPAMAYLKYVARRFKNGEYNISSSYLPPEEQQSELTLPPTQLERDQPHGAGADVVSENAPVDSNSLSVGTGSQSVEPVSQSGESGTQSGESGTQSGESGTQSGESGTQSGFEPQIALATLPQANDVFEVASPPRSRGRPKQAARSKKEARRKNVQTVQDDSNLYAANLSLANVQDAVEGEPTYKESAAILSRFKLFEFSKKPRAPVAHNKLSLPPAKNITPNLAEQDIAVELIGIGTYATETLGVMKKWHRAMKSIQYVEKAISWIETIDFTLPMPQAFRAESDPDMIPKLKAIPLLSVQAEALELVGKSTLGDGTVNTLMLKLFAERVDTIGIDTSIAGNVMNGFLPVDTMKSVLLGATGEKILIPVICGKNHWCSIMIDLTCKDVLIYDPMNSSYGSKVRPLADKLVTMLPDFAPRKYRVRLYLSELGVQVDSYNCGMYMLLAFEVFAGANTLSLLSRKELQYLRYRYLGMCI

>PpCys62_PPTG_14921

MHVASEGHEFQILPGISVLERWSTFEALDVKDELTSAANALQPIVNMSKLKLPKRIRLPDSSDQSAEERKSHTPSDDKREVVYIRLRRGECAKQVVLTSAEKYSYAKAMLEPLLKHLSELSSADFYQELKAWKETVEAGLSRREAATANTRDIGDNEDDEAEAELYSFLNPPSDLSTTNLMNALQNTEGVTDGSSDAEHGGLEQADTPTPPGSDVPARQVDIINVPKPKTKGKTRSNPKQLRQTKIATRLAVHKYPSDLTVQLYEFVIWARNTSSMILVAEMVNKYLVQLDDAYLRARTIECGWEAMRPAAYMHPFVIPGPLELNCGNQTHLIIPSRSRHPARSRGVYRSQPVEYVGAATTFYYVKTKVRQWFEDRKWLEQDWRKIVSDVDFLAVETGTSGLSSDAVRARHWAMANEVISKFASCRLSAEFVTPSRGCFITFENVVGALCKGWLNDSPIDFCLEVIGSTAEKCHVLSSHTTSTGRPKTPKKLITDTKFIIQPVNLKRSHGGVVITTLHYLESADILRVHPYLYEPLIDEEYHEDMEEVWKGIKDQENKVVMEGLRGFVKRWCQASTPTTKLRIDPIQWVEVPQQPDYASCGVFVVAQAFSYVHGNLQWQHNNISKTDVQVMRLRMLWLILCKSRESPMARGKVERMKKIQDQLLKELK

>PpCys63_PPTG_15428

MKKKKLIPINYINPRWLLGSDANGPEDDESNDDMSCPGFRIHNSNMRSTEHAVLNGSTKWKTAMEIAERVVEVMSSHGTQTFLEMANSLRRFGECASEGVVPIIGNYEQSEHHTPQNSAVVDSVFEHDSSDAESVVPPTLQTVELRENDGDHVEDTTKSRDESSDDVTHERIEIETTDTDILLAETDRILRQLDDVVSSDSDDLRAEGINRSVNDSNPLVSSTNELVNSNNELVTTTNELVNSNNELVTTTNELVNNIEHGNELMNNGNELENNGNNKKKRERQNASEKDQTKKGVLNGKLIRRKRQTISISSGEDEASEDGDQVAGLDITPGSFRVSASVKSKGRPKIRRKQKREAKRIRMSEAIAEANALVQGTLVPVKDLALVRKTIVCSLNVTDALPVLASIEEVGSPSWSATSIVQLARKQKVQQKVTIVFPKNYLSKCIAGINTYRKSLPSEHDAGKMGVSIRKLGTFAEKDLLTMRDWHNETPKLEAVRDLCSWIRASKFSRIALSTPLKNCATVDLKACATRLEAIDVNKAISNRFSKGVMHELAYFRRSEWLDDGCLRVVMSHLMDQDLDNNGQSRIGGVNPLYARVHESMKKEVITSSPFNTSNRLVLIPIYIDGHWAGAVFDYENSKAIIFEPMQTSKYYKGVCEVLDEYFGEYATELEPIQQRAPRQEDTNSCGPLVLLFFECMIRRVPVPNIPSEHIAYLRFRYFFLSTKGAFCRSPGLRE

>PpCys64_PPTG_15736

MSWCTIESDPGVFTALIEDIGVKGVQVEELYTLDEQQFADLSPVYGLVFLFKYESNHGEDAEPPVFATEDDGIFFAKQVISNACATQAILSILLNAQDVELGETLSEFKAFTSDFPSDLKGLAISNSDKIRLAHNSFARAEPFVVEERKATEDDDVYHFVAYVPVNGKVYELDGLREGPICLGDVPDAENRDSWLQVACPVIQKRIEKYSATEIRFNLLALVKNRIQTYEEQLQAIIEAGGSEQQAAQIQADLAAEQQKRENWALENKRRKHNYIPFIIQLLKTLAEKKQLEPLIKQQLDARNATAANTTNAQ

>PpCys65_PPTG_16055

MVLLLLLLMVLPLARSCTMIAVGRNATVDGSTLVAHTDDAGFGAADLRMVRVPAQDHEEGATRHVYNVQPGYPRLVSSQRGHEYEPKNDQEQLMTPLGEIPQVRHTYGYFDQDYGFMNEAQLSIGESTSGARTVGWPMDAGPHGYNMFGIGELTKVALERCDSARCAIQTMGDLAVKYGFYSEDSGDPAKPEYVSAAETLGIADRYGEIWVFHVLTGPQNASAVWAAQRVLDTHMVVVANGFVIREMDLDDPDRFMASGNVLSFAEEMGWWDPKQGKFDFTAAYASPTPDPVRALYMGRRIWRVFDLVAPSLQLDSRLGHYPEFPTYPFSVEPDRKLDVADVTRLLRDYFQGTQYDLSKGLAAGPFGNPMRWGGDEKGVVGGWERPISMYRTVFSFVLQARAHLPDEVGGVAWYGQSSPHASVYVPFSCAQQEIPAAYVLGKESEFAHGSAWWAFAFVNNWSMLRFDAISRDIRARIAELQKTCYTERKAMEHHVTHTKSLTASDVRTYLQEQSNRLASRVIDEWWTFAWKLVGKYADGYITTGEAPEDMKMPGYPEWWLKLSEFSKWPGDTLVPPHIPKLEKQLELFESRQNASANLGVQPSAGDDSASPSSTISIAQLGIGAVVGAVVGASATWFAVQRQQRSRYLPIP

>PpCys66_PPTG_16569

MCPPVDTNAECWVITVASVGDFTHQQLLPVQYVWSLVDASEKGMNCYSWLMSIADEQICYEETAAVAKRMLDAWLKERLPGFGEGVDIEWAHLYCARNGCWYNDNLITAYGKMVEGVYGNNTTILLPPMKKPVPKTPKKGMRVPPTTLSLITAASSGRIFLPLNINGTHWTCIVVDGTTQTVCCYDSTDKRANHNLLAQLADEIVKKSLTKAFCVTVVHSPIQKDDFNCGVFICLYFWRRFWKGAGSDYTEKGLLRRRWDILRTIMKFSDEIKEKEKVTE

>PpCys67_PPTG_16596

MQPRVSTATVALAVAFVAAIISDNVKARPMHAEIVPKYHRYLADKETIKAELNEWLKTYSDSGPKHGYIPVTESRSSDDDLEDKLQRFYLTKEQIEEARELNPMAEFSTDGPFTLMTMDEFKQFLSNSHLNETGKTEPPAVEKPKTLSEKEKKTRPSEEVKNKEDNDTKQSKSDDKNDDEEKKTWAPATEGPVVVPMVRRLRNAGKSQYAFNGGDYSNTGEGFQSTSDTYVKSSQETYESQSAPQPNTNNGGNTNQGWNFGDVSTGDSAQIAIVSGGTGSGNNGNWWGTNGWGTTWGGNTGNTWSWNQQWTQPTGSNNQWTPPSNNQWTPAPNNQWTPPPNNQWTPPPNNQPPSANNPPAPAPVTPQTPPTLAPAPPPAPPAPAPPAPAPPAPAPPAPATKRPATKRPATKAPPTKAPATKAPAPAPAPAPVTKQKATTTASTSDTARDSDSDSVDWSKSGCVNPPGLQGQCGSCWAFASLGALEAAQCIANGDKKAPSYSEQHLVSCDTKDFGCNGGAPVYAMQYLRDNGVCTESSYPYTSVEGGTAAACVKTCTPVKSGITDIAQLKSGDESALLGALKKQPVVVSVISNNPMWKQYKSGVITSCNTATVDHAVLAVGYDATTIKIKNSWGTEWGEDGYVRISRSSQGMGTCAVLTDMSYPKV

>PpCys68_PPTG_16597

MPSQDRSTTVSVLRFIGGVAIAAVAVSGVDGRQMHAGLDYHRYLAERAETRQELADWKANFGEMAKNNGWMPPPTSNSEERSADDDEEDHLQRFYMTKQNISAIQALNPNANFSVNTPFTLLTNDEFAAYVGKAYRTYNGSSVPTNRRLRSWHRSSSTKTTYNSGTPGAISKITSTSTSSGTNSNTQASVLGGTVNGGTNYETTSVKTITTTGADGKSTTTTTTTTSSGPGTESTTVTTSSGSASTMSNFGFGSGFSSLWQQWGNGFNFGGMGRNDFHPETVKPAGSASSSPTTNAPSTPTPITAAPTPTPTQTTATPSATAPTKSFTTTVTATDTSTASTSNEVDWTDSSCMSPIQNQGSCGDCWAFSTAAAIESGQCINGGQKTLNKYSEQQLTSCDSQNYGCSGGAPIYAMEYVQQNGLCTEDDYPFTSSDGTAASCSNGCSAVDTGITGYETVDDASGLATAVAQQPVIIAVASGNNAWKQYTGGVISSCDTSELDHAVVVVGYTDSEWKIRNSWGDSWGEEGYIRLERTSDSTGTCGMYGDMSYPIF

>PpCys69_PPTG_16598

MKPITLITTLVFATSMLAVVPVNARPMHAGINYQRYLTEIDTIKAETDEWKAMFEKTCKENNWMPEYSTEERSSVDQDEDLRQRIFMSKQDVLEAQAGNPNANFSIMTPFSALTKEEFAAKVLNSYVRGNTTRQTPTPSPAPPKRSLRQQETYTFTSMQDMINSLMQSLQQQMGGSWSIATVKPATDNTDTSTAKQWHWTIPATFPTVAPTPVAPAPAPNTPPPAPVTPAPAPVTPAPTPAPTPAPTPAPTPAPTPAPIPVTAKPKTAAPRTYAPVTPAPVVEPLTKKPSTPVRTEKAVLNSANSVDWSATKCMSPVQSQGQCGSCWAFASVAAVESLQCIKNGQSEINKYSEQQLVGCDSQNMGCGGGAPVYAYEYIQKNGLCSESAYPYTSSNGGGVSCSASCSKSQTGITGYERINEGDEAGLVDALKSQPVVVAVASGNAAWKQYTGGVMTTCETTQVDHAVLVVGYDDNTFKVRNSWGENWGEAGYVRMARSSSGMGTCGMLTDMSRPKM

>PpCys70_PPTG_16600

MHVPTLCLVIAFTILTIPTIARPMHSGIDYQRYLEEIDTTQADLDEWRSKFGDVAQKNGWMPVSEARSADDQEEDLRQRIFLTKQSIAKVQAANPSANFSIMSPFSAMTDEEFNKYVLNSYVRGNSTQSTNRTNSRQLRSAGNSDSNIDAGSDNASIIKTFESLIKTFFNGLNTATIQPTSDSSANVKRSGSQASYKFSDFWDWRPLQPTTNAPATTSSPTVAEASTDSVDWSTSKCMAPIQSQGSCGSCWAFATVSAVESAQCIASGKQSLTKYSEQQLVSCNTQNWGCDGGSPVYAFDYVQQNGLCKEDSYPYTSKGGYADSCSRSCDAQDTGLTGYSQVTGEDELLNALDKHPVIVAVASGNNVWKQYTGGVVSSCDSWQPDHAVVAVGYDSSSLKIRNSWGTYWGEEGYIRLARSSSREGTCGVMMDMSTPQM

>PpCys71_PPTG_16885

MPPRLRNAQKRPLSADDIDVDNDSNASDGSSSEWQPSPSPPRVEMTGTTSSASEESGQICVIAPPPGETQFRSWEELEIYLAKYQAQSFQIFRVRTNTSVAVRNGRIDAQGSSASKHSAEWENYAKTYVCTHYGDSGLGMLCPEGEPTYKESAAILSRFKLFEFSKKPRAPVAHNKLSLPPAKNITPVRSITRIFPKDLIERCNAKVSTLQKKHRDSSDQNLAEQDIAVELIGIGTYATETLGVMKKWHRAMKSIQYVDKVISWIETIDFTLPMPQAFRAESDPDMIPKLKTIPLLSVQAEALELVGKSPLGDGTVNTLMLKLFAERVDTIGIDTSIAGNVMNGFLPVDTMKSVLLGATGEKILIPVICGKNHWCSIMIDLTCKDVLIYDPTNSSYGSKVRPLADKLVTMLPDFAPRKYRVRLYLSELGVQVDSYNCGMYMLLAFEVFAGANTLSLLSRKELQYLRYRYLCMCI

>PpCys72_PPTG_16951

MRAVAVSLSRFKNNQTVMLPPPTSDGQRGDILQEKAIRSIARAVASRPFVLMLVNLGGVHWAGIVVDRDDKKVITYDSLNGTKNRKKLKKLAEDIMKKALQGETYNEVDVTKPKQKDGNNCEVFVSLFFWRCVSAEAPSDVVLSDLSPSGITKLRWALLRAILKMKQR

>PpCys73_PPTG_17193

MVTLNFSNFHWCCVVIKVKEKRIYFYDPLNQGPYMNAAVDVATNLKISGLQDYDVIPQNNPLQFDGYSYGVYVCWMFIHKVVPGSPVDTSDKSLTRRRFNLFFYLLTSRLLPFEGPLQQTQLTQATRRKTRRPRAATRRFHRRRSRIKSISKAAAYALASLI

>PpCys74_PPTG_17719

MVQEDLDMHENQLNLVSVYEILEDAPTFTSSHVRLLQFKLFRFLSKVKPPIAHEISKLPATKPLIRPDEICRIFPMELIKKCMAKVTAYQRKHPRVQELDIAFEIVGIGVFANSTVALMKRWHTAREILKKVERTLMWINQLDFSRLANNASFNATADPNLPEKLKNIPIFSSQNHLLDLAAKECLTEDTLHLVLSKKFGGDPNVVVFNAATLGAVVNGKIETDKEVIRNALSGLSKETILFPMNCNGNHWCSVMMDLNKGKVLVYDSSSSSYLCSVRAVAQTLITLLPERAQPSVRVGTCESGLGVQVDSYNCGVYVLLAFEIFCGAEPLGHVDKKTLQCLRYRYLRMCMET

>PpCys75_PPTG_17817

MFTVPSEILRYPRYDTKAIQDHPVWSKLKMAAASMDLSRDSSVNLFFFGVIHYDWNHWCAVGVNFKTSEVVIYDPQNTGDRFDAIKGFLKTQVLPLLPAPESPKRFRFTRIDLAPQLDDYNCGVFVLLFIELQLYGVQVTGLDSEIRSREAMEFFHYRYLSLILASM

>PpCys76_PPTG_18014

MVHVPPIDAEHESWLAFEKNLREYMKRTRQVLVVKEVMNVSRRNNILKKQVKYQGLQDSDIPLVPLLAQVTRREDGTWGICIKREVYSHNHGVSAEIYSNYPGIRQVSASSPLMPGIELLMESGASSSSIYDYIHQNSTHQVTPADVRNLIARLKKNGAQLSDDDAIAEAVVDFNMESIRNVSTVHQSGRGKTGVISVTSGHMFLERSYTNITTVLLSVATSTTSEGVANVDDKAMEEIRSYVEHDSVKFLFLPVNFNSSHWACLAVDKESKTTYIYDSMNGRKTGKALMQLVTKVKCSLQGSYAHQIAKAPRQKDDNNCGVFVCLFFWKMVSSDAPTDLSPTGLTMLRWKILMAVMNISPQPKRRLRG

>PpCys77_PPTG_18174

MQGFIPAPLLRSLRRFATTNEDEEGDNTALYSALTRGEILGTQRGVKSSTDDALVTRQVAVWGLLRCSPDDTDGHSFVRNLPGGISRIGHFVVVRGEASENEKQLESVALQVAGDANEAIVLVYGLATQTPCMFHCKEGKVVKVEMEVVHSLHARDFLRVIGFAPLRCAVDLHALGDKRALLHQLEKYKVATRDDGDFYVRAGVTQGKRGRSFRVLNGRGEDVVSSDAKTVLESILPEVDDSDGVGETKSKKRNGKKQKKAGKKTTTKQNGDEEDKLGFMPSLEYGDIANLDLLVSLAPLEATADVAAPVVTIPAPSAPNKLRQRFHAHCDALVMVPMDSPIESALTLLRQRLHDQLTEVFNLLKDAPEAVTSVTAHQFPLIGAAFPLTVVSSATDPDFNDESMNDVKTLEHLHRAFLQPLDQPLFRVSRGCSLAQQGEWLASSDVLYNVHEGIPSSGVGERCHSALVDGFYGYYHYLQQGMNDKGWGCAYRSLQTLASWLFVQHYTQQRFLSHEQIQSALVKIGDKPARFQGSTEWIGSLEVGYVLDELFGVTFRSLSVSSGAQLPDVARELLYHFETQGTPVMMGGGQLAFTLLGVDYDPDAGVCAFLTLDPHYSGDEDLATIQHQTVALEGYKAVPCSWRKTTTFAKNSFYNLCLPQRPSVGV

> PpCys78_PPTG_18745

MRILRVFLLFCALHDAAAVPMQVVEDFELRLHHAQTRTRRRALLGPTRDSEATRAEALADTERVVDEGNAEHAAGKKSYFMGYNGLSDLTDEQYRAFLTSRPDRDSPRRKQRKKQPTMNRRKRGKQVTISFDLDDGSSESDDDEEDIEVPTELDWTTKEGGKYMTPIKNQGTCGSCWAFAGVAAVESRYAIENDVQASPLSVEQVLSCSADLDHIRSKFEDNMTSSSEGCAGGMPFLTYAYLQLAQPHGISCGSAYPYVMATNETHPQCSSSLTADVAVTWKSSVSDYKVVAASEKALLRAVTSGPVTANIDATGDGFRHYAGGIYDAKDCLSDGDEVNHAVVVVGFGETDAGEKFWIIRNTWGTMWGEDGYMRIARGSGVGNYGPCNLYVYADYPVNLTAGSNVTSGEPSCPVPVLKFESLSLMKLIGLSGNQIAMLVLCIVLTVIAGVALYHGTEFIQNRKEAAGQLRYQDSYGRWILPSRDQIAAALAQRNSQRPTTGQ

>PpCys79_PPTG_18900

MWIHCCFIWVFRCSIRRTVAKMSDDGKHKRWFPLESNPEVMNSYVEKMGFPTSQFSFCDVLSTEEWALAMVPTPVVGVIMLFPIKPHTEEADKQEAARIEKDGQAVSPNVYYMRQTVGNACGTVGILHAIGNMRHLVQLTPGSYLDKFFNKTKTKSPEEIAQCLEEDDELEETHGSAAEAGQSEQLESVDDPINTHFVCFSCVDGNLYELDGRKKRPINHGPSSPDTVLQDACQVIKKFMARDESEVRFTILALAKTPTE

>PpCys80_PPTG_19421

MSLAWLRNHARNRDRGTTRTRSNSQRLAAQRAHRQFAGAEAYAPRITYDNSFVVDSSVTSRDTNWVSDDQYKRQYLRLGWEVAPIRKDGNCLFRALSDQLYGHERRHLELRRRLVDFIDLERAFFEPFVAGEGVVEYCARLREAGAWGGHPELVAASRLLGVNIIVHTGPVKRLRIDTDKYARTKEGKGKTINLLLKHDHYSSLRKREKPLDQQHGCSDMCLCRRIVTPSRASLDSRASLGARPSDLGSSFIDRNSDVTRRSSLGARPADISIEQRGRLKGAYGRKSVPDAPDSANKKQQKAPSKPKSRPPPPPVKPKTAAPTAPSRPKVPAPAPPSKPKSPAPAPPKAATPAPKSPVHSKAASPRRPRSPMPTPPAKQKSPGVVLFQPESLKPVVKPKAPLVVLFEPEQTPTSKPTPKKSTVNTATPAKPTSAVVLDAEVEEPESNEDVPQRGVRLPRRAVFNGGKRRRSSAAALSLLQEVSASLNVSAPEVTIEPVPVVKPVVKTITPVTMAKPVVKTIKPVTMTKPVIKTVKPATVDKPNPPAPRSMEIKKSSKKVFRQGRPVPTAC
