# Supplementary material for: Two Phytophthora parasitica cysteine protease genes, PpCys44 and PpCys45, trigger cell death in various Nicotiana spp. and act as virulence factors
Source: Mol Plant Pathol. 2020 Feb 19;21(4):541–54. doi: 10.1111/mpp.12915 (PMC7060141; doi:10.1111/mpp.12915)
Supplement: Supplementary file 2 [file MPP-21-541-s002.docx]

Supporting information S2. Basic information about cysteine proteases from *P. parasitica*

| Family | No. | Gene ID | |
| --- | --- | --- | --- |
|  |  | With signal peptide | Without signal peptide |
| C1 | 20 | PpCys10, PpCys17, PpCys19, PpCys39, PpCys43, PpCys44, PpCys45, PpCys53, PpCys58, PpCys59, PpCys60, PpCys67, PpCys69, PpCys70, PpCys78 | PpCys29, PpCys40, PpCys41, PpCys49, PpCys68 |
| C2 | 7 | PpCys54 | PpCys15, PpCys18, PpCys25, PpCys26, PpCys36, PpCys52 |
| C12 | 2 | PpCys79 | PpCys64 |
| C13 | 2 | PpCys20, PpCys35 | None |
| C15 | 1 | None | PpCys55 |
| C26 | 1 | PpCys12 | None |
| C48 | 35 | None | PpCys27, PpCys01, PpCys02, PpCys03, PpCys04, PpCys05, PpCys08, PpCys09, PpCys11, PpCys14, PpCys16, PpCys21, PpCys22, PpCys23, PpCys28, PpCys30, PpCys31, PpCys32, PpCys38, PpCys42, PpCys46, PpCys47, PpCys48, PpCys50, PpCys56, PpCys61, PpCys62, PpCys63, PpCys66, PpCys71, PpCys72, PpCys73, PpCys74, PpCys75, PpCys76 |
| C50 | 1 | None | PpCys57 |
| C54 | 1 | None | PpCys51 |
| C69 | 2 | PpCys24，PpCys65 | None |
| C78 | 1 | None | PpCys77 |
| OTU-like | 7 | None | PpCys06, PpCys07, PpCys13, PpCys33, PpCys34, PpCys37, PpCys80 |
