# Supplementary figures and images for: Two Phytophthora parasitica cysteine protease genes, PpCys44 and PpCys45, trigger cell death in various Nicotiana spp. and act as virulence factors
Source: Mol Plant Pathol. 2020 Feb 19;21(4):541–54. doi: 10.1111/mpp.12915 (PMC7060141; doi:10.1111/mpp.12915)

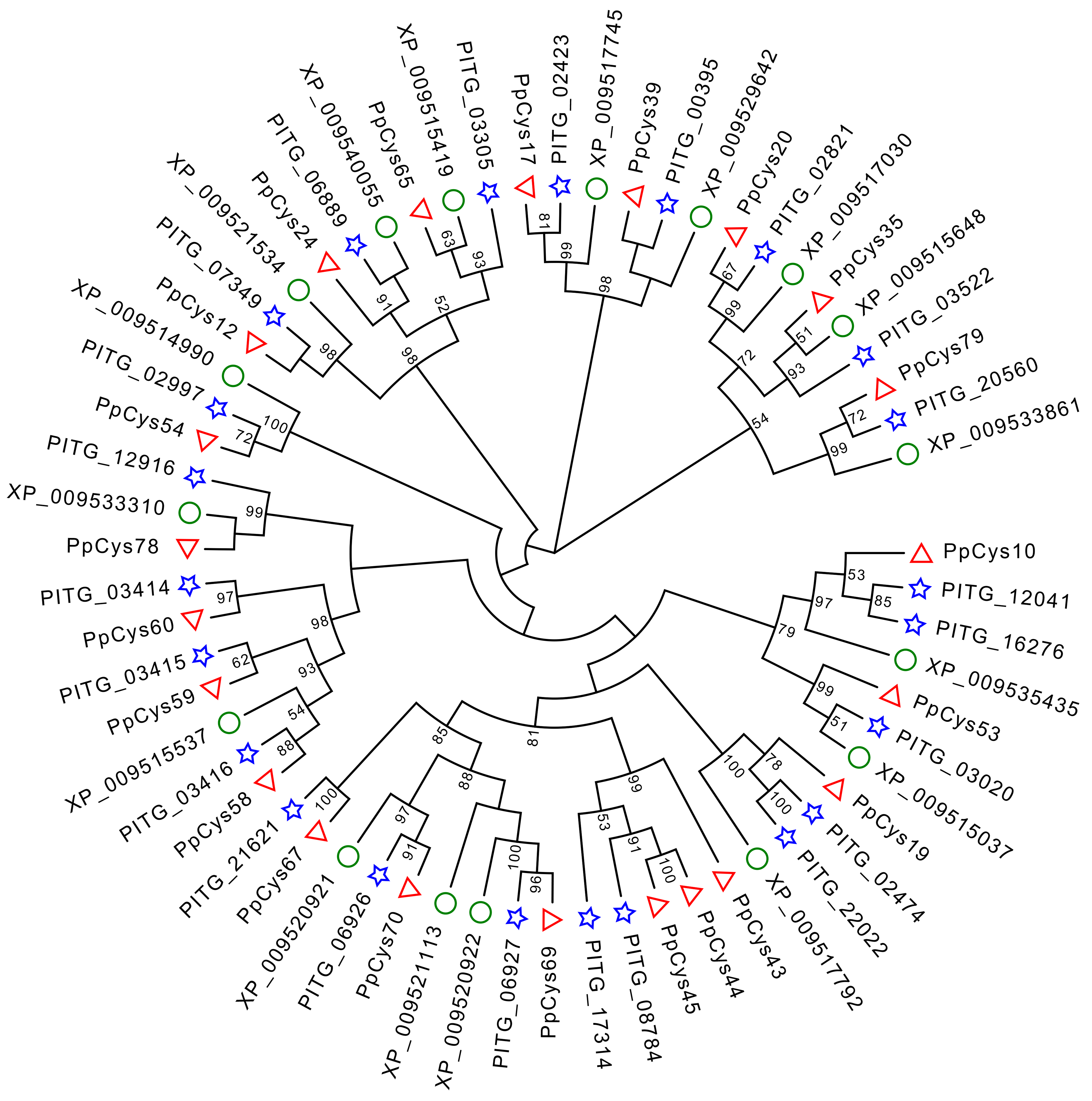

Supplement: Supplementary file 3 [file MPP-21-541-s003.tif]

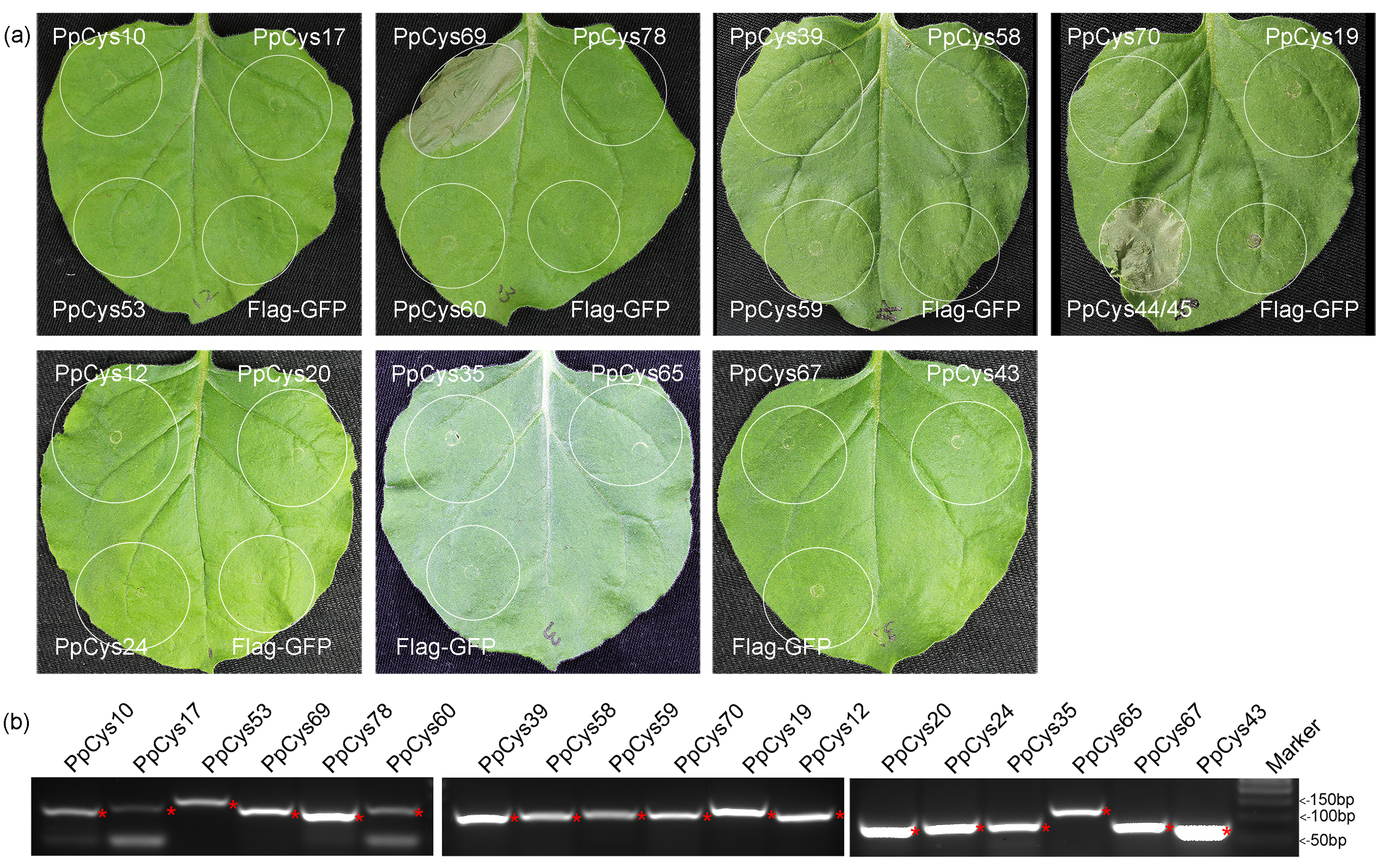

Supplement: Supplementary file 4 [file MPP-21-541-s004.tif]

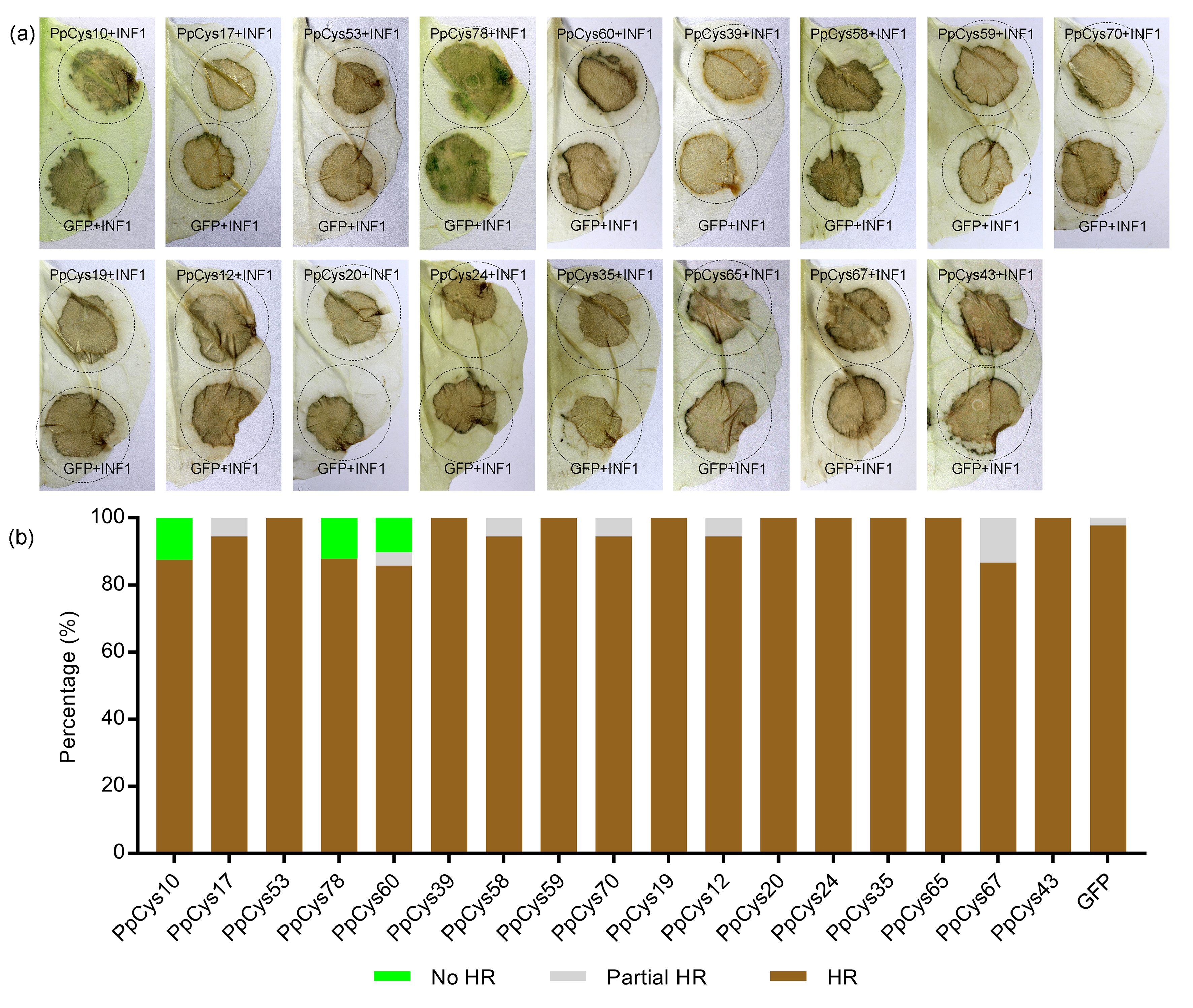

Supplement: Supplementary file 5 [file MPP-21-541-s005.tif]

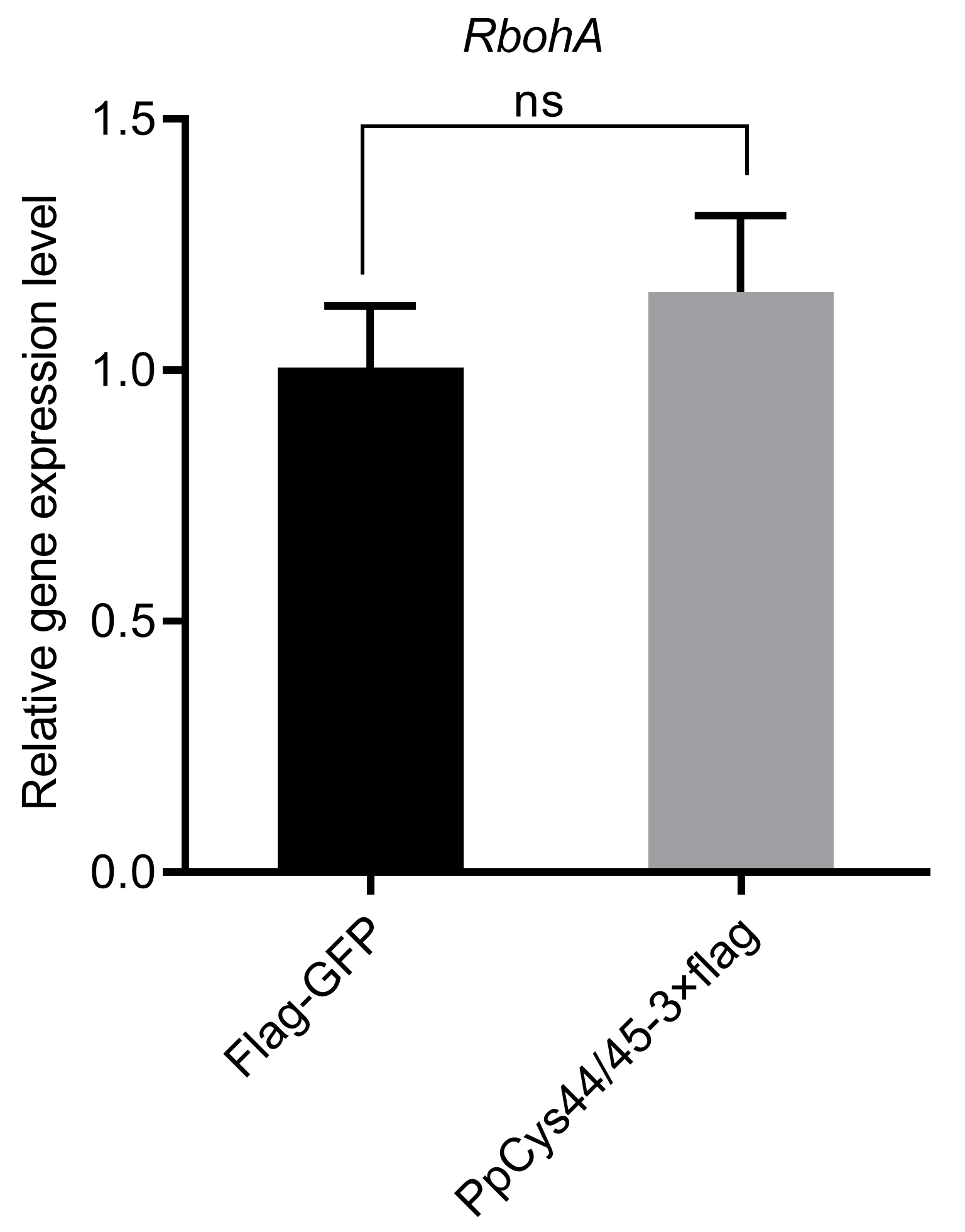

Supplement: Supplementary file 6 [file MPP-21-541-s006.tif]

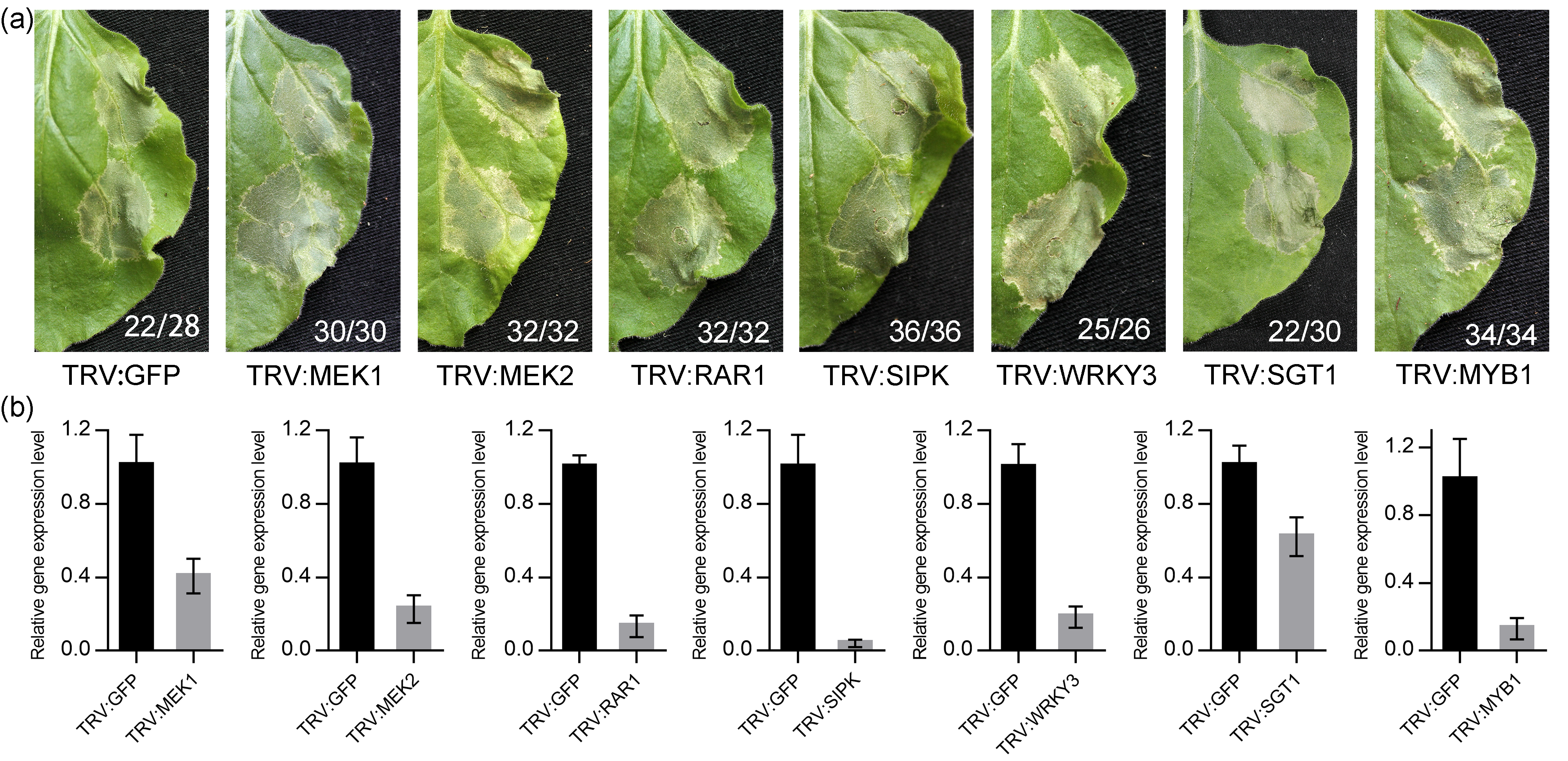

Supplement: Supplementary file 7 [file MPP-21-541-s007.tif]

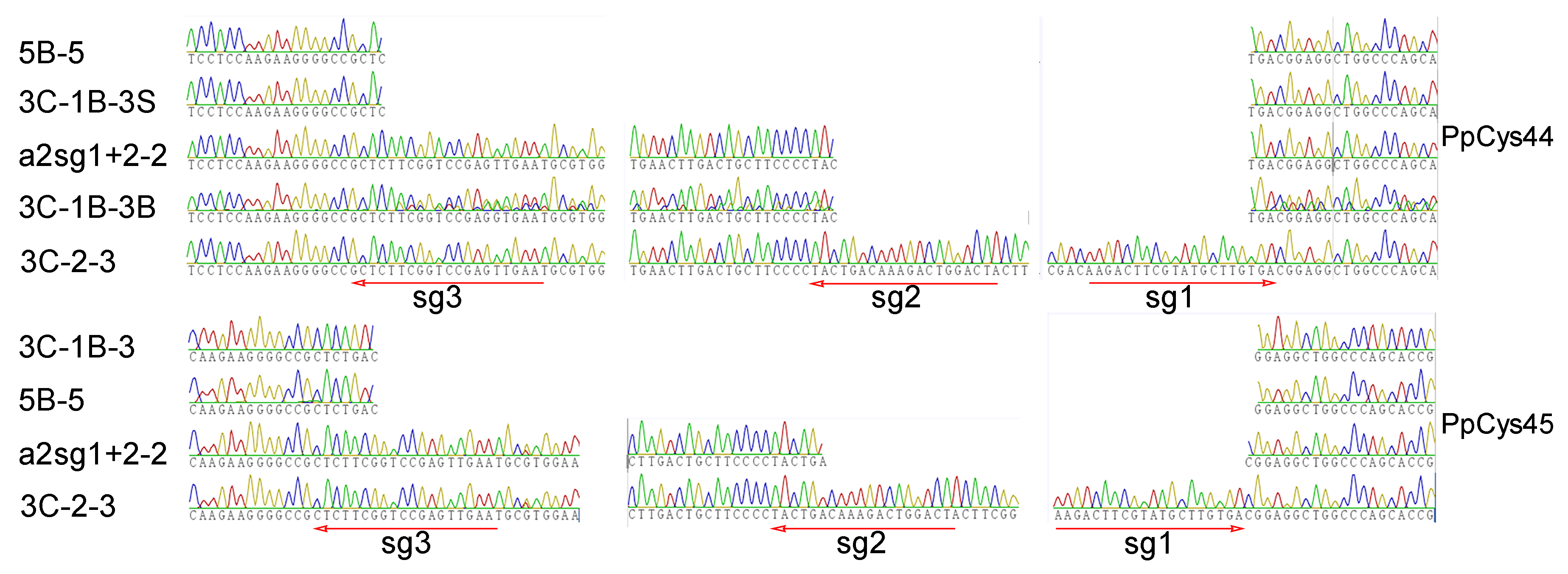

Supplement: Supplementary file 8 [file MPP-21-541-s008.tif]
