# Supplementary material for: Two Phytophthora parasitica cysteine protease genes, PpCys44 and PpCys45, trigger cell death in various Nicotiana spp. and act as virulence factors
Source: Mol Plant Pathol. 2020 Feb 19;21(4):541–54. doi: 10.1111/mpp.12915 (PMC7060141; doi:10.1111/mpp.12915)
Supplement: Supplementary file 9 [file MPP-21-541-s009.docx]

**Supporting information S9** All the primers used in this study.

| Name | sequence |
| --- | --- |
| **Primers for signal peptide function analysis** | |
| PpCys39sp-F | GGAATTTTAATTAAGAATTCATGTGGGGTGGACGCTTTGG |
| PpCys39sp-R | ACTATAGGGAGAACCTCGAGCTCAGCGTCGACAGGGTTGA |
| PpCys44/45sp-F | GGAATTTTAATTAAGAATTCATGAACACCGTGCTTCTGCTC |
| PpCys44/45sp-R | ACTATAGGGAGAACCTCGAGGGCCGCGTCTGCGGTG |
| PpCys43sp-F | GGAATTTTAATTAAGAATTCATGAACACCGCTCTTCTGCTC |
| PpCys43sp-R | ACTATAGGGAGAACCTCGAGTGCAGCGTCGGTAGCAGC |
| PpCys58sp-F | GGAATTTTAATTAAGAATTCATGCGCGTGCAAGCCCTC |
| PpCys58sp-R | ACTATAGGGAGAACCTCGAGAGCGGCCGAAGCGGAGG |
| PpCys59sp-F | GGAATTTTAATTAAGAATTCATGCAGATTCGAGCTATCACTTT |
| PpCys59sp-R | ACTATAGGGAGAACCTCGAGAGCACCATTGACAAGTGCTGT |
| PpCys60sp-F | GGAATTTTAATTAAGAATTCATGGTGCCGGCGCCGTG |
| PpCys60sp-R | ACTATAGGGAGAACCTCGAGTTGCGCCTGAGTCATCGAC |
| PpCys69sp-F | GGAATTTTAATTAAGAATTCATGAAGCCGATCACTTTAATCACAA |
| PpCys69sp-R | ACTATAGGGAGAACCTCGAGACGCGCATTGACAGGCACCA |
| PpCys70sp-F | GGAATTTTAATTAAGAATTCATGCACGTTCCCACTCTTTGC |
| PpCys70sp-R | ACTATAGGGAGAACCTCGAGTCGAGCAATGGTAGGAATAGTC |
| PpCys78sp-F | GGAATTTTAATTAAGAATTCATGCGCATTTTACGAGTTTTTCTACT |
| PpCys78sp-R | ACTATAGGGAGAACCTCGAGTACCGCTGCCGCGTCGTG |
| PpCys10sp-F | GGAATTTTAATTAAGAATTCATGAAGGTGTTCACGTCGCTG |
| PpCys10sp-R | ACTATAGGGAGAACCTCGAGCAGTGCACTGGCGGGGG |
| PpCys17sp-F | GGAATTTTAATTAAGAATTCATGCGGGCGTCTCTCATTG |
| PpCys17sp-R | ACTATAGGGAGAACCTCGAGCGAGGCCGATACGGTGG |
| PpCys19sp-F | GGAATTTTAATTAAGAATTCATGTTGCCCGTGTCCGTCT |
| PpCys19sp-R | ACTATAGGGAGAACCTCGAGCAAAGCCACGCCGGGGG |
| PpCys53sp-F | GGAATTTTAATTAAGAATTCATGCGCATCGCCTCCTCCT |
| PpCys53sp-R | ACTATAGGGAGAACCTCGAGGAGCGCGTCCGCCAGC |
| PpCys12sp-F | GGAATTTTAATTAAGAATTCATGGCGCTGTGGTCGTTCTT |
| PpCys12sp-R | ACTATAGGGAGAACCTCGAGACGAGCGCTGGCGGAAAC |
| PpCys20sp-F | GGAATTTTAATTAAGAATTCATGGGTTTTCTTGCTGCTTTATCC |
| PpCys20sp-R | ACTATAGGGAGAACCTCGAGCTCGGCATTGACCGGGGA |
| PpCys24sp-F | GGAATTTTAATTAAGAATTCATGGTCGGAGTACTGCGCG |
| PpCys24sp-R | ACTATAGGGAGAACCTCGAGCTTCGCCTCCACAAGCGAAG |
| PpCys35sp-F | GGAATTTTAATTAAGAATTCATGGCAAGGTGGACACTCTC |
| PpCys35sp-R | ACTATAGGGAGAACCTCGAGCTCCCCGCAAACGCAGCC |
| PpCys54sp-F | GGAATTTTAATTAAGAATTCATGACGCGGCGGAATCTGC |
| PpCys54sp-R | ACTATAGGGAGAACCTCGAGACTCTCGAAACGCTCTGA |
| PpCys65sp-F | GGAATTTTAATTAAGAATTCATGGTGCTGCTGTTGTTACTGC |
| PpCys65sp-R | ACTATAGGGAGAACCTCGAGGCAGGATCTCGCCAGTGG |
| PpCys67sp-F | GGAATTTTAATTAAGAATTCATGCAGCCCCGAGTGTCG |
| PpCys67sp-R | ACTATAGGGAGAACCTCGAGTCGCGCTTTGACATTATCCGAG |
| PpCys79sp-F | GGAATTTTAATTAAGAATTCATGAGCGACGACGGCAAGC |
| PpCys79sp-R | ACTATAGGGAGAACCTCGAGGTTCATGACCTCCGGGTTGG |
| **For 3xflag tagged protein construction** | |
| PpCys10-3xflag-F | AGAGGACACGCTCGAGATGAAGGTGTTCACGTCGCTG |
| PpCys10-3xflag-R | TCTTTGTAGTCCATCTCGAGCATGATGCTGGCCACCTCCGTC |
| PpCys17-3xflag-F | AGAGGACACGCTCGAGATGCGGGCGTCTCTCATTGT |
| PpCys17-3xflag-R | TCTTTGTAGTCCATCTCGAGGTGTGCATCGCCCACGTACA |
| PpCys53-3xflag-F | AGAGGACACGCTCGAGATGCGCATCGCCTCCTCCT |
| PpCys53-3xflag-R | TCTTTGTAGTCCATCTCGAGCATATACGAAATGGGTTCGTACAC |
| PpCys69-3xflag-F | AGAGGACACGCTCGAGATGAAGCCGATCACTTTAATCACAA |
| PpCys69-3xflag-R | TCTTTGTAGTCCATCTCGAGCATCTTCGGACGAGACATGTC |
| PpCys78-3xflag-F | AGAGGACACGCTCGAGATGCGCATTTTACGAGTTTTTCTACT |
| PpCys78-3xflag-R | TCTTTGTAGTCCATCTCGAGTTGTCCAGTTGTAGGACGCTG |
| PpCys60-3xflag-F | AGAGGACACGCTCGAGATGGTGCCGGCGCCGTG |
| PpCys60-3xflag-R | TCTTTGTAGTCCATCTCGAGCAGCGTCTCCTTGAAGGCG |
| PpCys39-3xflag-F | AGAGGACACGCTCGAGATGTGGGGTGGACGCTTTGG |
| PpCys39-3xflag-R | TCTTTGTAGTCCATCTCGAGCGGGATCGTCCGATACACG |
| PpCys58-3xflag-F | AGAGGACACGCTCGAGATGCGCGTGCAAGCCCTCA |
| PpCys58-3xflag-R | TCTTTGTAGTCCATCTCGAGGTGCGCCATTGTCTGCTGTG |
| PpCys39-3xflag-F | AGAGGACACGCTCGAGATGCAGATTCGAGCTATCACTTTC |
| PpCys39-3xflag-R | TCTTTGTAGTCCATCTCGAGATACCCATTTTGGGCATTCGTG |
| PpCys70-3xflag-F | AGAGGACACGCTCGAGATGCACGTTCCCACTCTTTGCT |
| PpCys70-3xflag-R | TCTTTGTAGTCCATCTCGAGCATCTGCGGAGTCGACATGTC |
| PpCys19-3xflag-F | AGAGGACACGCTCGAGATGTTGCCCGTGTCCGTCTTG |
| PpCys19-3xflag-R | TCTTTGTAGTCCATCTCGAGCATCGCGCAGTCCCGCTTG |
| PpCys12-3xflag-F | AGAGGACACGCTCGAGATGGCGCTGTGGTCGTTCTT |
| PpCys12-3xflag-R | TCTTTGTAGTCCATCTCGAGCATCTGCACGCTCCAAAAGTC |
| PpCys20-3xflag-F | AGAGGACACGCTCGAGATGGGTTTTCTTGCTGCTTTATCC |
| PpCys20-3xflag-R | TCTTTGTAGTCCATCTCGAGGGCCAACATGGCTACATTAGTG |
| PpCys24-3xflag-F | AGAGGACACGCTCGAGATGGTCGGAGTACTGCGCG |
| PpCys24-3xflag-R | TCTTTGTAGTCCATCTCGAGCTGCAGCGGTCTGTAGCC |
| PpCys35-3xflag-F | AGAGGACACGCTCGAGATGGCAAGGTGGACACTCTC |
| PpCys35-3xflag-R | TCTTTGTAGTCCATCTCGAGGATCGCTTTAATGGTTACGAACAC |
| PpCys54-3xflag-F | AGAGGACACGCTCGAGATGACGCGGCGGAATCTGC |
| PpCys54-3xflag-R | TCTTTGTAGTCCATCTCGAGGAAATCGTCGGGTTTCTCCCA |
| PpCys65-3xflag-F | AGAGGACACGCTCGAGATGGTGCTGCTGTTGTTACTGC |
| PpCys65-3xflag-R | TCTTTGTAGTCCATCTCGAGAGGAATGGGCAGGTACCTGG |
| PpCys67-3xflag-F | AGAGGACACGCTCGAGATGCAGCCCCGAGTGTCG |
| PpCys67-3xflag-R | TCTTTGTAGTCCATCTCGAGCACTTTGGGGTACGACATGTC |
| PpCys79-3xflag-F | AGAGGACACGCTCGAGATGAGCGACGACGGCAAGC |
| PpCys79-3xflag-R | TCTTTGTAGTCCATCTCGAGCTCGGTGGGCGTCTTCGC |
| P27:3xflag-F | AGAGGACACGCTCGAGATGGACTACAAAGACCATGATGGAGACTATAAGGATCACGACATCGATTACAAGGACGATGACGATAAG |
| P27:3xflag-R | TTAAAGCAGGACTCTAGTTATCTAGACTTATCGTCATCGTCCTTGTAATCGATGTCGTGATCCTTATAGTCTCCATCATGGTCTTTGT |
| PpCys44/45-3xflag-F | AGAGGACACGCTCGAGATGAACACCGTGCTTCTGCTC |
| PpCys44/45-3xflag-R | TCTTTGTAGTCCATCTCGAGCAGTTGAGGGTAGACGGACAA |
| PpCys43-3xflag-F | AGAGGACACGCTCGAGATGAACACCGCTCTTCTGCTC |
| PpCys43-3xflag-R | TCTTTGTAGTCCATCTCGAGCAGTTGAGGGTAGACGGACTT |
| PR1-SP-F | AGAGGACACGCTCGAATGGGATTTGTTCTCTTTTCACAA |
| PR1-SP-R | TTAAAGCAGGACTCTAGAGGATCCCTCGAGGGCACGGCAAGAGTGGGAT |
| PpCys44/45NC-3xflag-F | AGAGGACACGCTCGAGATGGCCGCTCCTGCTGTCCC |
| **For sgRNA design** | |
| sg1-F | ATTCGTTCGGTGACGCTAGCAGTCTTCTGATGAGTCCGTGAGGACGAAACGAGTAAGCTCGTCAAGACTTCGTATGCTTGTGA |
| sg1-R | CTATTTCTAGCTCTAAAACTCACAAGCATACGAAGTCTTGACGAGCTTACTCGTTTCGTCCTCACGGACTCATCAGAAGACTG |
| sg2-F | ATTCGTTCGGTGACGCTAGCGGACTACTGATGAGTCCGTGAGGACGAAACGAGTAAGCTCGTCTAGTCCAGTCTTTGTCAGTA |
| sg2-R | CTATTTCTAGCTCTAAAACTACTGACAAAGACTGGACTAGACGAGCTTACTCGTTTCGTCCTCACGGACTCATCAGTAGTCCG |
| sg3-F | ATTCGTTCGGTGACGCTAGCTTGAATCTGATGAGTCCGTGAGGACGAAACGAGTAAGCTCGTCATTCAACTCGGACCGAAGAG |
| sg-3R | CTATTTCTAGCTCTAAAACCTCTTCGGTCCGAGTTGAATGACGAGCTTACTCGTTTCGTCCTCACGGACTCATCAGATTCAAG |
| **For transformants detection** | |
| PpCys44-F | ATGAACACCGTGCTTCTGCTC |
| PpCys45-F | same as PpCys44-F |
| PpCys44-R | CACACCACCAGAGCCACCAA |
| PpCys45-R | GGTTGCCCAAGTGGAGGTGAT |
| **For RT-PCR** | |
| PpCys17q-F1 | GGTACTTGTAATGGTGGAAACCC |
| PpCys17q-R1 | TCGCAGATAGCGAACTGGTCG |
| PpCys43q-F | GCACCCGTGAAGCTGTGACTA |
| PpCys43q-R | GTTGAGTGCTGATGGCTTGGA |
| PpCys10q-F | TGATGAGCCGCAACAAGAACAA |
| PpCys10q-R | TCGTCTCCAGGACAATAGGG |
| PpCys53q-F | TTTCCAAGGACGAACAGACCG |
| PpCys53q-R | GAGGAGAACAGATCAGCCAAGT |
| PpCys69q-F | CGCTTACGAGTACATCCAGAAGA |
| PpCys69q-R | AGCCTGTGATTCCCGTTTGAC |
| PpCys78q-F | CTGTTACTGCCAACATTGACGC |
| PpCys78q-R | CGAAGCCCACGACTACCACA |
| PpCys60q-F | CGGGACCTTCTTGCGATGAGA |
| PpCys60q-R | CACGTATAAATACCGCCCTTGT |
| PpCys39q-F | GAGGGTCTATCTTCGGCATCG |
| PpCys39q-R | CGTCTCCGGCAAGTAATGAGC |
| PpCys58q-F | GGGGATAAGGTCTTTGTTGGTCT |
| PpCys58q-R | ACTTGAGCTTCCGCTGTGAGAT |
| PpCys59q-F | CTTACAATGCCATACGACGACTC |
| PpCys59q-R | ACGATCTGCTCCCAACCAGTA |
| PpCys70q-F | AACGGGTTGAATACGGCTACAA |
| PpCys70q-R | CGTTAGTCGTCGGTTGGAGT |
| PpCys19q-F | GAGGAGGGCTACGTTCGTGT |
| PpCys19q-R | CGGAAGGAGCAGAGGTGGT |
| PpCys12q-F | TCACGATGAACAACCACGAACA |
| PpCys12q-R | CAAAAGCTGAGATAAACTCAACTCCT |
| PpCys20q-F | CGACGACTCTGACAGCGAAGA |
| PpCys20q-R | CGCAACGACCAGGTCCACA |
| PpCys24q-F | ATGACCAAGGGACCGAAGAGC |
| PpCys24q-R | TCGAAGTGACCACGATCAATGTC |
| PpCys35q-F | ACTCCTGACTACCGGACTGAC |
| PpCys35q-R | TCGTCGTCGAAGTGAAGATGC |
| PpCys65q-F | TATGTGCCATTCTCCTGTGCG |
| PpCys65q-R | CCTACTAATAGCGTCAAAGCGTA |
| PpCys67q-F | AGTTGCTGGGCGTTCGCTAG |
| PpCys67q-R | CAAGATGCTGTTCCGAGTATGAG |
| PpCys44/45q-F | ACGCAGTGGGGCGAGGAT |
| PpCys44/45q-R | TGAGGGTAGACGGACAAGGG |
